# A probabilistic multi-omics data matching method for detecting sample errors in integrative analysis

**DOI:** 10.1093/gigascience/giz080

**Published:** 2019-07-09

**Authors:** Eunjee Lee, Seungyeul Yoo, Wenhui Wang, Zhidong Tu, Jun Zhu

**Affiliations:** 1 Department of Genetics and Genomic Sciences, Icahn School of Medicine at Mount Sinai, One Gustave L. Levy Place, New York, NY 10029, USA; 2 Icahn Institute of Genomics and Multiscale Biology, Icahn School of Medicine at Mount Sinai, One Gustave L. Levy Place, New York, NY 10029, USA; 3 Sema4, a Mount Sinai venture, 333 Ludlow street, Stamford, CT 06902, USA; 4 The Tisch Cancer Institute, Icahn School of Medicine at Mount Sinai, One Gustave L. Levy Place, New York, NY 10029, USA

**Keywords:** data error, omics data integration, data curation

## Abstract

**Background:**

Data errors, including sample swapping and mis-labeling, are inevitable in the process of large-scale omics data generation. Data errors need to be identified and corrected before integrative data analyses where different types of data are merged on the basis of the annotated labels. Data with labeling errors dampen true biological signals. More importantly, data analysis with sample errors could lead to wrong scientific conclusions. We developed a robust *probabilistic* multi-omics data matching procedure, proMODMatcher, to curate data and identify and correct data annotation and errors in large databases.

**Results:**

Application to simulated datasets suggests that proMODMatcher achieved robust statistical power even when the number of *cis*-associations was small and/or the number of samples was large. Application of our proMODMatcher to multi-omics datasets in The Cancer Genome Atlas and International Cancer Genome Consortium identified sample errors in multiple cancer datasets. Our procedure was not only able to identify sample-labeling errors but also to unambiguously identify the source of the errors. Our results demonstrate that these errors should be identified and corrected before integrative analysis.

**Conclusions:**

Our results indicate that sample-labeling errors were common in large multi-omics datasets. These errors should be corrected before integrative analysis.

## Background

With advances in high-throughput technologies in the past 2 decades, diverse types of omics data at multiple layers of regulation have been generated to survey complex human diseases [1–3], which arise from dysregulations of interplays among these multiple layers of regulations including genetics, epigenetics, transcriptomics, metabolomics, glycomics, and proteomics. Therefore, integration of multi-omics data at multiple layers of regulation is essential to derive a holistic view of molecular mechanisms underlying complex human disease. Previous studies have shown that simultaneously considering diverse types of biological data results in more complete understandings of biological systems [4–6].

Recently, many large projects, such as The Cancer Genome Atlas (TCGA) and International Cancer Genome Consortium (ICGC), have generated diverse types of omics data for public use. However, data errors, including sample swapping, mis-labeling, and improper data entry, are almost inevitable in the process of large-scale data generation and management. Westra et al. [7] found ∼20% of mis-matched samples between genotype and gene expression data. Yoo et al. [8] demonstrated that sample-labeling errors occurred in almost every database examined. Also, there are studies to identify cross-individual contamination in next-generation sequencing data from TCGA samples [9,10].

Identifying and ultimately correcting these sample errors are critical for statistical data analysis, especially for integrative analysis. Data errors need to be identified and corrected before extensive efforts are devoted to data analysis. Analyzing data with sample errors is a waste of limited public resources. More importantly, data analysis with sample errors could lead to wrong scientific conclusions. Furthermore, sample errors have more significant effects on integrative data analysis where different types of data are merged on the basis of the annotated labels. Some types of sample errors can be detected during data quality control on each individual type of data, whereas such sample errors as sample swapping or mis-labeling are difficult to detect by means of data quality control on that individual type of data alone.

Previously, we developed a sample-mapping procedure called MODMatcher (Multi-Omics Data matcher) [8], which is not only able to identify mis-matched omics profile pairs but also to properly map them to correct samples based on other omics data. The main idea is first to identify “biological *cis*-associations” between 2 types of omics data and then to use these biological *cis*-associations as intrinsic barcodes to match different types of omics data. The types of biological *cis*-associations are different when different pairs of omics data are mapped, but they all reflect general biological regulations. For example, when mapping genotype and gene expression data, the method is based on *cis*-genetic regulation of expression traits (or expression quantitative trait loci—*cis*-eQTLs), where a genetic polymorphism at a gene's promoter or regulatory region affects the binding of transcription factors or co-factors, which in turn affects the abundance of the gene's transcript [11]. Similarly, when mapping methylation and gene expression data, the method leverages on *cis*-methylation regulation of expression traits (or *cis*-methyls), where a high DNA methylation level of CpGs at a gene's promoter or regulatory region hinders the binding of transcription factors or co-factors, which in turn represses the gene's transcription [12]. More details on biological *cis*-associations are provided in the Methods section.

We demonstrated that the statistical power to identify biological signals increases after database cleaning by applying the MODMatcher procedure to multiple large-scale public multi-omics datasets from the Lung Genomic Research Consortium and TCGA. The power of MODMatcher depends on the number of intrinsic biological *cis*-associations that can be identified. The power of MODMatcher decreases when the number of *cis*-associations between 2 omics profiles is small. However, in some cases (a few examples are detailed in the Results), the number of possible intrinsic biological *cis*-associations is small, and new methods are needed for these types of applications.

In this study, we extended MODMatcher and developed a robust *probabilistic* multi-omics data-matching procedure, proMODMatcher, to curate data and identify and unambiguously correct data annotation and metadata attribute errors in large databases. First, we applied the proMODMatcher to simulated datasets to assess the statistical power of our procedure. The results suggest that proMODMatcher achieved robust statistical power even when the number of *cis*-associations was small and/or the number of samples was large. Next, we applied the proMODMatcher procedure to multiple large-scale publicly available multi-omics datasets from TCGA and, in particular, focused on the omics profiles that have small numbers of intrinsic *cis*-associations including microRNA (miRNA) expression and reverse phase protein array (RPPA). Additionally, we applied proMODMatcher to large-scale publicly available multi-omics datasets in ICGC. Our results indicate that sample-labeling errors were common in large multi-omics datasets. These errors should be corrected before integrative analysis is performed.

## Data Description

### TCGA datasets

For the TCGA breast invasive carcinoma (BRCA) dataset, level 3 data of gene expression, DNA methylation, miRNA expression, and copy number variation (CNV) was downloaded from the Genomic Data Commons data portal [13]. For gene expression profiles, the IlluminaHiSeq_RNASeqV2 and AgilentG4502A platform were used. Illumina HumanMethylation27 (HM27) and HumanMethylation450 (HM450) Beadchip were used for DNA methylation bisulfide sequencing. The IlluminaHiSeq_miRNASeq and IlluminaGA_miRNASeq platforms were used to profile miRNA expression. Affymetrix Genome-Wide Human SNP Array 6.0 was used for CNV. The protein expression levels were measured in RPPA and downloaded. Each of the level 3 profiles was reformatted into a matrix with genes (or probes) as rows and barcodes of samples as columns. For methylation profiles and CNV, the probes or segments were mapped to hg19 gene symbols. Different profiles were initially matched according to their barcodes. The mapping files of HM450, RPPA, and miRNA are available in the source code.

For other types of cancers in TCGA, we downloaded gene expression, miRNA expression, CNV, DNA methylation, and RPPA data from the Firehose database [14]. For RPPA data, we filtered genes with >25% of samples with not-assigned measurements.

### ICGC datasets

For the ICGC datasets, the pre-processed data were downloaded from the ICGC data portal [15]. We selected datasets with >1 available types of omics data including mesenger RNA (mRNA) expression profiles (i.e., RNA sequencing [RNAseq] and Array), DNA methylation profiles based on Illumina HM450, miRNA expression profiles, and copy number somatic mutation profiles. Each of the profiles was reformatted into a matrix with genes (or probes) as rows and barcodes of samples as columns. The gene and miRNA expression profiles were log_2_ transformed and normalized by quantile normalization [16]. For copy number somatic mutation profiles, the segments were mapped to hg19 gene symbols. Some datasets contain very sparse segment information for copy number somatic mutation profiles such as CLLE-ES. We excluded these copy number profiles from further analysis. For methylation profiles, the probes were mapped to hg19 gene symbols.

### Simulation study

Simulated datasets for testing alignment between a pair of omics profiles were generated. Given a set of *N cis*-associations, each of correlation coefficient *r_n_*, we can simulate omics profiles Υ based on omics profiles *X* for *M* samples as follows: ${X_i} = \ N( {0,1} )$ is a standard normal distribution, and ${Y_i} = \frac{{{r_n}}}{{\sqrt {1 - r_n^2} }}\ {X_i} + \epsilon ,$ where $\epsilon $ is a standard normal distribution, $N( {0,1} )$. For each *N* and *M* combination, we simulated *N* significant sets with *r_n_* drawn from a truncated normal distribution with a cut-off value corresponding to correlation coefficients *q*-value < 0.05, as well as 2,000 sets of random *r_n_* drawn from a normal distribution. We considered *N* significant *cis*-associations from 75 through 1000, and *M* samples from 100 through 1,000. The simulated data with label error were generated by permuting the labels of 1 type of data. We considered 0, 2, ..., 10% label error rates. We measured sensitivity (i.e., recall) = number of truly aligned pairs divided by number of simulated pairs, specificity (i.e., precision) = number of truly aligned pairs divided by number of aligned pairs, false-positive rate (FPR) = 1 − specificity, and F measures (= 2 × [(precision × recall)/(precision + recall)]) for assessment. Additionally, because a pair of omics profiles mostly has unbalanced samples, we mimic this by adding 10% of *M* samples for Type A and Type B omics profiles.

## Analyses

### Overview of proMODMatcher procedure

**Figure 1.**
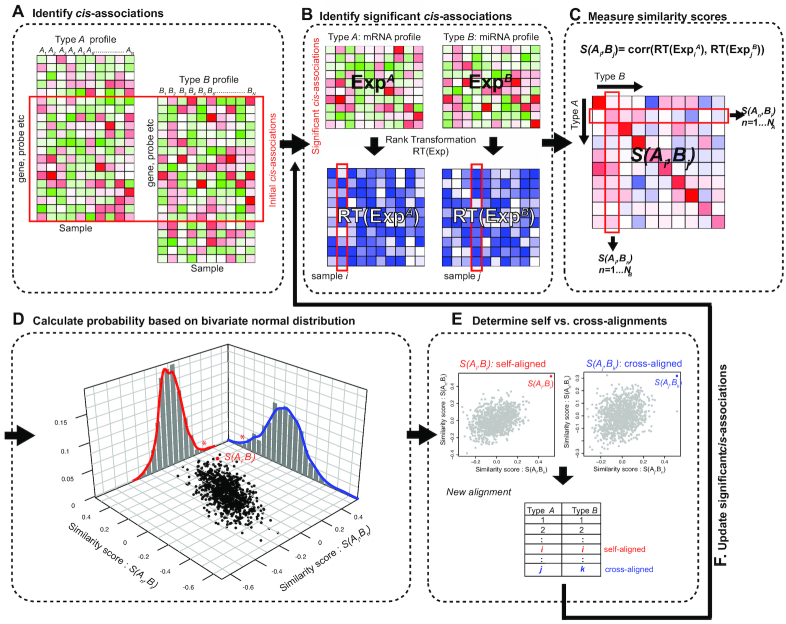
Overview of proMODMatcher procedure. **(A)** Probes in 2 types of profiles (i.e., Type A and Type B) were matched by intrinsic biological relationships. **(B)** The significant *cis*-associations from 2 different data types were identified by the Spearman correlation. The data for each *cis* relationship were normal rank-transformed. (**C**) The sample similarity score between the 2 types of data $S( {{A_i},\ {B_j}} )$ is defined as the Spearman correlation between normal rank-transformed profiles. **(D)** The proMODMatcher evaluated the similarity score of a match, $\ S( {{A_i},\ {B_j}} )$, by calculating the probability of a match estimated on the basis of a score distribution of $\ ( {S( {{A_i},\ {B_n}} ),\ S( {{A_n},\ {B_j}} )} )$, where *A_n_* and *B_n_* represent Type A and Type B profile of the *n*^th^ matched profile pairs. **(E)** In the determine self-aligned vs cross-aligned step, profile pairs matched by sample IDs were checked to determine whether their similarity scores were high, in which case they would be annotated as “self-aligned.” If not, additional steps were applied to find any potential matches among other unmatched profiles. The matched profile pairs were used to update significant *cis*-associations.

proMODMatcher followed the general framework of multi-omics data matching of the previous study [8]. Two types of data (or profiles) (i.e., Type A and Type B in Fig. 1) were matched based on their *cis*-associations. Samples were initially matched based on annotated sample ID and potential *cis*-associations (Fig. 1A). The significant *cis*-associations from 2 different data types were identified by the Spearman correlations (Fig. 1B). The data for each *cis*-association were normal rank-transformed (Fig. 1B). The profile similarity between the 2 types of data $S( {{A_i},\ {B_j}} )$ is defined as the correlation between profile *i* of Type A and profile *j* of Type B (Fig. 1C). The probability of a match between profile *i* of Type A and profile *j* of Type B is estimated by evaluating a similarity score in a bivariate normal distribution (Fig. 1D). Based on probability of a match, proMODMatcher determines self- or cross-alignments for each match. First, profile pairs matched by annotated sample IDs were checked to determine whether their similarity scores were high (Fig. 1D), in which case they would be annotated as “self-aligned.” If not, additional steps were applied to find any potential matches among other unmatched profiles (Fig. 1E). The matched profile pairs were then used to update significant *cis*-associations. We iteratively refined profile alignment, and rounds of alignments were repeated until there were no further updates (Fig. 1F).

### Simulation studies

**Figure 2.**
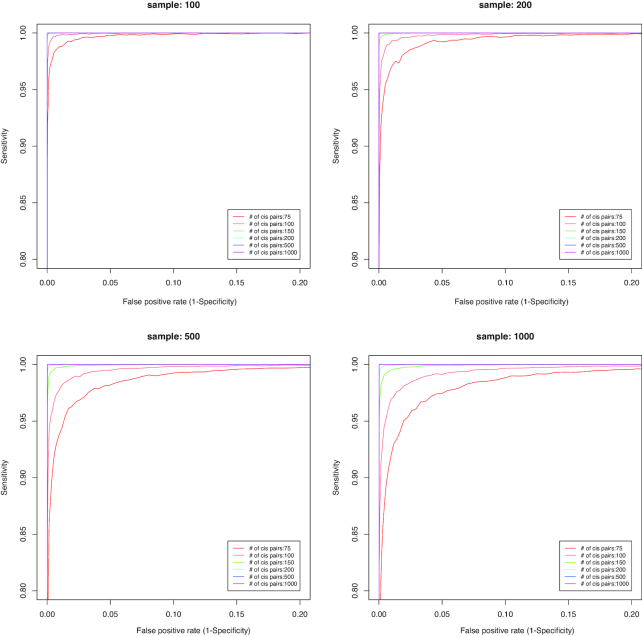
Application of MODMatcher to simulated datasets. We simulated datasets with different numbers of samples and significant *cis*-associations. For variable number of samples and significant *cis*-associations, sensitivity and false-positive rate (FPR, 1 − specificity) were measured and plotted.

Numbers of significant *cis*-associations and samples are 2 important deterministic factors of similarity scores, as well as the accuracy of omics profile alignment results. To investigate the effect of numbers of samples and *cis*-associations, we simulated datasets with different numbers of samples and significant *cis*-associations and applied MODMatcher and proMODMatcher to the simulated datasets. For MODMatcher, when the number of *cis*-associations was >200, almost all profile pairs could be aligned at high accuracy (FPR vs sensitivity) (Fig. 2). The similarity scores of matched pairs based on a low number of *cis*-associations were more variable, resulting in lower accuracies (Supplementary Fig. S1). This result indicates that the MODMatcher can be applied to align omics profile pairs with >200 *cis*-associations, such as methylation-mRNA profiles with >7,000 intrinsic *cis*-associations and mRNA-CNV profiles with >10,000 intrinsic *cis*-associations [8]. On the other hand, when the number of *cis*-associations was ∼200 or less, the accuracy of sample alignments decreased as the number of samples increased (Fig. 2). When aligning gene expression profiles with miRNA or RPPA profiles, the number of candidate intrinsic *cis*-associations was small (detailed below). Thus, MODMatcher was not powered to accurately align these types of profile pairs.

**Figure 3.**
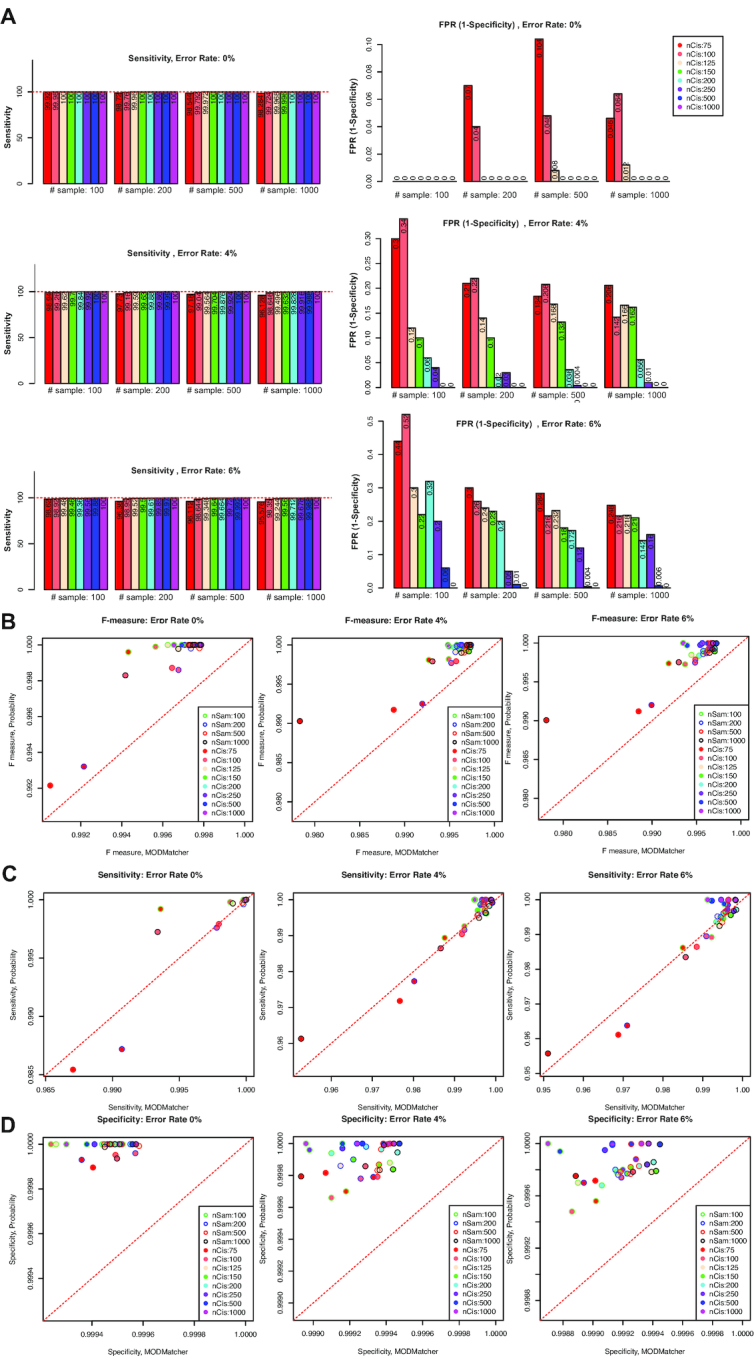
Application of proMODMatcher to simulated datasets. **(A)** For variable number of samples and significant *cis*-associations specificity and FPR were measured on the basis of simulated datasets with 0%, 4%, and 6% sample-labeling error rate. **(B,C)** F measure, sensitivity, and specificity were compared with MODMatcher's results.

The proMODMatcher was applied to the same simulated datasets and was able to achieve high sensitivities and low FPRs across a wide range of numbers of *cis*-associations and samples (Fig. 3A). When compared with MODMatcher's results, proMODMatcher resulted in better accuracies (F measure in Fig. 3B), similar sensitivities (Fig. 3C), and better specificities (Fig. 3D).

We further investigated their performances when there were labeling errors. Datasets with sample-labeling errors (i.e., 4% and 6%) were simulated by randomly assigning some samples’ labels; then proMODMatcher and MODMatcher were applied to identify aligned profile pairs. As expected, when a larger number of *cis*-associations was available, proMODMatcher achieved a high sensitivity and low FPR (Fig. 3A). Across all tested combinations of numbers of *cis*-associations and samples, proMODMatcher resulted in >99% accuracy with 4–6% input labeling error rates, consistently outperforming MODMatcher (Fig. 3B). The top goal of MODMatcher and proMODMatcher is to identify sample-labeling errors without introducing any errors. Thus, we optimized the specificity of proMODMatcher over its sensitivity. In terms of the contribution of sensitivity and specificity to F scores, proMODMatcher achieved a similar sensitivity as MODMatcher (Fig. 3C) but better specificities in all cases (Fig. 3D). These simulation results suggest that proMODMatcher is applicable for identifying and correcting labeling errors even when the number of *cis*-associations is small such as pairing mRNA-miRNA or mRNA-RPPA profiles.

### Application to TCGA breast cancer dataset: mRNA and miRNA profiles

**Figure 4.**
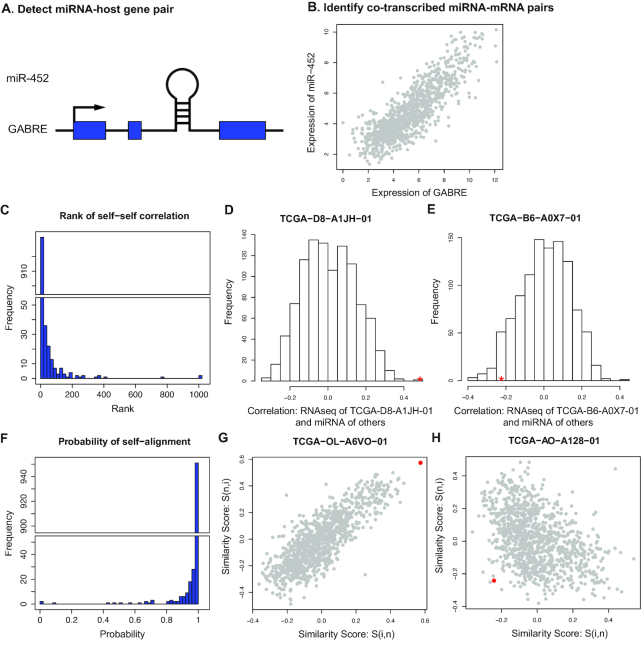
Aligning gene expression profiles by RNAseq and miRNAseq data. **(A)** An example of miRNAs (e.g., miR-452) that are embedded in gene regions (e.g., *GABRE*). **(B)** Expression level of miR-452 was highly associated with mRNA expression of *GABRE*. **(C)** The rank of the similarity scores of self-self RNAseq-miRNAseq profiles. **(D)** An example of the similarity score of the self-aligned profiles, TCGA−D8−A1JH-01. The similarity score between the RNAseq profile of TCGA−D8−A1JH-01 and the miRNA profiles of other samples are shown. The red asterisk indicates the similarity score of self-self RNAseq-miRNAseq profiles. **(E)** An example of non–self-aligned RNAseq-miRNA profiles, TCGA-B6-A0X7-01. **(F)** The probabilities of similarity scores (before multiplying prior probability) for self-aligned RNAseq-miRNAseq profiles. **(G)** An example of similarity scores of self-aligned RNAseq-miRNA profile pairs. The x-axis indicates the similarity scores between the RNAseq profile of TCGA-OL-A6VO-01 and the miRNAseq profiles of all other samples, and the y-axis indicates similarity scores between the miRNAseq profile of TCGA-OL-A6VO-01 and the RNAseq profiles of all other samples. The red dot indicates the similarity score for the self-self RNAseq-miRNAseq profile. **(H)** An example of similarity scores of non–self-aligned RNAseq-miRNA profile pairs.

Multiple omics data, including profiles of mRNA, miRNA, protein, DNA methylation, and CNV, were available in TCGA. The proMODMatcher was applied to align methylation and/or CNV profiles to mRNA profiles, similar to what we did previously [8]. Here we focused on alignment of miRNA expression profiles to mRNA expression data because the number of candidate intrinsic *cis*-associations between miRNA and mRNA profiles was small. We used the TCGA breast cancer (BRCA) dataset as an example to illustrate the profile alignment results in detail. There were mRNA expression profiles based on 2 different platforms, Agilent microarray and RNAseq technology. There were 519 tumor samples with both mRNA expression measured in Agilent microarray and miRNA expression measured by small-RNA sequencing method, and 1,041 tumor samples with both mRNA expression measured in RNAseq and miRNA measured by small-RNA sequencing method. A small portion of miRNAs are embedded in gene regions (i.e., host genes) and frequently co-transcribed with host genes [17, 18] (Fig. 4A); embedded miRNA-host gene pairs were candidate intrinsic *cis*-associations. A total of 1,222 miRNAs were profiled, and 227 and 271 of them were mapped to host genes, for Agilent microarray and RNAseq data, respectively. Among them, 138 of 227 and 175 of 271 miRNA-host gene pairs were significantly associated with each other at *q*-value < 0.05, for Agilent microarray and RNAseq data, respectively. For example, in the case of miR-452 located in the gene body of *GABRE*, its expression was highly associated with mRNA expression of *GABRE* (Fig. 4B). On the basis of these intrinsic *cis*-associations between expression levels of miRNAs and host genes, we aligned the 2 types of omics data.

#### Aligning gene expression profiles by RNAseq and miRNAseq data

The similarity scores of self-aligned gene expression–miRNA expression profiles were much higher than other possible pairings in general (Fig. 4C): 898 of 1,041 (86.3%) similarity scores for self-self RNAseq-miRNAseq profiles were ranked in the top 2%. For example, the similarity score for the self-aligned profiles of TCGA−D8−A1JH-01 was top ranked among other possible pairings (Fig. 4D). A total of 143 miRNA profiles that were not matched to the corresponding mRNA profiles of the same sample names based on MODMatcher (e.g., TCGA−B6−A0X7-01 shown in Fig.   4E). Among profile pairs that were not self-aligned, 5 RNAseq profiles were cross-aligned to other samples’ miRNA profiles (Supplementary Table S1). The rate of alignment was low compared to alignments of other types of profile pairs. For example, >99% of the profile pairs of DNA methylation and mRNA expression profiles were aligned for the TCGA BRCA dataset.

When proMODMatcher was applied to TCGA BRCA RNAseq-miRNAseq datasets, the probabilities of similarity scores (before multiplying prior probability) for self-aligned RNAseq-miRNA profiles were much higher than for other possible pairs in general (Fig. 4F). An example of similarity scores of a self-aligned RNAseq-miRNA profile pair and other possible pairs is shown in Fig. 4G. There were multiple self-self pairs with low probabilities for self-alignment (Fig. 4F and H), suggesting potential labeling errors in RNAseq and/or miRNA profiles. Overall, 989 of 1,041 candidate matching pairs (95.0%) (Table 1) were self-aligned compared to 86.3% for MODMatcher. Among profiles that were not self-aligned, 1 profile pair (i.e., TCGA-BH-A0BZ-01 and TCGA-E2-A15K-01) was cross-aligned to each other (Table 1).

**Table 1.** Application of proMODMatcher to mRNA and miRNA profiles of TCGA BRCA data

Data type Type 1	Type 2	No. samples^1^	No. *cis* pair^2^	No. (%) self-aligned	No. cross	Cross-aligned pairs RNA-CNV^3^Type 1	Self-aligned	Cross-aligned pairsType 2	By MODMatcher^4^
RNAseq	miRNAseq	1,041	175/215	989 (95.0)	1	**TCGA-BH-A0BZ-01**	**Y**	**TCGA-E2-A15K-01**	**Y**
Agilent	miRNAseq	519	138/178	466 (89.8)	9	TCGA-A8-A07U-01	Y	TCGA-A2-A3XY-01	Y
						TCGA-BH-A0H9-01	Y	TCGA-EW-A423-01	No
						TCGA-AO-A128-01	Y	TCGA-BH-A18V-06	Y
						**TCGA-A1-A0SD-01**	**No: TCGA-BH-A0EI-01**	**TCGA-BH-A0EI-01**	**Y**
						TCGA-BH-A18K-01	No: TCGA-BH-A18T-01	TCGA-BH-A18T-01	Y
						TCGA-BH-A18T-01	No: TCGA-BH-A18K-01	TCGA-BH-A18K-01	Y
						**TCGA-BH-A0BZ-01**	**Y**	**TCGA-E2-A15K-01**	**Y**
						**TCGA-BH-A0BS-01**	**No: TCGA-BH-A0BT-01**	**TCGA-BH-A0BT-01**	**Y**
						TCGA-AR-A0U0-01	Y	TCGA-AR-A256-01	Y

Boldface indicates cross-alignments supported by other data, and underscore indicates sample swaps.

^1^The number of common samples with both Type 1 and Type 2 profiles.

^2^The number of significant *cis*-pairs at *q*-value < 0.05 at final iteration and the number of *cis*-pairs investigated.

^3^Indicates whether the RNA samples of cross-aligned pairs were self-aligned in alignment between RNA profile (Agilent array or RNAseq) and CNV profile. The aligned pairs were also shown if there was a cross-aligned sample.

^4^Indicates whether the cross-aligned pairs were cross-aligned by MODMatcher.

**Figure 5.**
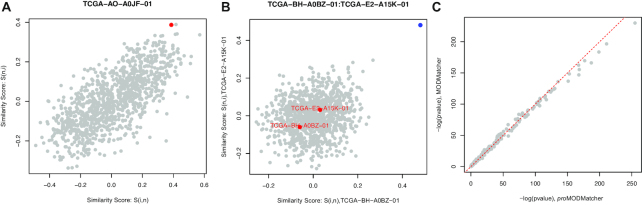
Comparison of MODMatcher and proMODMatcher for aligning expression profiles by RNAseq and miRNAseq data. **(A)** The similarity scores of a self-aligned RNAseq-miRNA profile pair identified by proMODMatcher but not by MODMatcher. The x-axis indicates the similarity score between the RNAseq profile of TCGA-AO-A0JF-01 and the miRNAseq profiles of all other samples, and the y-axis indicates the similarity score between the miRNAseq profile of TCGA-AO-A0JF-01 and the RNAseq profiles of all other samples. The red dot indicates the similarity score for self-self RNAseq-miRNAseq profiles. **(B)** One cross-aligned pair, RNAseq of TCGA-BH-A0BZ-01 and miRNA of TCGA-E2-A15K-01, identified by proMODMatcher. The similarity score of the cross-aligned pair is shown in blue, and the similarity scores of self-self alignments are shown in red. **(C)** Significance levels of *cis*-associations based on profile pairs aligned by MODMatcher and proMODMatcher.

Comparing MODMatcher and proMODMatcher, the proMODMatcher identified an additional 91 self-aligned profile pairs that were missed by MODMatcher. For example, the similarity score of self-alignment for TCGA-AO-A0JF-01 was among the highest when the miRNA profile was compared to RNAseq profiles of other samples (y-axis in Fig. 5A). However, the RNAseq profile of TCGA-AO-A0JF-01 was highly similar to multiple miRNA profiles of other samples (x-axis in Fig. 5A). As a result, the rank-based MODMatcher rejected the self-alignment, but proMODMatcher identified self-alignment for TCGA-AO-A0JF-01 with *P*-value of 7.3 × 10^−6^.

One cross-aligned pair, RNAseq of TCGA-BH-A0BZ-01 and miRNA of TCGA-E2-A15K-01, was identified by both proMODMatcher and MODMatcher. The similarity score of the cross-aligned pair is shown in Fig. 5B. The similarity scores of self-self alignments were low (red dots in Fig. 5B); on the other hand, the similarity score of the cross-aligned pair was significantly higher compared to other similarity scores (Fig. 5B), indicating high confidence of cross-alignment. On the other hand, the cross-aligned pairs detected only by MODMatcher showed relatively marginal similarity scores even though the similarity scores of cross-aligned pairs were the highest (Supplementary Fig. S2). Furthermore, we compared significance levels of *cis*-associations based on profile pairs aligned by MODMatcher and proMODMatcher. They were comparable in general, with a few highly significant *cis*-associations that were more significant based on proMODMatcher compared to MODMatcher (Fig. 5C).

#### Aligning gene expression profiles by Agilent microarray and miRNAseq data

MODMatcher and proMODMatcher were also applied to align mRNA expression profiles based on Agilent microarray and miRNA profiles. There were 138 *cis*-associations identified on the basis of Agilent microarray data and miRNAseq data. On the basis of these *cis*-associations, 87.1% of candidate profile pairs were identified as self-aligned by MODMatcher (Supplementary Table S1) while 89.8% of candidate profile pairs were self-aligned by proMODMatcher (Table 1).

**Figure 6.**
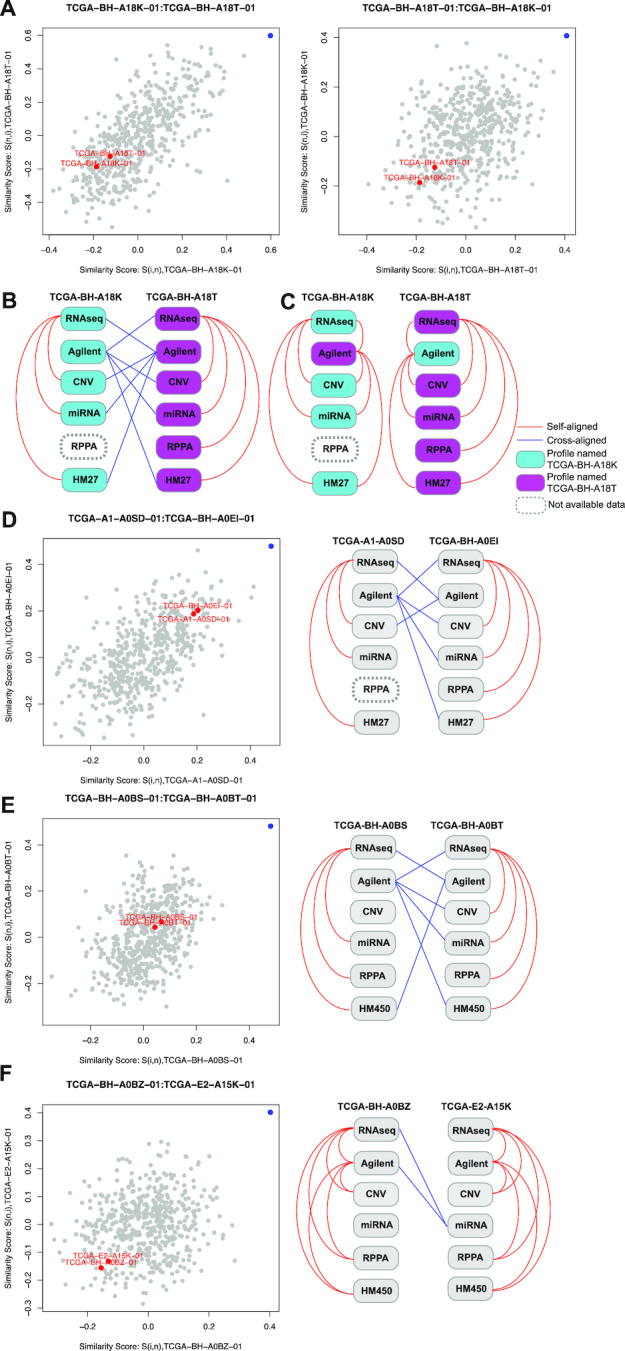
Aligning gene expression profiles by Agilent array and miRNAseq data. **(A)** An example of possible sample swaps. In the alignment of the Agilent array and miRNAseq profiles, TCGA-BH-A18K-01 and TCGA-BH-A18T-01 were cross-aligned to each other. The similarity scores of each cross-alignment are shown. The similarity score of the cross-aligned pair is shown in blue, and the similarity scores of self-self alignments are shown in red. **(B)** Other omics profiles of TCGA-BH-A18K and TCGA-BH-A18T were compared with each other, and the results were summarized into a patient-centric view. Red line indicates self-aligned, and blue line indicates cross-aligned. **(C)** After swapping the corresponding mRNA Agilent array profiles, multiple-omics profiles of TCGA-BH-A18K and TCGA-BH-A18T were aligned to each other consistently. **(D–F)** The similarity scores of other cross-aligned pairs are shown, and their available omics profiles and alignment results are summarized into a patient-centric view.

Among profiles that were not self-aligned, 9 cross-aligned profile pairs were identified by proMODMatcher (Table 1, Supplementary Fig. S3B), and 8 of the 9 pairs were also detected by MODMatcher (Table 1). MODMatcher detected additional cross-aligned pairs including several questionable cross-aligned pairs (i.e., TCGA−E2−A153−01 and TCGA−E9−A1NG−01, TCGA-AR-A1AL−01 and TCGA−AR−A1AN−01 in Supplementary Fig. S4). The cross-aligned pairs identified by proMODMatcher included a possible swap between TCGA-BH-A18**K**-01 and TCGA-BH-A18**T**-01 (Fig. 6A and Table 1). To determine the source of labeling errors (due to mRNA Agilent profiles or miRNA profiles) other omics profiles were compared with each other and the results were summarized into a patient-centric view (Fig. 6B). For patient/sample TCGA-BH-A18**K,** the RNAseq and miRNAseq profiles were self-aligned and the RNAseq and CNV profiles were self-aligned as well (Fig. 6B). Similarly, for patient/sample TCGA-BH-A18**T,** the RNAseq profile was self-aligned to the miRNA, CNV, and DNA methylation profiles as well as the RPPA profile (detailed below) (Fig. 6B). The cross-alignments of TCGA-BH-A18K-01 and TCGA-BH-A18T-01 mRNA Agilent profiles with their miRNA profiles (Fig. 6B) indicate that sample swapping occurred in the mRNA Agilent array profiles. After swapping the corresponding mRNA Agilent array profiles, multiple-omics profiles of TCGA-BH-A18K and TCGA-BH-A18T were aligned to each other consistently (Fig. 6C). Our previous study based on pairwise profile alignments of gene expression, DNA methylation, and CNV also identified the sample swaps in the mRNA Agilent array profiles of TCGA-BH-A18K-01 and TCGA-BH-A18T-01 [8] (Fig. 6B and C). In addition, proMODMatcher identified a cross-alignment of the mRNA Agilent array profile of TCGA-A1-A0SD-01 and the miRNA profile of TCGA-BH-A0EI-01 (Table 1, Fig. 6D), consistent with potential sample swaps of mRNA Agilent array profiles of TCGA-A1-A0SD-01 and TCGA-BH-A0EI-01 when alignments of other omics profiles were included. Similarly, the cross-alignment between the Agilent array profile of TCGA-BH-A0B**S**-01 and the miRNA profile of TCGA-BH-A0B**T**-01 was likely a result of a swap between the Agilent array profiles of the 2 samples when all available omics data were added into the comparison (Fig. 6E).

The proMODMatcher identified a cross-aligned pair between the mRNA Agilent array profile of TCGA-BH-A0BZ-01 and the miRNA profile of TCGA-E2-A15K-01 (See Table 1, Fig. 6F). The miRNA profile of TCGA-E2-A15K-01 was also cross-aligned to the mRNAseq profile of TCGA-BH-A0BZ-01 (Table 1, Fig. 5B). When alignments of other omics profiles were included in a patient-centric view (Fig. 6F), the result suggests that there was a labeling error of the miRNA profile of TCGA-E2-A15K-01.

These results together suggest that proMODMatcher with 138 *cis*-associations can accurately identify sample-labeling errors and unambiguously correct labeling errors.

### Application to TCGA breast cancer dataset: mRNA and RPPA profiles

**Figure 7.**
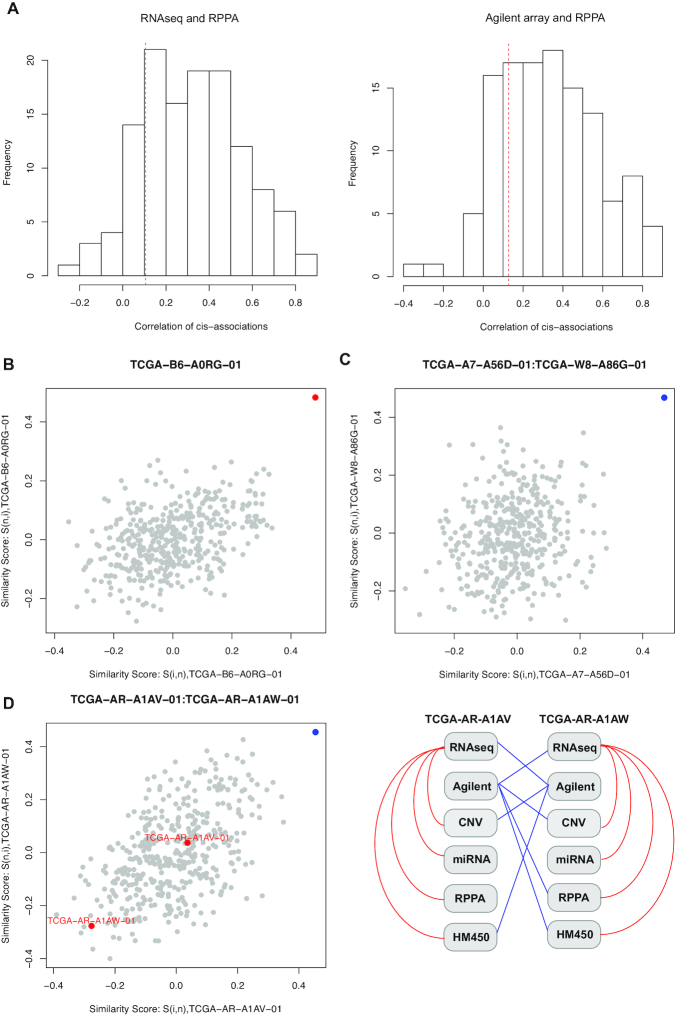
Aligning mRNA and RPPA profiles. **(A)** The Spearman correlations of protein abundance and the corresponding mRNA expression level are shown based on RNAseq and Agilent array. The red line indicates correlation values corresponding to *q*-value 0.05. **(B)** Similarity scores of a self-aligned RNAseq-miRNA profile pair. **(C)** Similarity scores of a cross-aligned RNAseq-miRNA profile pair. **(D)** Similarity scores of the cross-aligned pair between the mRNA Agilent microarray and RPPA profiles, TCGA-AR-A1AV-01 and TCGA-AR-A1AW-01, and alignment results for other omics profiles of this pair into a patient-centric view.

There were 424 tumor samples with both mRNA expression measured in Agilent microarray and RPPA data, and 856 tumor samples with both mRNA expression measured in RNAseq and RPPA data. A total of 145 proteins were mapped to unique mRNA transcripts, and 97 and 104 of the protein-mRNA pairs whose protein abundance was significantly correlated (*q* < 0.05) with the corresponding mRNA's expression level were defined as significant *cis*-associations based on Agilent microarray and RNAseq data, respectively (Fig. 7A and Table 2). And 84.9% and 80.3% of candidate profile pairs were identified as self-aligned by proMODMatcher (Table   2). Examples of similarity scores of a self-aligned RNAseq-miRNA profile pair (Fig. 7B) and a cross-alignment (Fig. 7C, Supplementary Fig. S5) comparing with other possible pairs are shown. The cross-aligned pair of the mRNA Agilent microarray profile TCGA-AR-A1A**V**-01 and the RPPA profile of TCGA-AR-A1A**W**-01 data was identified (Fig. 7D), consistent with labeling errors in the mRNA Agilent array data (Fig. 7D). However, this pair was not identified by MODMatcher (Table 2). The potential cross-alignment between the mRNA Agilent microarray profile TCGA-AR-A1A**W**-01 and the RPPA profile of TCGA-AR-A1A**V**-01 data was not identified (Fig. 7D), suggesting that proMODMatcher's sensitivity is limited when the number of *cis*-associations is ∼100. A large number of non-random missing data in RPPA data (Supplementary Fig. S6) may also contribute to low sensitivity of the method.

**Table 2.** Application of proMODMatcher to mRNA and RPPA profiles of TCGA BRCA data

Data type Type 1	Type 2	No. samples^1^	No. cis pair^2^	No. (%) self-aligned	No. of cross	Cross-aligned pairs Type 1	Self-aligned in RNA-CNV^3^	Cross-aligned pairs Type 2	By MODMatcher^4^
RNAseq	RPPA	856	104/151	687 (80.3)	1	TCGA-A7-A56D-01	Y	TCGA-W8-A86G-01	Y
Agilent	RPPA	424	97/145	360 (84.9)	11	TCGA-BH-A0DS-01	No: TCGA-BH-A0BA-01	TCGA-E2-A1IL-01	Y
						TCGA-E2-A10C-01	Y	TCGA-LL-A5YN-01	Y
						TCGA-E2-A1B0-01	Y	TCGA-D8-A1JK-01	Y
						**TCGA-AR-A1AV-01**	**No: TCGA-AR-A1AW-01**	**TCGA-AR-A1AW-01**	**No**
						TCGA-E2-A1B6-01	No: TCGA-E2-A1B5-01	TCGA-AR-A255-01	No
						TCGA-A8-A07J-01	Y	TCGA-D8-A1JU-01	No
						TCGA-A8-A0AB-01	Y	TCGA-EW-A1J3-01	No
						TCGA-AN-A04C-01	Y	TCGA-E9-A1N9-01	No
						TCGA-E2-A105-01	Y	TCGA-C8-A1HO-01	Y
						TCGA-AN-A0XL-01	Y	TCGA-D8-A1Y2-01	No
						TCGA-AN-A0XV-01	Y	TCGA-GM-A2DM-01	No

The **bold**face indicates cross-alignments supported by other data.

^1^The number of common sample with both Type 1 and Type 2 profiles.

^2^The number of significant *cis*-pairs at *q*-value < 0.05 at final iteration and the number of *cis*-pairs investigated.

^3^Indicates whether the RNA sample of cross-aligned pairs are self-aligned in alignment between RNA profile (Agilent array or RNAseq) and CNV profile. The aligned pairs are also shown if there is a cross-aligned sample.

^4^Indicates that cross-aligned pairs are cross-aligned by MODMatcher.

### Application to TCGA pan-cancer datasets

**Figure 8.**
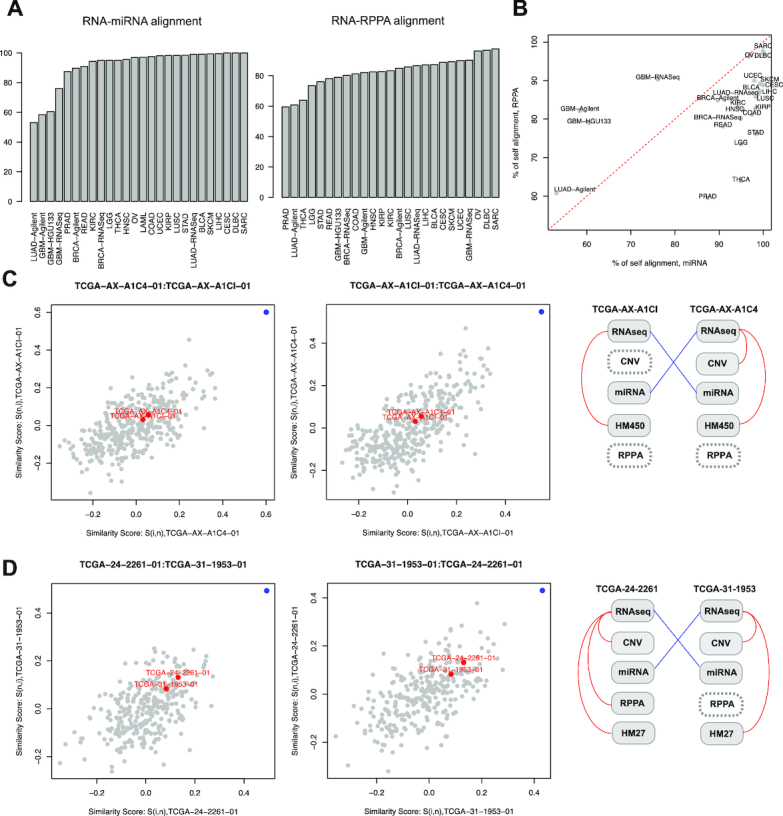
Application to TCGA pan-cancer datasets. **(A, B)** The self-alignment rate of RNA-miRNA and RNA-RPPA alignment for each cancer type. **(C, D)** Two possible sample swap cases of miRNA profiles in the TCGA UCEC and OV datasets. The similarity scores of each cross-alignment and alignment result for other available omics profiles are shown.

The proMODMatcher was also applied to pan-cancer datasets (a total of 22 different types of cancers) in TCGA to align miRNA (Table 3) and RPPA profiles (Table 4) with mRNA profiles. When aligning RNAseq and miRNAseq profiles, >95% of candidate profile pairs were identified as self-aligned for most cancer datasets (Fig. 8A). The self-alignment rates for sarcoma (SARC), lymphoid neoplasm diffuse large B-cell lymphoma (DLBC), and cervical and endocervical cancers (CESC) were 100%, suggesting high data quality for the datasets (Fig. 8A, Table 3). On the other hand, miRNA expression profiles were aligned to mRNA expression profiles (i.e., Agilent, HG-U133, or RNAseq) at a low self-alignment rate for the glioblastoma multiforme (GBM) dataset (Fig. 8A), suggesting low quality of the TCGA GBM miRNA profiles.

**Table 3.** Application of proMODMatcher to mRNA and miRNA profiles of TCGA cancer data excluding BRCA

Type of cancer	Data type Type 1	Type 2	No. common samples	No. cis pair	No. (%) self-aligned	No. (%) cross-aligned	Cross-aligned pairs Type 1	Self in RNA-CNV	Cross-aligned pairs Type 2
BLCA	RNAseq	miRNAseq	405	187/231	402 (99.3)	0			
CESC	RNAseq	miRNAseq	100	132/223	100 (100)	0			
COAD	RNAseq	miRNAseq	248	122/191	242 (97.6)	8 (3.2)	TCGA-CM-4744-01	Y	TCGA-AA-3558-01
							TCGA-QL-A97D-01	Y	TCGA-AA-A00W-01
							TCGA-A6-A567-01	Y	TCGA-AA-3693-01
							TCGA-5M-AATA-01	Y	TCGA-AA-3529-01
							TCGA-RU-A8FL-01	Y	TCGA-AZ-4681-01
							TCGA-QG-A5YV-01	Y	TCGA-AA-A02H-01
							TCGA-A6-A565-01	Y	TCGA-AA-A02E-01
							TCGA-5M-AATE-01	Y	TCGA-AA-A01F-01
DLBC	RNAseq	miRNAseq	47	59/210	47 (100)	0			
GBM	Agilent	miRNA array	525	73/107	307 (58.5)	14 (2.7)	TCGA-02-0064-01	Y	TCGA-08-0390-01
							TCGA-02-0325-01	Y	TCGA-08-0345-01
							TCGA-02-0321-01	Y	TCGA-19-0957-01
							TCGA-08-0510-01	Y	TCGA-26-5135-01
							TCGA-02-0070-01	Y	TCGA-28-5218-01
							TCGA-12-0773-01	Y	TCGA-06-0744-01
							TCGA-12-0780-01	Y	TCGA-08-0354-01
							TCGA-12-0822-01	Y	TCGA-16-1045-01
							TCGA-16-1062-01	Y	TCGA-28-5209-01
							TCGA-14-1829-01	Y	TCGA-14-1450-01
							TCGA-19-1385-01	Y	TCGA-08-0352-01
							TCGA-32-4719-01	Y	TCGA-06-0140-01
							TCGA-19-5952-01	Y	TCGA-02-0324-01
							TCGA-06-0201-01	No	TCGA-06-0141-01
	HG-U133	miRNA array	520	56/100	315 (60.6)	5 (1.0)	TCGA-02-0058-01	No: TCGA-06-0190-01	TCGA-12-0778-01
							TCGA-02-0115-01	Y	TCGA-12-0656-01
							TCGA-19-1789-01	Y	TCGA-06-0413-01
							TCGA-06-2561-01	Y	TCGA-12-0691-01
							TCGA-02-0338-01	Y	TCGA-76-6283-01
	RNAseq	miRNA array	151	70/129	115 (76.2)	19 (12.6)	TCGA-06-1804-01	Y	TCGA-81-5911-01
							TCGA-06-0178-01	No	TCGA-16-1060-01
							TCGA-14-1034-01	Y	TCGA-02-0330-01
							TCGA-15-0742-01	Y	TCGA-02-0116-01
							TCGA-06-5413-01	Y	TCGA-14-0865-01
							TCGA-19-2620-01	Y	TCGA-76-6193-01
							TCGA-06-0158-01	Y	TCGA-06-0174-01
							TCGA-06-0211-01	Y	TCGA-12-3648-01
							TCGA-06-2564-01	Y	TCGA-12-0688-01
							TCGA-06-0141-01	Y	TCGA-08-0246-01
							TCGA-06-0238-01	Y	TCGA-06-0177-01
							TCGA-06-0744-01	Y	TCGA-76-6664-01
							TCGA-06-0125-01	Y	TCGA-08-0358-01
							TCGA-41-2572-01	Y	TCGA-02-0021-01
							TCGA-06-0190-02	Y	TCGA-19-5955-01
							TCGA-28-2499-01	No: TCGA-02-0099-01	TCGA-12-1091-01
							TCGA-06-0152-02	Y	TCGA-26-1799-01
							TCGA-19-1389-02	Y	TCGA-14-0813-01
							TCGA-14-1034-02	Y	TCGA-15-1447-01
HNSC	RNAseq	miRNAseq	517	183/229	494 (95.6)	0			
KIRC	RNAseq	miRNAseq	516	146/205	487 (94.4)	0			
KIRP	RNAseq	miRNAseq	290	131/205	285 (98.3)	0			
LAML	RNAseq	miRNAseq	173	93/166	168 (97.1)	0			
LGG	RNAseq	miRNAseq	526	170/245	500 (95.1)	0			
LIHC	RNAseq	miRNAseq	369	179/228	367 (99.5)	0			
LUAD	RNAseq	miRNAseq	512	179/229	507 (99.0)	0			
	Agilent	miRNAseq	32	32/180	17 (53.1)	3 (9.4)	TCGA-44-2655-01	Y	TCGA-44-6148-01
							TCGA-05-4249-01	No	TCGA-86-A4D0-01
							TCGA-35-4123-01	No	TCGA-55-6969-01
LUSC	RNAseq	miRNAseq	474	191/229	466 (98.3)	0			
OV	RNAseq	miRNAseq	291	159/192	282 (96.9)	5 (1.7)	TCGA-24-2261-01	Y	TCGA-31-1953-01
							TCGA-31-1953-01	Y	TCGA-24-2261-01
							TCGA-61-1728-01	Y	TCGA-23-2072-01
							TCGA-09-0369-01	Y	TCGA-25-1877-01
							TCGA-VG-A8LO-01	Y	TCGA-04-1654-01
PRAD	RNAseq	miRNAseq	494	129/198	432 (87.4)	0			
READ	RNAseq	miRNAseq	66	77/180	60 (90.9)	3 (4.5)	TCGA-AG-A01J-01	Y	TCGA-DY-A1DG-01
							TCGA-AG-A014-01	Y	TCGA-DC-6158-01
							TCGA-AG-A023-01	Y	TCGA-AG-4022-01
SARC	RNAseq	miRNAseq	261	169/220	261 (100)	0			
SKCM	RNAseq	miRNAseq	449	203/251	446 (99.3)	0			
STAD	RNAseq	miRNAseq	377	193/256	371 (98.4)	0			
THCA	RNAseq	miRNAseq	508	139/217	483 (95.1)	0			
UCEC	RNAseq	miRNAseq	361	169/240	354 (98.1)	4 (1.1)	TCGA-A5-A0GP-01	Y	TCGA-AJ-A2QO-01
							TCGA-AX-A1C4-01	Y	TCGA-AX-A1CI-01
							TCGA-AX-A1CI-01	Y	TCGA-AX-A1C4-01
							TCGA-BG-A220-01	No	TCGA-AJ-A3NE-01

Underscore indicates sample swaps.

**Table 4.** Application of proMODMatcher to mRNA and RPPA profiles of TCGA cancer data excluding BRCA

Type of cancer	Data type Type 1	Type 2	No. common samples Type 1	No. *cis* pair	No. (%) self-aligned	No. (%) cross-aligned	Cross-aligned pairs Type 1	Self in RNA-CNV	Cross-aligned pairs Type 2
BLCA	RNAseq	RPPA	340	121/193	297 (87.4)	3 (0.9)	TCGA-XF-AAN8-01	Y	TCGA-FD-A6TB-01
							TCGA-FD-A5BR-01	Y	TCGA-XF-AAMF-01
							TCGA-E7-A6ME-01	Y	TCGA-E7-A541-01
CESC	RNAseq	RPPA	172	101/184	152 (88.4)	1 (0.6)	TCGA-EK-A3GJ-01	Y	TCGA-C5-A8XI-01
COAD	RNAseq	RPPA	240	110/202	195 (81.3)	15 (6.3)	TCGA-G4-6321-01	Y	TCGA-AA-A01P-01
							TCGA-AD-A5EJ-01	Y	TCGA-AA-3672-01
							TCGA-CA-5256-01	Y	TCGA-AA-3815-01
							TCGA-AZ-4682-01	Y	TCGA-G4-6321-01
							TCGA-G4-6303-01	Y	TCGA-A6-2677-01
							TCGA-A6-6137-01	Y	TCGA-AA-A01S-01
							TCGA-G4-6627-01	Y	TCGA-G4-6298-01
							TCGA-A6-6140-01	Y	TCGA-AA-3519-01
							TCGA-NH-A5IV-01	Y	TCGA-AA-A00E-01
							TCGA-G4-6320-01	Y	TCGA-A6-2672-01
							TCGA-DM-A28H-01	Y	TCGA-AA-3811-01
							TCGA-CK-5913-01	Y	TCGA-AA-3664-01
							TCGA-NH-A50U-01	Y	TCGA-AA-3558-01
							TCGA-AD-6901-01	Y	TCGA-NH-A6GC-06
							TCGA-A6-A565-01	Y	TCGA-AA-3520-01
DLBC	RNAseq	RPPA	33	58/184	32 (97.0)	0			
GBM	Agilent	RPPA	191	97/194	157 (82.2)	13 (6.8)	TCGA-06-0139-01	No	TCGA-06-A5U1-01
							TCGA-06-0158-01	Y	TCGA-19-5950-01
							TCGA-06-0176-01	Y	TCGA-19-2625-01
							TCGA-06-0206-01	Y	TCGA-06-0190-02
							TCGA-12-0620-01	Y	TCGA-RR-A6KC-01
							TCGA-06-0881-01	Y	TCGA-02-0003-01
							TCGA-14-1454-01	Y	TCGA-19-A6J5-01
							**TCGA-12**-**1091-01**	**Y**	**TCGA-14**-**1034-02**
							TCGA-14-1037-01	No	TCGA-19-A60I-01
							TCGA-14-1795-01	Y	TCGA-12-5301-01
							TCGA-32-2616-01	Y	TCGA-06-5858-01
							TCGA-81-5911-01	Y	TCGA-19-1389-02
							TCGA-14-1450-01	Y	TCGA-06-5418-01
	HG-U133	RPPA	186	90/187	147 (79.0)	13 (7.0)	TCGA-02-0068-01	Y	TCGA-06-5413-01
							TCGA-02-0033-01	No	TCGA-32-4211-01
							TCGA-14-0781-01	Y	TCGA-74-6575-01
							**TCGA-12**-**1091-01**	**Y**	**TCGA-14**-**1034-02**
							TCGA-28-2509-01	Y	TCGA-19-A60I-01
							TCGA-06-0141-01	Y	TCGA-06-A5U1-01
							TCGA-06-0160-01	Y	TCGA-06-6700-01
							TCGA-06-0394-01	Y	TCGA-74-6578-01
							TCGA-08-0518-01	Y	TCGA-26-6173-01
							TCGA-08-0512-01	Y	TCGA-19-1389-02
							TCGA-02-0330-01	Y	TCGA-06-A6S1-01
							TCGA-32-2491-01	Y	TCGA-06-6698-01
							TCGA-32-4719-01	Y	TCGA-06-0876-01
HNSC	RNAseq	RPPA	212	82/156	175 (82.5)	3 (1.4)	TCGA-CQ-6222-01	No	TCGA-CV-5439-01
							TCGA-D6-6824-01	Y	TCGA-CV-5976-01
							TCGA-MZ-A7D7-01	Y	TCGA-CN-6011-01
KIRC	RNAseq	RPPA	475	125/209	396 (83.4)	4 (0.8)	TCGA-CJ-5681-01	Y	TCGA-B0-5709-01
							TCGA-B0-5709-01	Y	TCGA-CJ-6030-01
							TCGA-CJ-4869-01	Y	TCGA-BP-4771-01
							TCGA-CJ-4888-01	Y	TCGA-CJ-4875-01
KIRP	RNAseq	RPPA	215	93/184	178 (82.8)	3 (1.4)	TCGA-KV-A74V-01	Y	TCGA-MH-A55Z-01
							TCGA-MH-A854-01	Y	TCGA-UZ-A9PL-01
							TCGA-MH-A561-01	Y	TCGA-B1-A47N-01
LGG	RNAseq	RPPA	435	95/173	320 (73.6)	1 (0.2)	TCGA-HT-7681-01	Y	TCGA-P5-A737-01
LIHC	RNAseq	RPPA	181	105/214	158 (87.3)	4 (2.2)	TCGA-ZS-A9CD-01	Y	TCGA-G3-A5SK-01
							TCGA-DD-AAC9-01	Y	TCGA-DD-A4NG-01
							TCGA-G3-AAV0-01	Y	TCGA-GJ-A9DB-01
							TCGA-G3-AAV5-01	Y	TCGA-ED-A627-01
LUAD	RNAseq	RPPA	360	125/193	312 (86.7)	10 (2.8)	TCGA-50-5045-01	No	TCGA-44-7672-01
							TCGA-44-7667-01	Y	TCGA-44-3917-01
							TCGA-MP-A4TI-01	Y	TCGA-MP-A4TA-01
							TCGA-MP-A4TJ-01	Y	TCGA-50-5939-01
							TCGA-50-5055-01	No	TCGA-97-A4M2-01
							TCGA-55-A48X-01	Y	TCGA-64-5778-01
							TCGA-64-5775-01	No	TCGA-05-5715-01
							TCGA-55-6987-01	Y	TCGA-44-2664-01
							TCGA-38-7271-01	Y	TCGA-50-5068-01
							TCGA-55-8208-01	Y	TCGA-50-5066-01
	Agilent	RPPA	23	34/187	14 (60.9)	7 (30.4)	TCGA-44-2661-01	No	TCGA-05-4249-01
							TCGA-05-4249-01	No	TCGA-55-6978-01
							TCGA-44-3398-01	No	TCGA-86-A4JF-01
							TCGA-44-4112-01	No	TCGA-44-3919-01
							TCGA-44-2662-01	Y	TCGA-78-7145-01
							TCGA-67-3774-01	Y	TCGA-73-7498-01
							TCGA-35-3621-01	No	TCGA-44-2661-01
LUSC	RNAseq	RPPA	324	125/193	278 (85.8)	3 (0.9)	TCGA-18-4086-01	Y	TCGA-63-5131-01
							TCGA-39-5039-01	Y	TCGA-34-2604-01
							TCGA-56-A4ZJ-01	Y	TCGA-90-6837-01
OV	RNAseq	RPPA	241	134/202	232 (96.3)	9 (3.7)	TCGA-61-2095-01	Y	TCGA-42-2587-01
							TCGA-09-0364-01	Y	TCGA-29-1774-01
							TCGA-09-2048-01	Y	TCGA-13-0802-01
							TCGA-13-0890-01	Y	TCGA-42-2590-01
							TCGA-24-2035-01	Y	TCGA-30-1892-01
							TCGA-25-1870-01	Y	TCGA-36-2534-01
							TCGA-31-1956-01	Y	TCGA-29-1768-01
							TCGA-57-1583-01	Y	TCGA-61-1916-01
							TCGA-59-2350-01	Y	TCGA-61-1913-01
PRAD	RNAseq	RPPA	351	96/178	209 (59.5)	9 (2.6)	TCGA-VN-A88I-01	Y	TCGA-KC-A4BV-01
							TCGA-KC-A7F3-01	Y	TCGA-ZG-A8QX-01
							TCGA-FC-A6HD-01	No	TCGA-EJ-A8FN-01
							TCGA-EJ-5499-01	Y	TCGA-VN-A88L-01
							TCGA-HC-7230-01	Y	TCGA-HC-7748-01
							TCGA-XJ-A83G-01	Y	TCGA-G9-6338-01
							TCGA-HC-A8CY-01	Y	TCGA-V1-A9Z8-01
							TCGA-HC-7821-01	Y	TCGA-YL-A9WL-01
							TCGA-VP-A87C-01	Y	TCGA-EJ-8470-01
READ	RNAseq	RPPA	55	54/202	43 (78.2)	4 (7.3)	TCGA-AG-A00H-01	Y	TCGA-F5-6810-01
							TCGA-AG-3584-01	Y	TCGA-AG-4022-01
							TCGA-AG-3883-01	Y	TCGA-AG-4005-01
							TCGA-AG-3575-01	Y	TCGA-F5-6863-01
SARC	RNAseq	RPPA	224	110/184	219 (97.8)	0			
SKCM	RNAseq	RPPA	352	128/193	314 (89.2)	2 (0.6)	TCGA-EB-A44N-01	Y	TCGA-EB-A5UM-01
							TCGA-W3-A828-06	Y	TCGA-EB-A551-01
STAD	RNAseq	RPPA	306	103/177	233 (76.1)	12 (3.9)	TCGA-D7-6818-01	Y	TCGA-EQ-8122-01
							TCGA-HU-A4H3-01	Y	TCGA-CG-4442-01
							TCGA-SW-A7EB-01	Y	TCGA-CG-4460-01
							TCGA-VQ-A94P-01	Y	TCGA-RD-A8NB-01
							TCGA-ZA-A8F6-01	Y	TCGA-CG-4476-01
							TCGA-FP-8210-01	Y	TCGA-D7-A4Z0-01
							TCGA-HU-8244-01	Y	TCGA-BR-4371-01
							TCGA-HU-8604-01	Y	TCGA-BR-A4QL-01
							TCGA-HU-A4GJ-01	Y	TCGA-CD-A4MI-01
							TCGA-HU-A4H8-01	Y	TCGA-CG-5720-01
							TCGA-R5-A7ZI-01	Y	TCGA-BR-6710-01
							TCGA-VQ-A927-01	Y	TCGA-F1-A72C-01
THCA	RNAseq	RPPA	222	55/167	142 (64.0)	3 (1.4)	TCGA-EM-A3FJ-01	No	TCGA-EM-A2CS-06
							TCGA-DJ-A4UW-01	No	TCGA-EL-A3CU-01
							TCGA-ET-A3BQ-01	No	TCGA-EL-A3GR-01
UCEC	RNAseq	RPPA	300	115/187	270 (90.0)	15 (5.0)	TCGA-AX-A05Y-01	Y	TCGA-AX-A060-01
							TCGA-AX-A05Z-01	Y	TCGA-EO-A3AV-01
							TCGA-AX-A0IW-01	Y	TCGA-KP-A3VZ-01
							TCGA-D1-A163-01	Y	TCGA-AJ-A3BH-01
							TCGA-D1-A1NZ-01	Y	TCGA-E6-A2P9-01
							TCGA-EO-A22T-01	Y	TCGA-B5-A1MW-01
							TCGA-FI-A2F9-01	Y	TCGA-A5-A1OH-01
							TCGA-BG-A0MQ-01	Y	TCGA-A5-A7WJ-01
							TCGA-BG-A0MO-01	Y	TCGA-BK-A13B-01
							TCGA-D1-A17A-01	Y	TCGA-A5-A0GB-01
							TCGA-BS-A0TE-01	Y	TCGA-AJ-A3EK-01
							TCGA-BS-A0UL-01	Y	TCGA-EO-A22T-01
							TCGA-FI-A2CX-01	Y	TCGA-E6-A2P8-01
							TCGA-B5-A11M-01	No	TCGA-EY-A1GW-01
							TCGA-FI-A2D6-01	Y	TCGA-DF-A2KY-01

For alignments between mRNA and RPPA profiles, the self-alignment rates were lower than alignments between mRNA and miRNA (Fig. 8B) for most datasets due to lower numbers of cis-associations between mRNA and RPPA profiles. The self-alignment rates for DLBC (97.0%) and SARC (97.8%) were higher compared to other datasets (Fig.   8AB), again suggesting high data qualities of the datasets. This observation indicates that some datasets in TCGA showed consistently high confidence for sample quality and low data labeling errors.

Even in datasets of high quality, sample-labeling errors were detected. For example, the self-alignment rate for mRNA-miRNA profiles of the TCGA UCEC dataset was 98.1%. Four cross-alignments were identified (Table 3). Two of them were likely due to a swap of miRNA profiles of TCGA-AX-A1**C4**-01 and TCGA-AX-A1**CI**-01 after considering other types of omics data (Fig. 8C). Similarly, the self-alignment rate for mRNA-miRNA profiles of the TCGA OV dataset was 96.9%. Five cross-alignments were identified (Table 3). Two of them were likely due to a swap of miRNA profiles of TCGA-24-2261-01 and TCGA-31-1953-01 (Fig. 8D).

### Application to ICGC datasets

**Figure 9.**
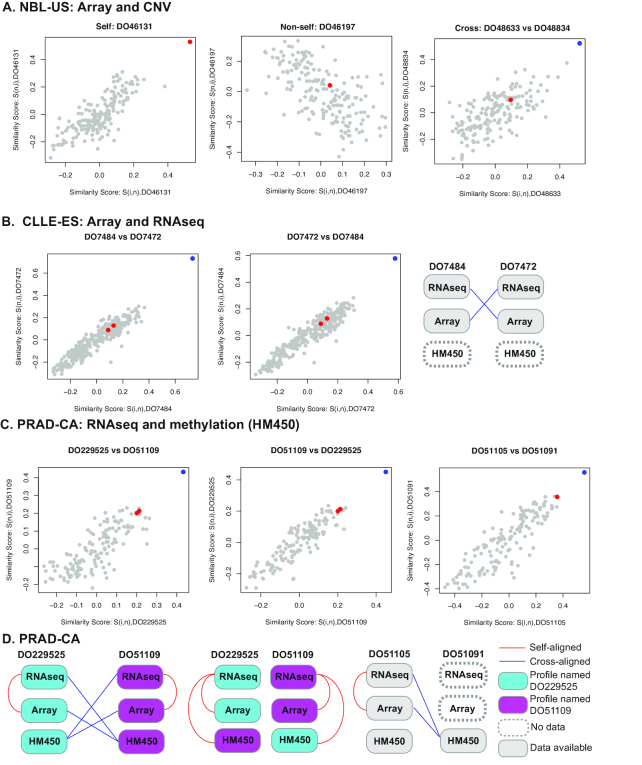
Application to ICGC datasets **(A)** An example of self-self aligned, non–self-self aligned, and cross-aligned pairs of samples based on alignment between Array and CNV profiles in the NBL-US dataset. (**B**) An example of sample-labeling errors. In alignment of Array and DNA methylation profiles, DO7484 and DO7472 were cross-aligned to each other. The similarity scores of each cross-alignment are shown. The similarity score of the cross-aligned pair is shown in blue, and the similarity scores of self-self alignments are shown in red. Omics profiles of DO7484 and DO7472 were compared with each other and results were summarized into a patient-centric view. The red line indicates self-aligned, the and blue line indicates cross-aligned. **(C)** An example of possible sample swaps and sample-labeling errors. DO229525 and DO51109 were cross-aligned to each other in alignment of RNAseq and DNA methylation profiles as well as Array and DNA methylation profiles. Additionally, the RNAseq and Array profiles of DO51105 were cross-aligned to the DNA methylation profile of DO51091. (**D**) Other omics profiles of these pairs were compared with each other and results were summarized into a patient-centric view. After swapping the corresponding DNA methylation profiles, multiple-omics profiles of DO229525 and DO51109 were aligned to each other consistently.

We applied proMODMatcher to 8 cancer datasets that were generated by institutes in the USA, Spain, UK, Germany, Australia, Canada, and France. Each dataset contains >1 types of omics data including mRNA expression profiles (i.e., RNAseq and Array), DNA methylation profiles based on Illumina HM450, miRNA expression profiles, and copy number somatic mutation profiles. The ICGC datasets used and the associated alignment results are summarized in Table 5. In some of the datasets such as PAEN-AU and PRAD-FR, all profiles were matched to other corresponding profiles of the same sample names (Table 5). On the other hand, several sample errors were identified in some datasets. For example, mapping between gene expression Array and CNV profiles in the NBL-US dataset resulted in 170 self-self aligned sample pairs, 10 non–self-self aligned samples, and 12 cross-mapped pairs of profiles (examples shown in Fig. 9A). Mapping gene expression profiles by RNAseq and Array in the CLLE-ES dataset yielded 5 non–self-self aligned samples and 2 cross-mapped pairs of samples. The 2 cross-mapped pairs of samples were likely due to a swap of either RNAseq profile or Array profile (Fig. 9B). Similarly, proMODMatcher identified 3 cross-alignments between RNAseq and DNA methylation profiles in the PRAD-CA dataset, which were also involved in cross-mappings when mapping Array and DNA methylation profiles: 2 of them were likely due to a swap of DNA methylation (HM450) profiles of DO229525 and DO51109 (Fig. 9CD), and 1 of them was likely due to sample-labeling errors in DNA methylation array (HM450) (Fig. 9CD).

**Table 5.** Application of proMODMatcher to datasets with multiple types of omics datasets from the ICGC database

Dataset	Cancer type	Country	Data type Type 1	Type 2	No. samples	No. cis pair	No. self	No. non-self	No. cross
CLLE-ES	Chronic lymphocytic leukemia	Spain	Exp-Array	Methylation	139	3,614	139	0	0
			Exp-Array	Exp-Seq	293	12,753	288	5	2
			Exp-Seq	Methylation	101	3,666	101	0	0
MALY-DE	Malignant lymphoma	Germany	Exp-Seq	miRNA	49	134	49	0	0
PAEN-AU	Pancreatic cancer endocrine neoplasms	Australia	Exp-seq	CNV	32	2,205	32	0	0
			Exp-Array	CNV	23	541	23	0	0
			Exp-Array	Exp-Seq	21	3,425	21	0	0
			Exp-Seq	Methylation	32	3,902	32	0	0
			Exp-Array	Methylation	31	3,845	31	0	0
NBL-US	Neuroblastoma	USA	Exp-Array	CNV	180	2,396	170	10	12
OV-AU	Ovarian	Australia	Exp-Seq	Methylation	80	1,045	80	0	0
			Exp-Seq	miRNA	82	56	79	3	0
PRAD-CA	Prostate cancer adenocarcinoma	Canada	Exp-Array	Exp-Seq	136	10,676	133	3	0
			Exp-Array	Methylation	210	3,114	196	14	4
			Exp-Seq	Methylation	142	4,263	132	10	3
PRAD-FR	Prostate cancer adenocarcinoma	France	Exp-Array	Exp-Seq	25	4249	25	0	0
PACA-AU	Pancreatic cancer	Australia	Exp-Array	Exp-Seq	72	7,548	72	0	0
			Exp-Array	CNV	121	1,041	118	3	0
			Exp-Seq	CNV	79	1,327	78	1	0
			Exp-Seq	Methylation	77	5,538	77	0	0
			Exp-Array	Methylation	174	2,514	169	5	1

## Discussion

We developed a sample alignment method, proMODMatcher, for detecting and correcting sample-labeling errors by aligning omics profiles. The proMODMatcher extended our previous method MODMatcher by estimating probabilities of potential matches rather than using ranks of similarity scores. Applied to simulated datasets, proMODMatcher outperformed MODMatcher when aligning the omics data profiles with a relatively small number of *cis*-associations. We showed that the number of candidate intrinsic *cis*-associations between mRNA-miRNA profiles or mRNA-RPPA profiles was low. Application of our proMODMatcher to alignment between mRNA-miRNA profile pairings and mRNA-RPPA profile pairings from 22 different cancer datasets in TCGA demonstrated that sample-labeling errors occurred even in datasets of high quality and our procedure was not only able to identify sample-labeling errors but also to unambiguously identify the source of the errors.

**Figure 10.**
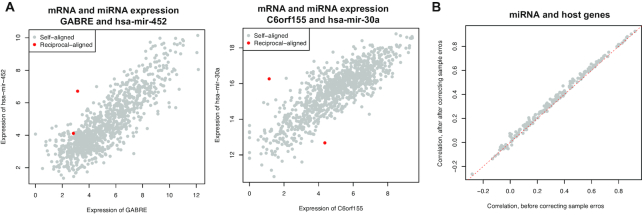
Correcting sample-labeling errors. **(A)** Mis-labeled samples were outliers when comparing significant pairs between mRNA and miRNA expression levels in the TCGA BRCA dataset. Red dots were mis-labeled samples. **(B)** Spearman correlation between expression levels of miRNAs and their host genes before and after curating sample-labeling errors.

Integrating multi-omics data into comprehensive network models is essential to elucidate complex molecular mechanisms of cancers. After correcting sample-labeling errors, associations between different profiles were stronger. For example, mis-labeled samples were outliers when comparing significant pairs between mRNA and miRNA expression levels in the TCGA BRCA dataset (Fig. 10A, red dots were mis-labeled samples). Spearman correlations between expression levels of miRNAs and their host genes were improved for most pairs of miRNA-host genes after curating sample-labeling errors (Fig. 10B).

We showed that some potential cross-aligned profile pairs in the TCGA BRCA dataset were missed by proMODMatcher. The sensitivity and accuracy of multi-omics profile-matching methods need further improvement. Integrating >2 types of profiles in probability estimation may yield more robust sensitivity and specificity when the number of *cis*-associations is small.

The proMODMatcher depends on a set of biological *cis*-associations, and the information content (Shannon entropy) of each *cis*-association depends on the randomness of the genotype at each locus or gene expression of each gene. For example, if there were 2 possible genotypes at a locus, then randomness or Shannon entropy is maximized when the probability of each genotype is 50%. When the probabilities of the 2 genotypes deviate from equal, the randomness or Shannon entropy at the locus decreases. Thus, in our analyses, we excluded biological *cis*-associations that are driven by extreme values (rare events). For example, in eQTL analyses, we only included loci of minor allele frequency >0.05. Missing values commonly occur in high-throughput omics data. In our analyses, we do not explicitly impute missing values. Instead, we filtered out probes or genes of >25% missing value in the data pre-processing step.

The computational cost of applying proMODMatcher is small. For example, mapping mRNA and miRNA expression profiles for 408 samples took 802 seconds of CPU time with maximum memory usage of 503 MB on a machine with CPU processor of 3.50 GHz.

## Potential implications

Our results demonstrated that sample-labeling errors were common in large multi-omics datasets. Our method has improved statistical accuracy to identify and curate these errors over the previous method and is generally applicable to other datasets. Application of our general framework for automated curation of public databases and properly merging omics data would be the fundamental basis for the development of effective integrative approaches.

## Methods

### A general framework of multi-omics data matching: Pairwise alignments based on *cis*-associations

We followed the general framework of multi-omics data matching of the previous study [8]. Two types of data (or profiles) (i.e., Type A and Type B in Fig. 1) were matched on the basis of their *cis*-associations. Probes in different types of data were matched by intrinsic biological relationships. For example, probes in methylation, miRNA, and CNV profiles were mapped to a close transcript based on hg19 reference genome. Samples were initially matched on the basis of annotated sample ID and potential *cis*-associations (Fig. 1A). The significant *cis*-associations from 2 different data types were identified by the Spearman correlations at Benjamini-Hochberg (BH) adjusted *q*-value < 0.05 (Fig. 1B). The data for each *cis*-association was normal rank-transformed as RT(*A_n,i_*) and RT(*B_n,i_*), where *A_n,i_* and *B_n,i_* represent the measurements of sample *i* and *n*th *cis-*related probes for Type A and B profiles, respectively (Fig. 1B). For simplicity, we omitted all normal rank transformation in the rest of the notations. The profile similarity between the 2 types of data $S( {{A_i},\ {B_j}} )$ is defined as (Fig. 1C):

\begin{eqnarray*}
&& S\ \left( {{A_i},{B_j}} \right) = \mathrm{corr} \left( {{A_i},\ {B_j}} \right)\nonumber\\
&&\quad\quad = \frac{{\mathop \sum \nolimits_{n = 1}^N {A_{n,i}}\mathop \sum \nolimits_{n = 1}^N {B_{n,j}} - N\mathop \sum \nolimits_{n = 1}^N {A_{n,i}} \times {B_{n,j}}}}{{\sqrt {N\mathop \sum \nolimits_{n = 1}^N {A_{n,i}}^2 - {{\left( {\mathop \sum \nolimits_{n = 1}^N {A_{n,i}}} \right)}^2}} \sqrt {N\mathop \sum \nolimits_{n = 1}^N {B_{n,j}}^2 - {{\left( {\mathop \sum \nolimits_{n = 1}^N {B_{n,j}}} \right)}^2}} }}.\
\end{eqnarray*}

First, profile pairs matched by annotated sample IDs were checked to determine whether their similarity scores were high (Fig. 1D), in which case they were annotated as “self-aligned.” If not, additional steps were applied to find any potential matches among other unmatched profiles (Fig. 1E). The matched profile pairs were then used to update significant *cis*-associations. We iteratively refined profile alignment, and rounds of alignments were repeated until there were no further updates.

### Biological *cis*-associations

“Biological *cis*-associations” reflect different biological regulations when different pairs of omics data are mapped. (1) *cis*-eQTLs for mapping genotype and gene expression data: a genetic polymorphism at a gene's promoter or regulatory region affects binding of transcription factors or co-factors, which in turn affects the abundance of the gene's transcripts [11]. If the genetic polymorphism occurs within 1 million bases from the gene's transcription start site and the association is significant at FDR <0.05, the association is called a *cis*-eQTL. (2) *cis*-methylations for mapping DNA methylation and gene expression data: increased DNA methylation at CpG sites near a gene promoter region is associated with gene repression [12]. A methylation probe is assigned to the transcript whose start site is closest to the genomic location of the methylation probe when it is potentially mapped to multiple transcripts. If a DNA methylation probe locates within 1 million bases from the gene's start site and the association between the methylation level and the gene's expression level is significant at FDR <0.05, the methylation probe is a *cis*-methylation probe. (3) *cis*-CNVs for mapping DNA CNVs and gene expression profiles: amplified or deleted genomic regions can regulate the expression levels of genes within that genomic region [19]. If a gene's expression is associated with its CNV at FDR <0.05, the CNV is a *cis*-CNV. (4) *cis*-miRNA-gene pairs for mapping miRNA and gene expression profiles: a small portion of miRNAs are embedded in gene regions (i.e., host genes) and frequently co-transcribed with host genes [17, 18]. If the expression levels of an miRNA and its host gene are associated at FDR <0.05, the pair is a *cis*-miRNA-gene pair. (5) *cis*-mRNA-protein pairs for mapping protein and gene expression profiles: the abundance of a protein depends on the corresponding mRNA transcript level and other factors [20]. If their association is significant at FDR <0.05, the pair is a *cis*-mRNA-protein pair.

### Multi-Omics Data matcher (MODMatcher)

In the “determine self-aligned vs cross-aligned” step (Fig. 1E), the similarity scores of self-aligned profiles between Type A and Type B, $S( {{A_i},\ {B_i}} )$, were top 5% ranked among $S( {{A_n},\ {B_i}} ),\ n\ = \ 1 \ldots {N_A}$ as well as $S( {{A_i},\ {B_n}} ),\ n\ = \ 1 \ldots {N_B}$, to be annotated as *self-aligned*, where ${N_A}\ $ and ${N_B}$ represent the number of samples of Type A and Type B, respectively. If the sample sizes were >400, top 20 was used as the threshold for self-alignment. Next, for the profiles that were not self-aligned, reciprocal mapping was applied to find any potential matches among other unmatched profiles. If the similarity score of sample *j* of Type A and sample *k* of Type B, $S( {{A_i},\ {B_k}} )\ $, is first ranked among $S( {{A_j},\ {B_n}} ),\ n\ = \ 1 \ldots {N_B}$ as well as $S( {{A_n},\ {B_k}} ),\ n\ = \ 1 \ldots {N_A}$, then the pair is annotated as *cross-aligned*.

### A probabilistic Multi-Omics Data matcher (proMODMatcher)

The characteristics (e.g., noises, biases, dynamic ranges) of 2 types of profiles may be different. The rank-based cut-off was not able to reflect similarity score differences in a specific similarity score distribution with a large or small variance (Supplementary Fig. S7). In the “determine self- vs cross-aligned” step, the proMODMatcher evaluated a similarity score in a bivariate normal distribution, ${\rm{{\rm X}}}\sim\,{N_2}( {{{\bf \mu }},\ {{\bf \Sigma }}} )$, where ${{\bf \mu }}$ is the mean vector and ${{\bf \Sigma }}$ is the covariance matrix (Fig. 1D). The probability of a match between profile *i* of Type A and profile *j* of Type B, ${\rm{P}}\ ( {{A_i},\ {B_j}} ) = \ P( {S( {{A_i},\ {B_j}} ),\ S( {{A_i},\ {B_j}} )} )$, is estimated on the basis of a score distribution of $\ ( {S( {{A_i},\ {B_m}} ),\ S( {{A_m},\ {B_j}} )} )$, where *A_m_* and *B_m_* represent the Type A and Type B profile of the *m*th matched profile pairs, respectively. Given the bivariate normal distribution, we calculated the distance of a point $x\ = \ ( {S( {{A_i},\ {B_m}} ),\ S( {{A_m},\ {B_j}} )} )$ to the center of the distribution, known as the Mahalanobis distance, as $\ r\ = \left[{{( {x - {{\bf \mu }}} )}^T}{{\rm{\Sigma }}^{ - 1}}( {x - {{\bf \mu }}} )\right]^{1/2} \ $, and the cumulative function $F\ ( {R \le r} ) = {\rm{\ }}1 - {e^{ - {r^2}/2}}$. To obtain a more robust estimation of the covariance matrix ${{\bf \Sigma }}$ of the distribution, we added 1,000 profile pairs of randomly permuted profiles in addition to true profile pairs.

Additionally, we introduced a prior probability of self-alignment ${p_0}$. Thus, given profiles *A_i_* and *B_j_* and their similarity score $S( {{A_i},\ {B_j}} )\ $ as well as the estimated Mahalanobis distance *r_i_,_j_*, we calculated the *p*-value of the 2 profiles matched by chance as

\begin{eqnarray*}
p( {{A_i},\ {B_j}} ){\rm{\ }} = \left \lbrace {\begin{array}{@{}*{1}{c}@{}} {{p_0}*{e^{ - r_{i,\ j}^2/2}},\ {\rm{if}}\ i\ = \ j}\\ {{e^{ - r_{i,\ j}^2/2}},\ {\rm{if}}\ i \ne j} \end{array}}\right. \ \!.
\end{eqnarray*}

In this study, the prior probability ${p_0}$ was set as ${p_0} = 1/{N_s}$, where ${N_s}$ represents the number of samples. We also set global similarity score cut-offs for self-alignment, $S_{\mathrm{self}}^{\mathrm{cut-off}}$, as well as cross-alignment, $S_{\mathrm{cross}}^{\mathrm{cut-off}}$. The $S_{\mathrm{self}}^{\mathrm{cut-off}}$ value was set as the lower bound of 99% of the self-self similarity scores estimated by mean and standard deviations of $S( {{A_i},\ {B_i}} )$, where *i* indicates the samples with both Type A and Type B profiles. And the $S_{\mathrm{cross}}^{\mathrm{cut-off}}\ $ was set as the lower bound of 68% of the self-self similarity scores.

The similarity score $S( {{A_i},\ {B_j}} )$ and its corresponding *p*-value $p( {{A_i},\ {B_j}} )$ were used to identify matched pairs between Type A and Type B profiles (Fig. 1E). Each round of our procedure consisted of 3 steps. First, the self-alignment similarity score $S( {{A_i},\ {B_i}} )$ and corresponding *p*-value $p( {{A_i},\ {B_i}} )$ were calculated. If $S( {{A_i},\ {B_i}} )\ $ > $S_{\mathrm{self}}^{\mathrm{cut-off}}$ and $( {{A_i},\ {B_i}} ) < {p_{i \ne j}}( {{A_i},\ {B_j}} )$, then the profiles ${A_i}$ and ${B_i}$ were self-aligned. Second, for a profile ${A_i}$ that was not self-aligned to the profile ${B_i}$ in the first step, it was compared to all unmapped profile ${B_j}$. If the similarity score $S( {{A_i},\ {B_j}} ) < S_{\mathrm{cross}}^{\mathrm{cut-off}}\ $ and the corresponding *p*-value $\ p( {{A_i},\ {B_j}} ) \le arg\mathop {\min }\limits_{n \in [ {1 \ldots ,\ {N_B}} ]} ( {p( {{A_i},\ {B_n}} )} )$and $\ p( {{A_i},\ {B_j}} ) \le arg\mathop {\min }\limits_{n \in [ {1 \ldots ,\ {N_A}} ]} ( {p( {{A_n},\ {B_j}} )} )$, then the profiles ${A_i}$ and ${B_j}$ were cross-aligned. Third, for profile pairs ${A_i}$ and ${B_i}$ that were not aligned in the first 2 steps, if $S( {{A_i},\ {B_i}} )\ $ > $S_{\mathrm{self}}^{\mathrm{cut-off}}$ and the *p*-value $p( {{A_i},\ {B_i}} )$ was smaller than the fifth smallest among $p( {{A_i},\ {B_n}} ),\ n\ = \ 1 \ldots {N_B}$ as well as $p( {{A_n},\ {B_i}} ),\ n\ = \ 1 \ldots {N_A}$, then the profiles ${A_i}$ and ${B_i}$ were rescued as self-aligned. The rounds of alignments were repeated until there was no further change.

### Correlation of *cis*-associated mRNA and miRNA before and after correction of labeling errors

To assess improvement of signals after labeling error correction, we calculated the Spearman correlation between miRNA expression and its host genes with initially matched pairs based on sample ID and with aligned sample pairs. To avoid bias due to different number of samples, we matched the number of samples of initially matched pairs to the number of aligned pairs. We randomly selected the samples with the same number of aligned pairs, and calculated the Spearman correlation. We performed random selection 100 times and calculated the mean of correlation.

## Availability of source code and requirements

Project name: ProMODMatcher (passcode to decrypt the zipped file is “password123”)

Project home page: Github site (https://github.com/integrativenetworkbiology/proMODMatcher) and http://labs.icahn.mssm.edu/zhulab/software/

Operating system: Platform independent

Programming language: R (R 3.5.1 or later)

Other requirements: R package mnormt

License: GNU General Public License

RRID: SCR_01 7219

## Availability of supporting data and materials

Data supporting the results of this article are publicly available at the Firehose database, TCGA data portal, and ICGC data portal (see Data Description). Data further supporting this work and snapshots of our code are available in the *GigaScience* repository, GigaDB [21].

## Additional files

**Supplementary Figure S1**. Similarity scores of matched pairs based on simulated datasets.

**Supplementary Figure S2**. Similarity scores of cross-aligned pairs detected by only MODMatcher based on aligning RNASeq and miRNA profiles of BRCA data.

**Supplementary Figure S3**. Similarity scores of cross-aligned pairs based on aligning RNA and miRNA profiles of BRCA data. (A) Alignment of RNAseq and miRNAseq profiles. (B) Alignment of Agilent array and miRNAseq profiles.

**Supplementary Figure S4**. Similarity scores of cross-aligned pairs detected by only MODMatcher based on aligning Array and miRNA profiles of BRCA data.

**Supplementary Figure S5**. Similarity scores of cross-aligned pairs based on aligning RNA and RPPA profiles of BRCA data. (A) Aligning of RNAseq and RPPA profiles. (B) Aligning of Agilent array and RPPA profiles.

**Supplementary Figure S6**. The frequency of genes of which the number of samples with non-assigned values are greater than each percent of samples.

**Supplementary Figure S7**. Examples of distribution of similarity scores. The red asterisk indicates the values of the highest similarity score.

**Supplementary Table S1**. Sample alignment results by MODMatcher for mRNA (Array and RNAseq) -miRNAseq profiles based on BRCA data.

**Supplementary Table S2**. Sample alignment results by MODMatcher for mRNA (Array and RNAseq) -RPPA profiles based on BRCA data.

giz080_GIGA-D-19-00039_Original_Submission

giz080_GIGA-D-19-00039_Revision_1

giz080_GIGA-D-19-00039_Revision_2

giz080_Response_to_Reviewer_Comments_Original_Submission

giz080_Response_to_Reviewer_Comments_Revision_1

giz080_Reviewer_1_Report_Original_Submission Ettore Mosca -- 3/13/2019 Reviewed

giz080_Reviewer_1_Report_Revision_1 Ettore Mosca -- 5/31/2019 Reviewed

giz080_Reviewer_2_Report_Original_Submission Sijia Huang -- 4/16/2019 Reviewed

giz080_Reviewer_2_Report_Revision_1 Sijia Huang -- 5/31/2019 Reviewed

giz080_Supplemental_File

## Abbreviations

BH: Benjamini-Hochberg; BLCA: bladder urothelial carcinoma; BRCA: breast invasive carcinoma; CESC: cervical and endocervical cancers; CNV: copy number variation; CPU: central processing unit; eCOAD: colon adenocarcinoma; DLBC: lymphoid neoplasm diffuse large B-cell lymphoma; eQTL: expression quantitative trait locus; FPR: false-positive rate; GBM: glioblastoma multiforme; HM27: Illumina HumanMethylation27 Beadchip; HM450: Illumina HumanMethylation450 Beadchip; HNSC: head and neck squamous cell carcinoma; ICGC: International Cancer Genome Consortium; KIRC: kidney renal clear cell carcinoma; KIRP: kidney renal papillary cell carcinoma; LGG: brain lower grade glioma; LIHC: liver hepatocellular carcinoma; LUAD: lung adenocarcinoma; LUSC: lung squamous cell carcinoma; miRNA: microRNA; MODMatcher: Multi-Omics Data matcher; mRNA: messenger RNA; OV: ovarian serous cystadenocarcinoma; PRAD: prostate adenocarcinoma; proMODMatcher: probabilistic Multi-Omics Data matcher; READ: rectum adenocarcinoma; RNAseq: RNA sequencing; RPPA: reverse phase protein array; SARC: sarcoma; SKCM: skin cutaneous melanoma; SNP: single-nucleotide polymorphism; STAD: stomach adenocarcinoma; TCGA: The Cancer Genome Atlas; THCA: thyroid carcinoma; UCEC: uterine corpus endometrial carcinoma.

## Competing interests

The authors declare that they have no competing interests.

## Funding

This work was partially supported by the National Institutes of Health (grants R01-AG046170, U01-HG008451, and U19-AI118610).

## Authors’ contributions

E.L. and J.Z. designed research. E.L. performed research and analyzed data. S.Y. contributed to download data and analyzed data by MODMatcher method. W.W. contributed design of simulation. Z.T. contributed revising paper. E.L. and J.Z. wrote the paper. All authors read and approved the final manuscript.
