# Supplementary material for: A probabilistic multi-omics data matching method for detecting sample errors in integrative analysis
Source: Gigascience. 2019 Jul 9;8(7):giz080. doi: 10.1093/gigascience/giz080 (PMC6615984; doi:10.1093/gigascience/giz080)
Supplement: giz080_GIGA-D-19-00039_Revision_2 [file giz080_giga-d-19-00039_revision_2.pdf]

GigaScience

A probabilistic multi-omics data matching method for detecting sample errors in integrative analysis

--Manuscript Draft--

**Manuscript Number:**	GIGA-D-19-00039R2
**Full Title:**	A probabilistic multi-omics data matching method for detecting sample errors in integrative analysis
**Article Type:**	Research
**Funding Information:**	National Institutes of Health (R01-AG046170) — Dr. Jun Zhu<br>National Institutes of Health (U01-HG008451) — Dr. Jun Zhu<br>National Institute of Health (U19 AI118610) — Dr. Jun Zhu
**Abstract:**	Background: Data errors, including sample swapping and mis-labeling are inevitable in the process of large-scale omics data generation. Data errors need to be identified and corrected before integrative data analyses where different types of data are merged based on the annotated labels. Data with labeling errors dampen true biological signals. More importantly, data analysis with sample errors could lead to wrong scientific conclusions. We developed a robust probabilistic multi-omics data matching procedure, proMODMatcher, to curate data, identify and correct data annotation and errors in large databases.<br>Results: Application to simulated datasets suggests that proMODMatcher achieved robust statistical power even when the number of cis-associations was small and/or the number of samples was large. Application of our proMODMatcher to multi-omics datasets in The Cancer Genome Atlas (TCGA) and International Cancer Genome Consortium (ICGC) identified sample errors in multiple cancer datasets. Our procedure was not only able to identify sample labeling errors but also to unambiguously identify the source of the errors. Our results demonstrate that these errors should be identified and corrected before integrative analysis.<br>Conclusions: Our results indicate that sample labeling errors were common in large multi-omics datasets. These errors should be corrected before integrative analysis.
**Corresponding Author:**	Jun Zhu<br>Icahn School of Medicine at Mount Sinai<br>UNITED STATES
**Corresponding Author Secondary Information:**	
**Corresponding Author's Institution:**	Icahn School of Medicine at Mount Sinai
**Corresponding Author's Secondary Institution:**	
**First Author:**	Eunjee Lee
**First Author Secondary Information:**	
**Order of Authors:**	Eunjee Lee<br>Seungyeul Yoo<br>Wenhui Wang<br>Zhidong Tu<br>Jun Zhu
**Order of Authors Secondary Information:**	
**Response to Reviewers:**	Reviewer reports:<br>Reviewer #1: The authors responded appropriately to my comments. The manuscript still requires some editing for language and clarity, such as:

Powered by Editorial Manager® and ProduXion Manager® from Aries Systems Corporation

	- 417. "The sensitivity and accuracy of multi-omics profile matching 418 methods needs further improvement" should be "The sensitivity and accuracy […] need further improvement".<br>We thank the reviewer for pointing this out. We corrected the grammar error.<br><br>- 421. "The proMODMatcher depends on a set of biological cis-associations and the information content (Shannon entropy) of each cis-association depends on the randomness of each locus or gene".<br>Here, the "randomness" attributed to "each locus or gene" is unclear and requires further explanation.<br>As the reviewer suggested, we modified the sentence as the following :<br>"The proMODMatcher depends on a set of biological cis-associations and the information content (Shannon entropy) of each cis-association depends on the randomness of genotypes at each locus or expression of each gene. For example, if there were two possible genotypes at a locus, then randomness or Shannon entropy is maximized when the probability of each genotype is 50%. If the probabilities of the two genotypes deviate from equal, the randomness or Shannon entropy at the locus decreases."<br><br>Reviewer #2: Most of the issues have been addressed.<br><br>One question regarding the package is regarding the resource of these mapping files, where are they coming from? Are they up-to-date? Are they all experiment validated?<br>For Methylation data, we downloaded annotation file for HM27 and HM450 Illumina BeadChip. For miRNA, based on the coordinates of genes and miRNA, we mapped miRNA-host genes. For protein, we mapped the protein whose gene symbol is same as the mRNA id. All mapping files are based on most updated coordinates in chromosome of genes and probes.. There is no experiment attempted to validate beyond associations.<br><br>It will be much better if you can provide the links for these files and offer an automatic way of updating, with standardized IDs for each category (gene expression, methylation, CNV, proteins etc.)<br>We thank the reviewer's suggestion. We modified the code and readme file to take standardized IDs and use the mapped files if a user prefers.
**Additional Information:**	
**Question**	**Response**
Are you submitting this manuscript to a special series or article collection?	No
**Experimental design and statistics**<br><br>Full details of the experimental design and statistical methods used should be given in the Methods section, as detailed in our Minimum Standards Reporting Checklist. Information essential to interpreting the data presented should be made available in the figure legends.<br><br>Have you included all the information requested in your manuscript?	Yes

Powered by Editorial Manager® and ProduXion Manager® from Aries Systems Corporation

**Resources**<br><br>A description of all resources used, including antibodies, cell lines, animals and software tools, with enough information to allow them to be uniquely identified, should be included in the Methods section. Authors are strongly encouraged to cite Research Resource Identifiers (RRIDs) for antibodies, model organisms and tools, where possible.<br><br>Have you included the information requested as detailed in our Minimum Standards Reporting Checklist?	Yes
**Availability of data and materials**<br><br>All datasets and code on which the conclusions of the paper rely must be either included in your submission or deposited in publicly available repositories (where available and ethically appropriate), referencing such data using a unique identifier in the references and in the “Availability of Data and Materials” section of your manuscript.<br><br>Have you have met the above requirement as detailed in our Minimum Standards Reporting Checklist?	Yes

Powered by Editorial Manager® and ProduXion Manager® from Aries Systems Corporation

Manuscript with DOI
Click here to view linked References
Click here to access/download;Manuscript;GigaScience_pSA_v4.4_revision2

A probabilistic multi-omics data matching method for detecting sample errors in integrative analysis

Eunjee Lee[1,2,3], Seungyeul Yoo[1,2], Wenhui Wang[1,2], Zhidong Tu[1,2] and Jun Zhu[1,2,3,4,*]

[1]Department of Genetics and Genomic Sciences; [2]Icahn Institute of Genomics and Multiscale Biology, Icahn School of Medicine at Mount Sinai, New York, NY 10029; [3]Sema4, a Mount Sinai venture, Stamford, CT, USA; [4]The Tisch Cancer Institute, Icahn School of Medicine at Mount Sinai, New York, NY 10029;

*All corresponds should be addressed to Dr. Jun Zhu (jun.zhu@mssm.edu)

Eunjee Lee (eunjee.lee@mssm.edu)
Seungyeul Yoo (seungyeul.yoo@mssm.edu)
Wenhui Wang (wenhui.wang@mssm.edu)
Zhidong Tu (zhidong.tu@mssm.edu)
Jun Zhu (jun.zhu@mssm.edu)

Abstract

Background: Data errors, including sample swapping and mis-labeling are inevitable in the process of large-scale omics data generation. Data errors need to be identified and corrected before integrative data analyses where different types of data are merged based on the annotated labels. Data with labeling errors dampen true biological signals. More importantly, data analysis with sample errors could lead to wrong scientific conclusions. We developed a robust *probabilistic* multi-omics data matching procedure, *pro*MODMatcher, to curate data, identify and correct data annotation and errors in large databases.

Results: Application to simulated datasets suggests that *pro*MODMatcher achieved robust statistical power even when the number of cis-associations was small and/or the number of samples was large. Application of our *pro*MODMatcher to multi-omics datasets in The Cancer Genome Atlas (TCGA) and International Cancer Genome Consortium (ICGC) identified sample errors in multiple cancer datasets. Our procedure was not only able to identify sample labeling errors but also to unambiguously identify the source of the errors. Our results demonstrate that these errors should be identified and corrected before integrative analysis.

Conclusions: Our results indicate that sample labeling errors were common in large multi-omics datasets. These errors should be corrected before integrative analysis.

Keywords: data error, omics data integration, and data curation

Background

With advances in high throughput technologies in the past two decades, diverse types of omics data at multiple layers of regulation have been generated to survey complex human diseases [1-3], which arise from dysregulations of interplays among these multiple layers of regulations including genetics, epigenetics, transcriptomics, metabolomics, glycomics, and proteomics. Therefore, integration of multi-omics data at multiple layers of regulation is essential to derive a holistic view of molecular mechanisms underlying complex human disease. Previous studies have shown that simultaneously considering diverse types of biological data result in more complete understandings of biological systems [4-6].

Recently, many large projects, such as The Cancer Genome Atlas (TCGA) and International Cancer Genome Consortium (ICGC), have generated diverse types of omics data for public use. However, data errors, including sample swapping, mis-labeling, and improper data entry are almost inevitable in the process of large-scale data generation and management. Westra *et al*. [7] showed that there is about 20% of mis-matched samples between genotype and gene expression data. Yoo *et al.* [8] demonstrated that sample labeling errors occurred in almost every database examined. Also, there are studies to identify cross-individual contamination in next-generation sequencing data from TCGA samples [9, 10].

Identifying and ultimately correcting these sample errors are critical for statistical data analysis, especially for integrative analysis. Data errors need to be identified and corrected before extensive efforts being devoted to data analysis. Analyzing data with sample errors is a waste of limited public resources. More importantly, data analysis with sample errors could lead to wrong scientific conclusions. Furthermore, sample errors have more significant effect on integrative data analysis where different types of data are merged based on the annotated labels. Some types of sample errors can be detected during data quality control (QC) on each

individual type of data, whereas sample errors including sample swapping, or mis-labeling are elusive to be detected by data QC on individual type of data alone.

Previously, we developed sample mapping procedure called MODMatcher (Multi-Omics Data matcher) [8], which is not only able to identify mis-matched omics profile pairs, but also to properly map them to correct samples based on other omics data. The main idea is first to identify "biological *cis*-associations" between two types of omics data, and then to use these "biological *cis*-associations" as intrinsic barcodes to match different types of omics data. The types of "biological *cis*-associations" are different when different pairs of omics data are mapped, but they all reflect general biological regulations. For example, when mapping genotype and gene expression data, the method is based on *cis*-genetic regulation of expression traits (or expression quantitative trait loci—*cis*-eQTLs), where a genetic polymorphism at a gene's promotor or regulatory region affects transcription factors or co-factors binding, which in turn affects the abundance of the gene's transcript [11]. Similarly, when mapping methylation and gene expression data, the method leverages on *cis*-methylation regulation of expression traits (or *cis*-methyls), where high DNA methylation level of CpGs at a gene's promotor or regulatory region hinders transcription factors or co-factors binding, which in turn represses the gene's transcription [12]. More on "biological *cis*-associations" are detailed in the Methods section.

We demonstrated that the statistical power to identify biological signals increases after database cleaning by applying the MODMatcher procedure to multiple large-scale public multi-omics datasets from LGRC and TCGA. The power of MODMatcher depends on the number of intrinsic biological cis-associations that can be identified. The power of MODMatcher decreases when the number of cis-associations between two omics profiles is small. However, in some cases (a few examples are detailed in the Results), the number of possible intrinsic biological cis-associations is small, new methods are needed for these types of applications.

In this study, we extended MODMatcher and developed a robust *probabilistic* multi-omics data matching procedure, *pro*MODMatcher, to curate data, identify and unambiguously correct data annotation and metadata attribute errors in large databases. First, we applied the *pro*MODMatcher to simulated datasets to assess the statistical power of our procedure. Results suggest that *pro*MODMatcher achieved robust statistical power even when the number of cis-associations was small and/or the number of samples was large. Next, we applied the *pro*MODMatcher procedure to multiple large-scale publicly available multi-omics datasets from TCGA, and in particular, focused on the omics profiles that have small numbers of intrinsic *cis*-associations including miRNA expression and Reverse Phase Protein Array (RPPA). Additionally, we applied *pro*MODMatcher to large-scale publicly available multi-omics datasets in ICGC. Our results indicate that sample labeling errors were common in large multi-omics datasets. These errors should be corrected before integrative analysis.

Data Description

TCGA datasets

For the TCGA breast invasive carcinoma (BRCA) dataset, level 3 data of gene expression, DNA methylation, miRNA expression and CNV was downloaded from Genomic Data Commons (GDC) data portal (https://portal.gdc.cancer.gov/). For gene expression profiles, IlluminaHiSeq_RNASeqV2 and AgilentG4502A platform were used. Illumina HumanMethylation27 (HM27) and HumanMethylation450 (HM450) Beadchip were used for DNA methylation bisulfide sequencing. IlluminaHiSeq_miRNASeq and IlluminaGA_miRNASeq platforms were used to profile miRNA expression. Affymetrix Genome-Wide Human SNP Array 6.0 was used for copy number variation. The protein expression levels were measured in Reverse Phase Protein Array (RPPA), and downloaded. Each of level 3 profiles was

reformatted for matrix of row with gene (or probes) and column with barcodes of samples. For methylation profiles and CNV, the probes or segments were mapped to hg19 gene symbols. Different profiles were initially matched according to their barcodes. The mapping files of HM450, RPPA, and miRNA are available in the source code.

For other types of cancers in TCGA, we downloaded gene expression, miRNA expression, CNV, DNA methylation, and RPPA data from firehose database https://gdac.broadinstitute.org/. For RPPA data, we filtered genes with more than 25% of samples with not-assigned measurements.

ICGC datasets

For the ICGC datasets, the pre-processed data were downloaded from ICGC data portal (https://dcc.icgc.org/). We selected datasets with more than one available types of omics data including mRNA expression profiles (i.e. RNAseq and Array), DNA methylation profiles based on Illumina HumanMethylation450 (HM450), miRNA expression profiles, and copy number somatic mutation profiles. Each of profiles was reformatted into a matrix with genes (or probes) as rows and barcodes of samples as columns. The gene and miRNA expression profiles were log2 transformed and normalized by quantile normalization[13]. For copy number somatic mutation profiles, the segments were mapped to hg19 gene symbols. Some datasets contain very sparse segment information for copy number somatic mutation profiles such as CLLE-ES. We excluded these copy number profiles for further analysis. For methylation profiles, the probes were mapped to hg19 gene symbols.

Simulation study

Simulated data sets for testing alignment between a pair of omics profiles were generated.

Given a set of N *cis*-associations and each of correlation coefficient r_n, we can simulate omics profiles Υ based on omics profiles X for M samples as following: $X_i = N(0,1)$ is a standard normal distribution, and $\gamma_i = \frac{r_n}{\sqrt{1-r_n^2}} X_i + \epsilon$, where ϵ is standard normal distribution, $N(0,1)$. For each N and M combination, we simulated N significant sets with r_n drawn from a truncated normal distribution with a cutoff value corresponding to correlation coefficients q-value < 0.05, as well as 2000 sets of random r_n drawn from a normal distribution. We considered N significant *cis*-associations from 75 through 1000, and M samples from 100 through 1000. The simulated data with label error were generated by permuting the labels of one type of data. We considered 0, 2, .. 10% label error rates. We measured sensitivity (i.e. recall) $=\frac{\#truly\ aligned\ pairs}{\#simulated\ pairs}$, specificity (i.e. precision) $= \frac{\#truely\ aligned\ pairs}{\#align\ pairs}$, false positive rate (FPR)=1-specificity, and F measures ($= 2 \times \frac{precision \times recall}{precision + recall}$) for assessment. Additionally, because a pair of omics profiles mostly has unbalanced samples, we mimics this by adding 10% of M samples for type A and type B omics profiles.

Analyses

<u>Overview of *pro*MODMatcher procedure</u>

*pro*MODMatcher followed the general framework of multi-omics data matching of the previous study [8]. Two types of data (or profiles) (i.e. Type A and Type B in **Figure 1**) were matched based on their *cis*-associations. Samples were initially matched based on annotated sample ID and potential *cis*-associations (**Figure 1A**). The significant *cis*-associations from two different data types were identified by the Spearman correlations (**Figure 1B**). The data for each *cis*-association was normal rank-transformed (**Figure 1B)**. The profile similarity between the two

types of data $S(A_i, B_j)$ is defined as the correlation between profile *i* of type A and profile *j* of type B (**Figure 1C**). The probability of a match between profile *i* of type A and profile *j* of type B is estimated by evaluating a similarity score in a bivariate normal distribution (**Figure 1D**). Based on probability of a match, *pro*MODMatcher determines self- or cross-alignments for each match. First, profile pairs matched by annotated sample IDs were checked whether their similarity scores were high (**Figure 1D**) to be annotated as "self-aligned". If not, additional steps were applied to find any potential matches among other unmatched profiles (**Figure 1E**). The matched profile pairs were then used to update significant *cis*-associations. We iteratively refined profile alignment and rounds of alignments were repeated until there were no further updates (**Figure 1F**).

Simulation studies

Numbers of significant *cis*-associations and samples are two important deterministic factors of similarity scores as well as the accuracy of omics profile alignment results. To investigate the effect of numbers of samples and *cis*-associations, we simulated data sets with different numbers of samples and significant *cis*-associations and applied MODMatcher and *pro*MODMatcher to the simulated data sets. For MODMatcher, when the number of cis-associations was >200, almost all profile pairs could be aligned at high accuracy (false positive rate vs. sensitivity) (**Figure 2**). The similarity scores of matched pairs based on a low number of *cis*-associations were more variable resulting in lower accuracies (**Supplementary Figure S1**). This result indicates that the MODMatcher can be applied to align the omics profile pairs with >200 *cis*-associations, such as methylation-mRNA profiles with over 7000 intrinsic *cis*-associations and mRNA-CNV profiles with over 10,000 intrinsic *cis*-associations [8]. On the other hand, when the number of *cis*-associations was around 200 or below, the accuracy of

sample alignments dropped as the number of samples increased (**Figure 2**). When aligning gene expression profiles with miRNA or RPPA profiles, the number of candidate intrinsic cis-associations was small (detailed below). Thus, MODMatcher was not powered to accurately align these types of profile pairs.

The *pro*MODMatcher was applied to the same simulated datasets and was able to achieve high sensitivities and low FPRs across a wide range of numbers of *cis*-associations and samples (**Figure 3A**). When compared with MODMatcher's results, *pro*MODMatcher resulted in better accuracies (F measure in **Figure 3B**), similar sensitivities (**Figure 3C**), and better specificities (**Figure 3D**).

We further investigated their performances when there were labeling errors. Datasets with sample labeling errors (i.e. 4% and 6%) were simulated by randomly assigning some samples' labels, then *pro*MODMatcher and MODMatcher were applied to identify aligned profile pairs**.** As expected, when a larger number of *cis*-associations was available, *pro*MODMatcher achieved a high sensitivity and low FPR (**Figure 3A**). Across all tested combinations of numbers of *cis*-associations and samples, *pro*MODMatcher resulted in >99% accuracy with 4-6% input labeling error rates, consistently outperformed MODMatcher (**Figure 3B**). The top goal of MODMatcher and *pro*MODMatcher is to identify sample labeling errors without introducing any errors. Thus, we optimized the specificity of *pro*MODMatcher over its sensitivity. In terms of sensitivity and specificity's contribution to F scores, *pro*MODMatcher achieved a similar sensitivity as MODMatcher (**Figure 3C**) but better specificities in all cases (**Figure 3D**). These simulation results suggest that *pro*MODMatcher is applicable for identifying and correcting labeling errors even when the number of *cis*-associations is small such as paring mRNA-miRNA or mRNA-RPPA profiles.

Application to TCGA breast cancer dataset: mRNA and miRNA profiles

Multiple omics data, including profiles of mRNA, miRNA, protein, DNA methylation, and CNV, were available in TCGA. The *pro*MODMatcher was applied to align methylation and/or CNV profiles to mRNA profiles similar to what we did previously [8]. Here we focused on alignment of miRNA expression profiles to mRNA expression data because the number of candidate intrinsic cis-associations between miRNA and mRNA profiles was small. We used the TCGA breast cancer (BRCA) dataset as an example to illustrate the profile alignment results in detail. There were mRNA expression profiles based on two different platforms, Agilent microarray and RNAseq technology. There were 519 tumor samples with both mRNA expression measured in Agilent microarray and miRNA expression measured by small-RNA sequencing method, and 1041 tumor samples with both mRNA expression measured in RNAseq and miRNA measured by small-RNA sequencing method. A small portion of miRNAs are embedded in gene regions (i.e. host genes) and frequently co-transcribed with host genes [14, 15] (**Figure 4A**), embedded miRNA-host gene pairs were candidate intrinsic *cis*-associations. Total 1222 miRNAs were profiled, and 227 and 271 of them were mapped to host genes, for Agilent microarray and RNAseq data, respectively. Among them, 138 out of 227 and 175 out of 271 miRNA-host genes pairs were significantly associated with each other at q-value<0.05, for Agilent microarray and RNAseq data, respectively. For example, miR-452 located in the gene body of *GABRE*, its expression was highly associated with mRNA expression of *GABRE* (**Figure 4B**). Based on these intrinsic *cis*-associations between expression levels of miRNAs and host genes, we aligned the two types of omics data.

Aligning gene expression profiles by RNAseq and miRNAseq data

The similarity scores of self-aligned gene expression-miRNA expression profiles were much higher than other possible pairings in general (**Figure 4C**): 898 out of 1041 (86.2%) the similarity scores for self-self RNAseq-miRNAseq profiles were ranked at top 2%. For example, the similarity score for the self-aligned profiles of TCGA−D8−A1JH-01 was top ranked among other possible pairings (**Figure 4D**). Total 143 miRNA profiles that were not matched to the corresponding mRNA profiles of the same sample names based on MODMatcher (e.g. TCGA−B6−A0X7-01 shown in **Figure 4E**). Among profile pairs that were not self-aligned, 5 for RNAseq profiles were cross-aligned to other samples' miRNA profiles (**Supplementary Table S1**). The rate of alignment was low compared to alignments of other types of profile pairs. For example, >99% profile pairs of DNA methylation and mRNA expression profiles were aligned for the TCGA BRCA data set.

Table 1. Application of *pro*MODMatcher to mRNA and miRNA profiles of TCGA BRCA data.

Data types	Data types	# samples[1]	# cis pair [2]	# of self-aligned	# of cross	Cross-aligned pairs	Self-aligned in RNA-CNV[3]	Cross-aligned pairs	By MODMatcher[4]
Type1	Type 2					Type 1		Type 2	
RNAseq	miRNAseq	1041	175/215	989 (95.0%)	1	**TCGA-BH-A0BZ-01**	**Y**	**TCGA-E2-A15K-01**	**Y**
Agilent	miRNAseq	519	138/178	466 (89.7%)	9	TCGA-A8-A07U-01	Y	TCGA-A2-A3XY-01	Y
						TCGA-BH-A0H9-01	Y	TCGA-EW-A423-01	N
						TCGA-AO-A128-01	Y	TCGA-BH-A18V-06	Y
						**TCGA-A1-A0SD-01**	**No: TCGA-BH-A0EI-01**	**TCGA-BH-A0EI-01**	**Y**
						TCGA-BH-A18K-01	No: TCGA-BH-A18T-01	TCGA-BH-A18T-01	Y
						TCGA-BH-A18T-01	No: TCGA-BH-A18K-01	TCGA-BH-A18K-01	Y
						**TCGA-BH-A0BZ-01**	**Y**	**TCGA-E2-A15K-01**	**Y**
						**TCGA-BH-A0BS-01**	**No: TCGA-BH-A0BT-01**	**TCGA-BH-A0BT-01**	**Y**
						TCGA-AR-A0U0-01	Y	TCGA-AR-A256-01	Y

The **bold** indicates cross-alignments supported by other data and underlines indicates sample swaps.
[1]The number of common sample with both type1 and type2 profiles.
[2]The number of significant cis-pairs at q-value <0.05 at final iteration and the number of cis-pairs investigated.
[3]Indicating the RNA samples of cross-aligned pairs were self-aligned or not in alignment between RNA profile (Agilent array or RNAseq) and CNV profile. The aligned pairs were also shown if there was a cross-aligned sample.
[4]Indicating whether the cross-aligned pairs were cross-aligned by MODMatcher.

Applying *pro*MODMatcher to TCGA BRCA RNAseq-miRNAseq datasets, the

probabilities of similarity scores (before multiplying prior probability) for self-aligned RNAseq-miRNA profiles were much higher than other possible pairs in general (**Figure 4F**). An example of similarity scores of a self-aligned RNAseq-miRNA profile pair and other possible pairs is shown in **Figure 4G**. There were multiple self-self pairs with low probabilities for self-alignment (**Figure 4F** and **Figure 4H**), suggesting potential labeling errors in RNAseq and/or miRNA profiles. Overall, 989 out of 1041 candidate matching pairs (i.e. 95.0%) (**Table 1**) were self-aligned compared to 86.2% for MODMatcher. Among profiles that were not self-aligned, 1 profile pair (i.e. TCGA-BH-A0BZ-01 and TCGA-E2-A15K-01) was cross-aligned to each other (**Table 1**).

Comparing MODMatcher and *pro*MODMatcher, the *pro*MODMatcher identified additional 91 self-aligned profile pairs that were missed by MODMatcher. For example, the similarity score of self-alignment for TCGA-AO-A0JF-01 was among the highest one when the miRNA profile compared to RNAseq profiles of other samples (y-axis in **Figure 5A**). However, the RNAseq profile of TCGA-AO-A0JF-01 was highly similar with multiple miRNA profiles of other samples (x-axis in **Figure 5A**). As a result, the rank-based MODMatcher rejected the self-alignment, but *pro*MODMatcher identified self-alignment for TCGA-AO-A0JF-01 with p-value of 7.3×10^{-6}.

One cross-aligned pair, RNAseq of TCGA-BH-A0BZ-01 and miRNA of TCGA-E2-A15K-01, was identified by both *pro*MODMatcher and MODMatcher. The similarity score of the cross-aligned pair is shown in **Figure 5B**. The similarity scores of self-self alignments were low (red dots in **Figure 5B**); on the other hand, the similarity score of the cross-aligned pair was significantly higher compared to other similarity scores (**Figure 5B)**, indicating high confidence of cross-alignment. On the other hand, the cross-aligned pairs detected only by MODMatcher showed relatively marginal similarity scores even though the similarity scores of cross-aligned pairs were the highest (**Supplementary Figure S2**). Furthermore, we compared significance

levels of *cis*-associations based on profile pairs aligned by MODMatcher and *pro*MODMatcher. They were comparable in general with a few highly significant *cis*-associations more significant based on *pro*MODMatcher compared to MODMatcher (**Figure 5C**).

Aligning gene expression profiles by Agilent microarray and miRNAseq data

MODMatcher and *pro*MODMatcher were also applied to align mRNA expression profiles based Agilent microarray and miRNA profiles. There were 138 *cis*-associations identified based on Agilent microarray data and miRNAseq data. Based on these cis-associations, 87% of candidate profile pairs were identified as self-aligned by MODMatcher (**Supplementary Table S1**) while 89.7% of candidate profile pairs were self-aligned by *pro*MODMatcher (**Table 1**).

Among profiles that were not self-aligned, 9 cross-aligned profile pairs were identified by *pro*MODMatcher (**Table 1, Supplementary Figure S3B**), 8 out of 9 pairs were also detected by MODMatcher (**Table 1**). MODMatcher detected additional cross-aligned pairs including several questionable cross-aligned pairs (i.e. TCGA−E2−A153−01 and TCGA−E9−A1NG−01, TCGA-AR-A1AL−01 and TCGA−AR−A1AN−01 in **Supplementary Figure S4**). The cross-aligned pairs by *pro*MODMatcher included a possible swap between TCGA-BH-A18**K**-01 and TCGA-BH-A18**T**-01 (**Figure 6A** and **Table 1**). To determine the source of labeling errors (due to mRNA Agilent profiles or miRNA profiles) other omics profiles were compared with each other and results were summarized into a patient-centric view (**Figure 6B**). For patient/sample TCGA-BH-A18**K,** the RNAseq and miRNAseq profiles were self-aligned and the RNAseq and CNV profiles were self-aligned as well (**Figure 6B**). Similarly, for patient/sample TCGA-BH-A18**T,** the RNAseq profile was self-aligned to the miRNA, CNV, and DNA methylation profiles as well as the RPPA profile (detailed below) (**Figure 6B**). The cross-alignments of TCGA-BH-A18K-01 and TCGA-BH-A18T-01 mRNA Agilent profiles with their miRNA profiles (**Figure 6B**) indicate

sample swapping occurred in mRNA Agilent array profiles. After swapping the corresponding mRNA Agilent array profiles, multiple-omics profiles of TCGA-BH-A18K and TCGA-BH-A18T were aligned to each other consistently (**Figure 6C**). Our previous study based on pairwise profile alignments of gene expression, DNA methylation and CNV also identified the sample swaps in mRNA Agilent array profiles of TCGA-BH-A18K-01 and TCGA-BH-A18T-01 [8] (**Figure 6B-C**). In addition, *pro*MODMatch identified a cross-alignment of the mRNA Agilent array profile of TCGA-A1-A0SD-01 and the miRNA profile of TCGA-BH-A0EI-01 (**Table 1**, **Figure 6D**), consistent with potential sample swaps of mRNA Agilent array profiles of TCGA-A1-A0SD-01 and TCGA-BH-A0EI-01 when alignments of other omics profiles were included. Similarly, the cross-alignment between the Agilent array profile of TCGA-BH-A0B**<u>S</u>**-01 and the miRNA profile of TCGA-BH-A0B**<u>T</u>**-01 was likely a result of a swap between the Agilent array profiles of the two samples when adding all available omics data into the comparison (**Figure 6E**).

The *pro*MODMatcher identified a cross-aligned pair between the mRNA Agilent array profile of TCGA-BH-A0BZ-01 and the miRNA profile of TCGA-E2-A15K-01(See **Table 1, Figure 6F**). The miRNA profile of TCGA-E2-A15K-01 was also cross-aligned to the mRNAseq profile of TCGA-BH-A0BZ-01 (**Table 1, Figure 5B**). When including alignments of other omics profiles in a patient-centric view (**Figure 6F**), the result suggests that there was a labeling error of the miRNA profile of TCGA-E2-A15K-01.

These results together suggest that *pro*MODMatcher with 138 *cis*-associations can accurately identify sample labeling errors and unambiguously correct labeling errors.

<u>Application to TCGA breast cancer dataset: mRNA and RPPA profiles</u>

There were 424 tumor samples with both mRNA expression measured in Agilent microarray and RPPA data, and 856 tumor samples with both mRNA expression measured in RNAseq and RPPA data. Total 145 proteins were mapped to unique mRNA transcripts, and 97 and 104 of protein-mRNA pairs whose protein abundance was significantly correlated ($q<0.05$) with the corresponding mRNA's expression level were defined as significant *cis*-associations based on Agilent microarray and RNAseq data, respectively (**Figure 7A** and **Table 2**). And 84.9% and 80.2% of candidate profile pairs were identified as self-aligned by *pro*MODMatcher (**Table 2**). Examples of similarity scores of a self-aligned RNAseq-miRNA profile pair (**Figure 7B**) and a cross-alignment (**Figure 7C, Supplementary Figure S5**) comparing with other possible pairs are shown. The cross-aligned pair of the mRNA Agilent microarray profile TCGA-AR-A1A**V**-01 and the RPPA profile of TCGA-AR-A1A**W**-01 data was identified (**Figure 7D**), consistent with labeling errors in the mRNA Agilent array data (**Figure 7D**). However, this pair was not identified by MODMatcher (**Table 2**). The potential cross-alignment between the mRNA Agilent microarray profile TCGA-AR-A1A**W**-01 and the RPPA profile of TCGA-AR-A1A**V**-01 data was not identified (**Figure 7D)**, suggesting *pro*MODMatcher's sensitivity is limited when the number of *cis*-associations is around 100. A large number of non-random missing data in RPPA data (**Supplementary Figure S6**) may also contribute to low sensitivity of the method.

Table 2. Application of *pro*MODMatcher to mRNA and RPPA profiles of TCGA BRCA data

Data types	Data types	# samples[1]	# cis pair [2]	# of self-aligned	# of cross	Cross-aligned pairs	Self-aligned in RNA-CNV[3]	Cross-aligned pairs	By MODMatcher[4]
Type1	Type 2					Type 1		Type 2	
RNAseq	RPPA	856	104/151	687 (80.2%)	1	TCGA-A7-A56D-01	Y	TCGA-W8-A86G-01	Y
Agilent	RPPA	424	97/145	360 (84.9%)	11	TCGA-BH-A0DS-01	No :TCGA-BH-A0BA-01	TCGA-E2-A1IL-01	Y
						TCGA-E2-A10C-01	Y	TCGA-LL-A5YN-01	Y
						TCGA-E2-A1B0-01	Y	TCGA-D8-A1JK-01	Y
						**TCGA-AR-A1AV-01**	**No: TCGA-AR-A1AW-01**	**TCGA-AR-A1AW-01**	**N**
						TCGA-E2-A1B6-01	No:TCGA-E2-A1B5-01	TCGA-AR-A255-01	N
						TCGA-A8-	Y	TCGA-D8-	N

						A07J-01		A1JU-01	
						TCGA-A8-A0AB-01	Y	TCGA-EW-A1J3-01	N
						TCGA-AN-A04C-01	Y	TCGA-E9-A1N9-01	N
						TCGA-E2-A105-01	Y	TCGA-C8-A1HO-01	Y
						TCGA-AN-A0XL-01	Y	TCGA-D8-A1Y2-01	N
						TCGA-AN-A0XV-01	Y	TCGA-GM-A2DM-01	N

The **bold** indicates cross-alignments supported by other data.
[1]The number of common sample with both type1 and type2 profiles.
[2]The number of significant cis-pairs at q-value <0.05 at final iteration and the number of cis-pairs investigated.
[3]Indicate the RNA sample of cross-aligned pairs are self-aligned or not in alignment between RNA profile (Agilent array or RNAseq) and CNV profile. The aligned pairs are also shown if there is a cross-aligned sample.
[4]Indicate cross-aligned pairs are cross-aligned by MODMatcher.

Application to TCGA pan-cancer datasets

The *pro*MODMatcher was also applied to pan-cancer datasets (total 22 different types of cancers) in TCGA to align miRNA (**Table 3**) and RPPA profiles (**Table 4**) with mRNA profiles. When aligning RNAseq and miRNAseq profiles, more than 95% of candidate profile pairs were identified as self-aligned for most cancer datasets (**Figure 8A**). The self-alignment rates for SARC, DLBC, and CESC were 100%, suggesting high data quality for the datasets (**Figure 8A, Table 3**). On the other hand, miRNA expression profiles were aligned to mRNA expression profiles (i.e. Agilent, HG-U133, or RNAseq) at low self-alignments rate for the GBM dataset (**Figure 8A**), suggesting low quality of the TCGA GBM miRNA profiles.

For alignments between mRNA and RPPA profiles, the self-alignment rates were lower than alignments between mRNA and miRNA (**Figure 8B**) for most datasets due to lower numbers of cis-associations between mRNA and RPPA profiles. The self-alignment rates for DLBC (96.97%) and SARC (97.7%) were higher compared to other datasets (**Figure 8AB**), again suggesting high data qualities of the datasets. This observation indicates some datasets in TCGA showed consistently high confidence for sample quality and low data labeling errors.

Even in datasets of high quality, sample labeling errors were detected. For example, the self-alignment rate for mRNA-miRNA profiles of the TCGA UCEC dataset was 98%. Four

cross-alignments were identified (**Table 3**). Two of them were likely due to a swap of miRNA profiles of TCGA-AX-A1**<u>C4</u>**-01 and TCGA-AX-A1**<u>CI</u>**-01 after considering other types of omics data (**Figure 8C**). Similarly, the self-alignment rate for mRNA-miRNA profiles of the TCGA OV dataset was 96.9%. Five cross-alignments were identified (**Table 3**). Two of them were likely due to a swap of miRNA profiles of TCGA-24-2261-01 and TCGA-31-1953-01 (**Figure 8D**).

<u>Application to ICGC datasets</u>

We applied *pro*MODMatcher to 8 cancer datasets that were generated by institutes in the U.S., Spain, UK, Germany, Australia, Canada, and France. Each dataset contains more than one types of omics data including mRNA expression profiles (i.e. RNAseq and Array), DNA methylation profiles based on Illumina HumanMethylation450 (HM450), miRNA expression profiles, and copy number somatic mutation profiles. The ICGC datasets used and the associated alignment results were summarized in **Table 5**. In some of datasets such as PAEN-AU and PRAD-FR, all profiles were matched to other corresponding profiles of the same sample names (**Table 5)**. On the other hand, several sample errors were identified in some datasets. For example, mapping between gene expression Array and CNV profiles in the NBL-US dataset resulted in 170 self-self aligned sample pairs, 10 non self-self aligned samples and 12 cross-mapped pairs of profiles (examples shown in **Figure 9A**). Mapping gene expression profiles by RNAseq and Array in the CLLE-ES dataset yielded five non self-self aligned samples and two cross-mapped pairs of samples. The two cross-mapped pairs of samples were likely due to a swap of either RNAseq profile or Array profile (**Figure 9B**). Similarly, *pro*MODMatcher identified three cross-alignments between RNAseq and DNA methylation profiles in the PRAD-CA dataset, which were also involved in cross-mappings when mapping Array and DNA methylation profiles: two of them were likely due to a swap of DNA

methylation (HM450) profiles of DO229525 and DO51109 (**Figure 9CD**), and one of them was likely due to sample labeling errors in DNA methylation array (HM450) (**Figure 9CD**).

Table 5. Application of *pro*MODMatcher to datasets with multiple types of omics datasets from ICGC database

Dataset	Cancer type	Country	Data types	Data types	# samples	# cis pair	# self	# non-self	# cross
			Type1	Type 2					
CLLE-ES	Chronic Lymphocytic Leukemia	Spain	Exp-Array	Methylation	139	3614	139	0	0
			Exp-Array	Exp-Seq	293	12753	288	5	2
			Exp-Seq	Methylation	101	3666	101	0	0
MALY-DE	Malignant Lymphoma	Germany	Exp-Seq	miRNA	49	134	49	0	0
PAEN-AU	Pancreatic Cancer Endocrine neoplasms	Australia	Exp-seq	CNV	32	2205	32	0	0
			Exp-Array	CNV	23	541	23	0	0
			Exp-Array	Exp-Seq	21	3425	21	0	0
			Exp-Seq	Methylation	32	3902	32	0	0
			Exp-Array	Methylation	31	3845	31	0	0
NBL-US	Neuroblastoma	USA	Exp-Array	CNV	180	2396	170	10	12
OV-AU	Ovarian	Australia	Exp-Seq	Methylation	80	1045	80	0	0
			Exp-Seq	miRNA	82	56	79	3	0
PRAD-CA	Prostate Cance r Adenocarcino ma	Canada	Exp-Array	Exp-Seq	136	10676	133	3	0
			Exp-Array	Methylation	210	3114	196	14	4
			Exp-Seq	Methylation	142	4263	132	10	3
PRAD-FR	Prostate Cance r Adenocarcino ma	France	Exp-Array	Exp-Seq	25	4249	25	0	0
PACA-AU	Pancreatic Cancer	Australia	Exp-Array	Exp-Seq	72	7548	72	0	0
			Exp-Array	CNV	121	1041	118	3	0
			Exp-Seq	CNV	79	1327	78	1	0
			Exp-Seq	Methylation	77	5538	77	0	0
			Exp-Array	Methylation	174	2514	169	5	1

Discussion

We developed a sample alignment method, *pro*MODMatcher, for detecting and correcting sample labeling errors by aligning omics profiles. The *pro*MODMatcher extended our previous method MODMatcher by estimating probabilities of potential matches rather than using ranks of similarity scores. Applied to simulated datasets, *pro*MODMatcher outperformed MODMatcher when aligning the omics data profiles with relatively small number of *cis*-associations. We showed that the number of candidate intrinsic cis-association between mRNA-miRNA profiles or mRNA-RPPA profiles was low. Application of our *pro*MODMatcher to alignment between mRNA-miRNA profile pairings and mRNA-RPPA profile pairings from 22 different cancer

datasets in TCGA demonstrated that sample labeling errors occurred even in datasets of high quality and our procedure was not only able to identify sample labeling errors but also to unambiguously identify the source of the errors.

Integrating multi-omics data into comprehensive network models is essential to elucidate complex molecular mechanisms of cancers. After correcting sample labeling errors, associations between different profiles were stronger. For example, mis-labeled samples were outliers when comparing significant pairs between mRNA and miRNA expression levels in the TCGA BRCA dataset (**Figure 10A,** red dots were mis-labeled samples). Spearman correlation between expression levels of miRNAs and their host genes were improved for most pairs of miRNA-host genes after curating sample labeling errors (**Figure 10B**).

We showed that some potential cross-aligned profiles pairs in the TCGA BRCA dataset were missed by *pro*MODMatcher. The sensitivity and accuracy of multi-omics profile matching methods need further improvement. Integrating more than two types of profiles in probability estimation may yield more robust sensitivity and specificity when the number of cis-associations is small.

The *pro*MODMatcher depends on a set of biological *cis*-associations and the information content (Shannon entropy) of each *cis*-association depends on the randomness of genotype at each locus or gene expression of each gene. For example, if there were two possible genotypes at a locus, then randomness or Shannon entropy is maximized when the probability of each genotype is 50%. When the probabilities of the two genotypes deviate from equal, the randomness or Shannon entropy at the locus decreases. Thus, in our analyses, we excluded biological *cis*-associations that are driven by extreme values (rare events). For example, in eQTL analyses, we only included loci of minor allele frequency (MAF)>0.05. Missing values commonly occur in high throughput omics data. In our analyses, we don't explicitly impute

missing values. Instead, we filtered out probes or genes of more than 25% missing value in the data pre-processing step.

The computational cost of applying *pro*MODMatcher is small. For example, mapping mRNA and miRNA expression profiles for 408 samples took 802 seconds of CPU time with maximum memory usage of 503 MB on a machine with CPU processor 3.50 GHz.

Potential implications

Our results demonstrated that sample labeling errors were common in large multi-omics datasets. Our method has improved statistical accuracy to identify and curate these errors over the previous method, and generally applicable to other data sets. Application of our general framework for automated curation of public databases and properly merging omics data would be the fundamental basis for the development of effective integrative approaches.

Methods

A general framework of multi-omics data matching: Pairwise alignments based on *cis*-associations

We followed the general framework of multi-omics data matching of the previous study [8]. Two types of data (or profiles) (i.e. Type A and Type B in **Figure 1**) were matched based on their *cis*-associations. Probes in different types of data were matched by intrinsic biological relationships. For example, probes in methylation, miRNA and Copy number variation (CNV) profiles were mapped to a close transcript based on hg19 reference genome. Samples were initially matched based on annotated sample ID and potential *cis*-associations (**Figure 1A**). The significant *cis*-associations from two different data types were identified by the Spearman correlations at Benjamini-Hochberg (BH) adjusted q-value < 0.05 (**Figure 1B**). The data for each *cis*-

association was normal rank-transformed as $RT(A_{n,i})$ and $(B_{n,i})$, where $A_{n,i}$ and $B_{n,i}$ represents the measurements of sample *i* and *n*th *cis*-related probes for Type A and B profiles, respectively (**Figure 1B**). For simplicity, we omitted all normal rank transformation in the rest of notations. The profile similarity between the two types of data $S(A_i, B_j)$ is defined as (**Figure 1C**):

$$S(A_i, B_j) = corr(A_i, B_j)$$

$$= \frac{\sum_{n=1}^{N} A_{n,i} \sum_{n=1}^{N} B_{n,j} - N \sum_{n=1}^{N} A_{n,i} \times B_{n,j}}{\sqrt{N \sum_{n=1}^{N} {A_{n,i}}^2 - (\sum_{n=1}^{N} A_{n,i})^2} \sqrt{N \sum_{n=1}^{N} {B_{n,i}}^2 - (\sum_{n=1}^{N} B_{n,i})^2}}$$

First, profile pairs matched by annotated sample IDs were checked whether their similarity scores were high (**Figure 1D**) to be annotated as "self-aligned". If not, additional steps were applied to find any potential matches among other unmatched profiles (**Figure 1E**). The matched profile pairs were then used to update significant *cis*-associations. We iteratively refined profile alignment and rounds of alignments were repeated until there were no further updates.

Biological *cis*-associations

"Biological *cis*-associations" reflect different biological regulations when different pairs of omics data are mapped. (1) *cis*-eQTLs for mapping genotype and gene expression data: a genetic polymorphism at a gene's promotor or regulatory region affects transcription factors or co-factors binding, which in turn affects the abundance of the gene's transcripts [11]. If the genetic polymorphism occurs within 1M bases from the gene's transcription start site and the association is significant at the false discovery rate (FDR) <0.05, the association is called as a *cis*-eQTL. (2) *cis*-methylations for mapping DNA methylation and gene expression data:

increased DNA methylation at CpGs sites near a gene promoter region is associated with gene repression [12]. A methylation probe is assigned to the transcript whose start site is closest to the genomic location of the methylation probe when it is potentially mapped to multiple transcripts. If a DNA methylation probe locates within 1M bases from the gene's start site and the association between the methylation level and the gene's expression level is significant at FDR <0.05, the methylation probe is a *cis*-methylation probe. (3) *cis*-CNVs for mapping DNA copy number variations (CNVs) and gene expression profiles: amplified or deleted genomic regions can regulate the expression levels of genes within that genomic region [16]. If a gene's expression is associated with its CNV at FDR <0.05, the CNV is a *cis*-CNV. (4) *cis*-miRNA-gene pairs for mapping miRNA and gene expression profiles: a small portion of miRNAs are embedded in gene regions (i.e. host genes) and frequently co-transcribed with host genes [14, 15]. If the expression levels of a miRNA and its host gene are associated at FDR <0.05, the pair is a *cis*-miRNA-gene pair. (5) *cis*-mRNA-protein pairs for mapping protein and gene expression profiles: the abundance of a protein depends on the corresponding mRNA transcript level and other factors [17]. If their association is significant at FDR <0.05, the pair is a *cis*-mRNA-protein pair.

Multi-Omics Data matcher (MODMatcher)

In the "Determine self-aligned vs. cross-aligned" step (**Figure 1E**), the similarity scores of self-aligned profiles between type A and type B, $S(A_i, B_i)$, were top 5% ranked among $S(A_n, B_i), n = 1 \dots N_A$ as well as $S(A_i, B_n), n = 1 \dots N_B$, to be annotated as *self-aligned*, where N_A and N_B represent the number of samples of type A and type B, respectively. If the sample sizes were bigger than 400, top 20 was used as the threshold for self-alignment. Next, for the profiles that were not self-aligned, reciprocal mapping was applied to find any potential matches among

other unmatched profiles. If sample j of type A and sample k of type B, $S(A_i, B_k)$ is 1st ranked among $S(A_j, B_n), n = 1 \dots N_B$ as well as $S(A_n, B_k), n = 1 \dots N_A$, then the pair is annotated as *cross-aligned*.

A probabilistic Multi-Omics Data matcher (*pro*MODMatcher)

The characteristics (noises, biases, dynamic ranges, and etc.) of two types of profiles may be different. The rank-based cutoff was not able to reflect similarity score differences in a specific similarity score distribution with a large or small variance (**Supplementary Figure S7**). In the "Determine self- vs. cross-aligned" step, the *pro*MODMatcher evaluated a similarity score in a bivariate normal distribution, $\mathrm{X} \sim N_2(\boldsymbol{\mu}, \boldsymbol{\Sigma})$, where $\boldsymbol{\mu}$ is the mean vector and $\boldsymbol{\Sigma}$ is the covariance matrix (**Figure 1D**). The probability of a match between profile i of type A and profile j of type B, $\mathrm{P}(A_i, B_j) = P(S(A_i, B_j), S(A_i, B_j))$, is estimated based on a score distribution of $(S(A_i, B_m), S(A_m, B_j))$, where A_m and B_m represent type A and type B profile of the mth matched profile pairs, respectively. Given the bivariate normal distribution, we calculated the distance of a point $x = (S(A_i, B_m), S(A_m, B_j))$ to the center of the distribution, known as Mahalanobis distance, as $r = \sqrt{(x - \boldsymbol{\mu})^T \Sigma^{-1} (x - \boldsymbol{\mu})}$, and the cumulative function $F(R \le r) = 1 - e^{-r^2/2}$. To obtain a more robust estimation of covariance matrix $\boldsymbol{\Sigma}$ of the distribution, we added 1000 profile pairs of randomly permuted profiles in addition to true profile pairs.

Additionally, we introduced a prior probability of self-alignment p_0. Thus, given profiles A_i and B_j and their similarity score $S(A_i, B_j)$ as well as estimated Mahalanobis distance $r_{i,j}$, we calculated the p-value of the two profiles matched by chance as $p(A_i, B_j) = \begin{cases} p_0 * e^{-r_{i,j}^2/2}, if\ i = j \\ e^{-r_{i,j}^2/2}, if\ i \ne j \end{cases}$. In this study, the prior probability p_0 was set as $p_0 = 1/N_s$, where N_s

represents number of samples. We also set global similarity score cutoffs for self-alignment, S_{self}^{cutoff}, as well as cross-alignment, S_{cross}^{cutoff}. The S_{self}^{cutoff} value was set as the lower bound of 99% of the self-self similarity scores estimated by mean and standard deviations of $S(A_i, B_i)$, where *i* indicates the samples with both type A and Type B profiles. And the S_{cross}^{cutoff} was set as the lower bound of 68% of the self-self similarity scores.

The similarity score $S(A_i, B_j)$ and its corresponding p-value $p(A_i, B_j)$ were used to identify matched pairs between type A and type B profiles (**Figure 1E**). Each round of our procedure consisted of three steps. First, the self-alignment similarity score $S(A_i, B_i)$ and corresponding p-value $p(A_i, B_i)$ were calculated. If $S(A_i, B_i) > S_{self}^{cutoff}$ and $(A_i, B_i) < p_{i \neq j}(A_i, B_j)$, then the profiles A_i and B_i were self-aligned. Second, for a profile A_i that was not self-aligned to the profile B_i in the first step, it was compared to all unmapped profile B_j. If the similarity score $S(A_i, B_j) < S_{cross}^{cutoff}$ and the corresponding p-value $p(A_i, B_j) \leq arg \min_{n \in [1 \ldots, N_B]}(p(A_i, B_n))$ and $p(A_i, B_j) \leq arg \min_{n \in [1 \ldots, N_A]}(p(A_n, B_j))$, then the profiles A_i and B_j were cross-aligned. Third, for profile pairs A_i and B_i that were not aligned in the first two steps, if $S(A_i, B_i) > S_{self}^{cutoff}$ and the p-value $p(A_i, B_i)$ was smaller than the fifth smallest among $p(A_i, B_n), n = 1 \ldots N_B$ as well as $p(A_n, B_i), n = 1 \ldots N_A$, then the profiles A_i and B_i were rescued as self-aligned. The rounds of alignments were repeated until there was no further change.

<u>Correlation of cis-associated mRNA and miRNA before and after correcting labeling errors</u>

To assess improvement of signals after labeling error correction, we calculated Spearman correlation between miRNA expression and its host genes with initially matched pairs based on sample ID and with aligned sample pairs. To avoid bias due to different number of samples, we

matched the number of samples of initially matched pairs to the number of aligned pairs. We randomly selected the samples with the same number of aligned pairs, and calculated the Spearman correlation. We performed random selection 100 times and calculated mean of correlation.

Availability of source code and requirements

Project name: ProMODMatcher (passcode to decrypt the zipped file is “password123”)

Project home page: Github site (https://github.com/integrativenetworkbiology/proMODMatcher) and http://research.mssm.edu/integrative-network-biology/Software.html

Operating system: Platform independent

Programming language: R (R 3.5.1 or later)

Other requirements: R package mnormt

License: GNU General Public License

RRID: SCR_017219

Availability of supporting data and materials

Data supporting the results of this article are deposited in Data supporting the results of this article are publicly available at firehose database, TCGA data portal, and ICGC data portal (see Data Description). Data further supporting this work and snapshots of our code are available in the *GigaScience* repository, GigaDB [18].

Declarations

List of abbreviations

TCGA: The Cancer Genome Atlas

QC: quality control

MODMatcher: Multi-Omics Data matcher

*pro*MODMatcher : A probabilistic Multi-Omics Data matcher

BH: Benjamini-Hochberg

FPR: false positive rate

RPPA: Reverse Phase Protein Array

CNV: Copy number variation

HM27: Illumina HumanMethylation27 Beadchip

HM450: Illumina HumanMethylation450 Beadchip

BRCA: breast invasive carcinoma

BLCA: Bladder urothelial carcinoma

CESC: Cervical and endocervical cancers

COAD: Colon adenocarcinoma

DLBC: Lymphoid Neoplasm Diffuse Large B-cell Lymphoma

GBM: Glioblastoma multiforme

HNSC: Head and Neck squamous cell carcinoma

KIRC: Kidney renal clear cell carcinoma

KIRP: Kidney renal papillary cell carcinoma

LGG: Brain Lower Grade Glioma

LIHC: Liver hepatocellular carcinoma

LUAD: Lung adenocarcinoma

LUSC: Lung squamous cell carcinoma

OV: Ovarian serous cystadenocarcinoma

PRAD: Prostate adenocarcinoma

READ: Rectum adenocarcinoma

SARC: Sarcoma

SKCM: Skin Cutaneous Melanoma

STAD: Stomach adenocarcinoma

THCA: Thyroid carcinoma

UCEC: Uterine Corpus Endometrial Carcinoma

Consent for publication

Not applicable.

Competing interests

The authors declare that they have no competing interests.

Funding

This work was partially supported by National Institutes of Health [grant numbers R01-AG046170, U01-HG008451, and U19-AI118610].

Authors' contributions

EL and JZ designed research. EL performed research and analyzed data. SY contributed to download data and analyzed data by MODMatcher method. WW contributed design of simulation. ZT contributed revising paper. EL and JZ wrote the paper. All authors read and approved the final manuscript.

Acknowledgements

We thank members of Zhu laboratory for discussions.

REFERENCES

1. Chen Y, Zhu J, Lum PY, Yang X, Pinto S, MacNeil DJ, et al. Variations in DNA elucidate molecular networks that cause disease. Nature. 2008;452 7186:429-35.
2. Cancer Genome Atlas N. Comprehensive molecular portraits of human breast tumours. Nature. 2012;490 7418:61-70. doi:10.1038/nature11412.
3. Lee E, de Ridder J, Kool J, Wessels LF and Bussemaker HJ. Identifying regulatory mechanisms underlying tumorigenesis using locus expression signature analysis. Proceedings of the National Academy of Sciences of the United States of America. 2014;111 15:5747-52. doi:10.1073/pnas.1309293111.
4. Zhong H, Beaulaurier J, Lum PY, Molony C, Yang X, Macneil DJ, et al. Liver and adipose expression associated SNPs are enriched for association to type 2 diabetes. PLoS Genet. 2010;6 5:e1000932. doi:10.1371/journal.pgen.1000932.
5. Schadt EE, Molony C, Chudin E, Hao K, Yang X, Lum PY, et al. Mapping the genetic architecture of gene expression in human liver. PLoS Biol. 2008;6 5:e107.
6. Hsu YH, Zillikens MC, Wilson SG, Farber CR, Demissie S, Soranzo N, et al. An integration of genome-wide association study and gene expression profiling to prioritize the discovery of novel susceptibility Loci for osteoporosis-related traits. PLoS genetics. 2010;6 6:e1000977. doi:10.1371/journal.pgen.1000977.
7. Westra HJ, Jansen RC, Fehrmann RS, te Meerman GJ, van Heel D, Wijmenga C, et al. MixupMapper: correcting sample mix-ups in genome-wide datasets increases power to detect small genetic effects. Bioinformatics. 2011;27 15:2104-11. doi:btr323 [pii]

10.1093/bioinformatics/btr323.

8. Yoo S, Huang T, Campbell JD, Lee E, Tu Z, Geraci MW, et al. MODMatcher: multi-omics data matcher for integrative genomic analysis. PLoS Comput Biol. 2014;10 8:e1003790. doi:10.1371/journal.pcbi.1003790.
9. Cibulskis K, McKenna A, Fennell T, Banks E, DePristo M and Getz G. ContEst: estimating cross-contamination of human samples in next-generation sequencing data. Bioinformatics. 2011;27 18:2601-2. doi:10.1093/bioinformatics/btr446.
10. Bergmann EA, Chen BJ, Arora K, Vacic V and Zody MC. Conpair: concordance and contamination estimator for matched tumor-normal pairs. Bioinformatics. 2016;32 20:3196-8. doi:10.1093/bioinformatics/btw389.
11. Brem RB, Yvert G, Clinton R and Kruglyak L. Genetic dissection of transcriptional regulation in budding yeast. Science. 2002;296 5568:752-5. doi:10.1126/science.1069516.
12. Ball MP, Li JB, Gao Y, Lee JH, LeProust EM, Park IH, et al. Targeted and genome-scale strategies reveal gene-body methylation signatures in human cells. Nat Biotechnol. 2009;27 4:361-8. doi:10.1038/nbt.1533.
13. Bolstad BM, Irizarry RA, Astrand M and Speed TP. A comparison of normalization methods for high density oligonucleotide array data based on variance and bias. Bioinformatics. 2003;19 2:185-93. doi:10.1093/bioinformatics/19.2.185.
14. Baskerville S and Bartel DP. Microarray profiling of microRNAs reveals frequent coexpression with neighboring miRNAs and host genes. RNA. 2005;11 3:241-7. doi:10.1261/rna.7240905.

15. Rodriguez A, Griffiths-Jones S, Ashurst JL and Bradley A. Identification of mammalian microRNA host genes and transcription units. Genome Res. 2004;14 10A:1902-10. doi:10.1101/gr.2722704.

16. Stranger BE, Forrest MS, Dunning M, Ingle CE, Beazley C, Thorne N, et al. Relative impact of nucleotide and copy number variation on gene expression phenotypes. Science. 2007;315 5813:848-53. doi:10.1126/science.1136678.

17. de Sousa Abreu R, Penalva LO, Marcotte EM and Vogel C. Global signatures of protein and mRNA expression levels. Mol Biosyst. 2009;5 12:1512-26. doi:10.1039/b908315d.

18. Lee E; Yoo S; Wang W; Tu Z; Zhu J: Supporting data for "A probabilistic multi-omics data matching method for detecting sample errors in integrative analysis" GigaScience Database. 2019. http://dx.doi.org/10.5524/100616.

Figure legends

Figure 1. Overview of *pro*MODMatcher procedure. (A) Probes in two types of profiles (i.e. Type A and Type B) were matched by intrinsic biological relationships. **(B)** The significant *cis*-associations from two different data types were identified by the Spearman correlation. The data for each *cis* relationship was normal rank-transformed. (**C**) The sample similarity score between the two types of data $S(A_i, B_j)$ is defined as Spearman correlation between normal rank-transformed profiles. **(D)** The *pro*MODMatcher evaluated a similarity score of a match, $S(A_i, B_j)$, by calculating probability of a match estimated based on a score distribution of $\left(S(A_i, B_n), S(A_n, B_j)\right)$, where A_n and B_n represent type A and type B profile of the nth matched profile pairs. **(E)** In the Determine self-aligned vs. cross-aligned step, profile pairs matched by sample IDs were checked whether their similarity scores were high to be annotated as "self-aligned". If not, additional steps were applied to find any potential matches among other unmatched profiles. The matched profile pairs were used to update significant *cis*-associations.

Figure 2. Application of MODMatcher to simulated data sets. We simulated data sets with different numbers of samples and significant *cis*-associations. For variable number of samples and significant *cis*-associations, sensitivity and false positive rate (FPR, 1-specificity) were measured and plotted.

Figure 3. Application of *pro*MODMatcher to simulated data sets. (A) For variable number of samples and significant *cis*-associations specificity and FPR were measured based on simulated data sets with 0%, 4% and 6% sample labeling error rate. **(B-C)** F measure, sensitivity, and specificity were compared with MODMatcher's results.

Figure 4. Aligning gene expression profiles by RNAseq and miRNAseq data. (A) An example of miRNAs (e.g. miR-452) that are embedded in gene regions (e.g. *GABRE*). **(B)** Expression level of miR-452 was highly associated with mRNA expression of *GABRE.* **(C)** The rank of the similarity scores of self-self RNAseq-miRNAseq profiles. **(D)** An example of the similarity score of the self-aligned profiles, TCGA−D8−A1JH-01. The similarity score between RNAseq profile of TCGA−D8−A1JH-01 and miRNA profiles of other samples were shown. The red star indicates similarity score of self-self RNAseq-miRNAseq profiles. **(E)** An example of non self-aligned RNAseq-miRNA profiles, TCGA-B6-A0X7-01. **(F)** The probabilities of similarity scores (before multiplying prior probability) for self-aligned RNAseq-miRNAseq profiles. **(G)** An example of similarity scores of self-aligned RNAseq-miRNA profile pairs. X-axis indicates the similarity scores between RNAseq profile of TCGA-OL-A6VO-01 and miRNAseq profiles of all other samples, and y-axis indicates similarity scores between miRNAseq profile of TCGA-OL-A6VO-01 and RNAseq profiles of all other samples. The red dot indicates similarity score for self-self RNAseq-miRNAseq profile. **(H)** An example of similarity scores of non self-aligned RNAseq-miRNA profile pairs.

Figure 5. Comparison of MODMatcher and *pro*MODMatcher for aligning expression profiles by RNAseq and miRNAseq data. (A) The similarity scores of a self-aligned RNAseq-miRNA profile pair identified by proMODMatcher, but not by MODMatcher. X-axis indicates the similarity score between RNAseq profile of TCGA-AO-A0JF-01 and miRNAseq profiles of all other samples, and y-axis indicates similarity score between miRNAseq profile of TCGA-AO-A0JF-01 and RNAseq profiles of all other samples. The red dot indicates similarity score for self-self RNAseq-miRNAseq profiles. **(B)** One cross-aligned pair, RNAseq of TCGA-BH-A0BZ-

01 and miRNA of TCGA-E2-A15K-01, identified by *pro*MODMatcher. The similarity score of the cross-aligned pair was shown in blue and the similarity scores of self-self alignments was shown in red. **(C)** Significance levels of *cis*-associations based on profile pairs aligned by MODMatcher and *pro*MODMatcher.

Figure 6. Aligning gene expression profiles by Agilent array and miRNAseq data (A) An example of possible sample swaps. In alignment of Agilent array and miRNAseq profiles, TCGA-BH-A18K-01 and TCGA-BH-A18T-01 were cross-aligned to each other. The similarity scores of each cross-alignment were shown. The similarity score of the cross-aligned pair was shown in blue and the similarity scores of self-self alignments were shown in red. **(B)** Other omics profiles of TCGA-BH-A18K and TCGA-BH-A18T were compared with each other and results were summarized into a patient-centric view. Red line indicates self-aligned, and blue line indicates cross-aligned. **(C)** After swapping the corresponding mRNA Agilent array profiles, multiple-omics profiles of TCGA-BH-A18K and TCGA-BH-A18T were aligned to each other consistently. **(D-F)** The similarity scores of other cross-aligned pairs were shown, and their available omics profiles and alignment results were summarized into a patient-centric view.

Figure 7. Aligning mRNA and RPPA profiles. **(A)** The Spearman correlations of protein abundance and the corresponding mRNA's expression level were shown based on RNAseq and Agilent array. The red line indicates correlation values corresponding to q-value 0.05. **(B)** Similarity scores of a self-aligned RNAseq-miRNA profile pair **(C)** Similarity scores of a cross-aligned RNAseq-miRNA profile pair. **(D)** Similarity scores of the cross-aligned pair between the mRNA Agilent microarray and RPPA profiles, TCGA-AR-A1AV-01 and TCGA-AR-A1AW-01, and alignment results for other omics profiles of this pair into a patient centric view.

Figure 8. Application to TCGA pan-cancer datasets. (A-B) The self-alignment rate of RNA-miRNA and RNA-RPPA alignment for each cancer type. **(C-D)** Two possible sample swap cases of miRNA profiles in the TCGA UCEC and OV datasets. The similarity scores of each cross-alignment and alignment results for other available omics profiles were shown.

Figure 9. Application to ICGC datasets (A) An example of self-self aligned, non self-self aligned and cross-aligned pairs of samples based on alignment between Array and CNV profiles in the NBL-US dataset. (**B**) An example of sample labeling errors. In alignment of Array and DNA methylation profiles, DO7484 and DO7472 were cross-aligned to each other. The similarity scores of each cross-alignment are shown. The similarity score of the cross-aligned pair is shown in blue and the similarity scores of self-self alignments are shown in red. Omics profiles of DO7484 and DO7472 were compared with each other and results were summarized into a patient-centric view. Red line indicates self-aligned, and blue line indicates cross-aligned. **(C)** An example of possible sample swaps and sample labeling errors. DO229525 and DO51109 were cross-aligned to each other in alignment of RNAseq and DNA methylation profiles as well as Array and DNA methylation profiles. Additionally, RNAseq and Array profiles of DO51105 were cross-aligned to DNA methylation profile of DO51091. (**D**) Other omics profiles of these pairs were compared with each other and results were summarized into a patient-centric view. After swapping the corresponding DNA methylation profiles, multiple-omics profiles of DO229525 and DO51109 were aligned to each other consistently.

Figure 10. Correcting sample labeling errors. (A) Mis-labeled samples were outliers when comparing significant pairs between mRNA and miRNA expression levels in the TCGA BRCA dataset. Red dots were mis-labeled samples. **(B)** Spearman correlation between expression levels of miRNAs and their host genes before and after curating sample labeling errors.

Table 3. Application of *pro*MODMatcher to mRNA and miRNA profiles of TCGA cancer data excluding BRCA.

Types of cancer	Data types	Data types	# Common samples	# cis pair	# of self-aligned	# of cross-aligned	Cross-aligned pairs	Self in RNA-CNV	Cross-aligned pairs
	Type1	Type 2					Type 1		Type 2
BLCA	RNAseq	miRNAseq	405	187/231	402 (99.2%)	0			
CESC	RNAseq	miRNAseq	100	132/223	100 (100%)	0			
COAD	RNAseq	miRNAseq	248	122/191	242 (97.5%)	8 (3.2%)	TCGA-CM-4744-01	Y	TCGA-AA-3558-01
							TCGA-QL-A97D-01	Y	TCGA-AA-A00W-01
							TCGA-A6-A567-01	Y	TCGA-AA-3693-01
							TCGA-5M-AATA-01	Y	TCGA-AA-3529-01
							TCGA-RU-A8FL-01	Y	TCGA-AZ-4681-01
							TCGA-QG-A5YV-01	Y	TCGA-AA-A02H-01
							TCGA-A6-A565-01	Y	TCGA-AA-A02E-01
							TCGA-5M-AATE-01	Y	TCGA-AA-A01F-01
DLBC	RNAseq	miRNAseq	47	59/210	47 (100%)	0 (0%)			
GBM	Agilent	miRNA array	525	73/107	307 (58.4%)	14(2.6%)	TCGA-02-0064-01	Y	TCGA-08-0390-01
							TCGA-02-0325-01	Y	TCGA-08-0345-01
							TCGA-02-0321-01	Y	TCGA-19-0957-01
							TCGA-08-0510-01	Y	TCGA-26-5135-01
							TCGA-02-0070-01	Y	TCGA-28-5218-01
							TCGA-12-0773-01	Y	TCGA-06-0744-01
							TCGA-12-0780-01	Y	TCGA-08-0354-01
							TCGA-12-0822-01	Y	TCGA-16-1045-01
							TCGA-16-1062-01	Y	TCGA-28-5209-01
							TCGA-14-1829-01	Y	TCGA-14-1450-01
							TCGA-19-1385-01	Y	TCGA-08-0352-01
							TCGA-32-4719-01	Y	TCGA-06-0140-01
							TCGA-19-5952-01	Y	TCGA-02-0324-01
							TCGA-06-0201-01	No	TCGA-06-0141-01
	HG-U133	miRNA array	520	56/100	315 (60.5%)	5 (0.9%)	TCGA-02-0058-01	No: TCGA-06-0190-01	TCGA-12-0778-01

							TCGA-02-0115-01	Y	TCGA-12-0656-01
							TCGA-19-1789-01	Y	TCGA-06-0413-01
							TCGA-06-2561-01	Y	TCGA-12-0691-01
							TCGA-02-0338-01	Y	TCGA-76-6283-01
	RNAseq	miRNA array	151	70/129	115 (76.1%)	19 (12.5%)	TCGA-06-1804-01	Y	TCGA-81-5911-01
							TCGA-06-0178-01	No	TCGA-16-1060-01
							TCGA-14-1034-01	Y	TCGA-02-0330-01
							TCGA-15-0742-01	Y	TCGA-02-0116-01
							TCGA-06-5413-01	Y	TCGA-14-0865-01
							TCGA-19-2620-01	Y	TCGA-76-6193-01
							TCGA-06-0158-01	Y	TCGA-06-0174-01
							TCGA-06-0211-01	Y	TCGA-12-3648-01
							TCGA-06-2564-01	Y	TCGA-12-0688-01
							TCGA-06-0141-01	Y	TCGA-08-0246-01
							TCGA-06-0238-01	Y	TCGA-06-0177-01
							TCGA-06-0744-01	Y	TCGA-76-6664-01
							TCGA-06-0125-01	Y	TCGA-08-0358-01
							TCGA-41-2572-01	Y	TCGA-02-0021-01
							TCGA-06-0190-02	Y	TCGA-19-5955-01
							TCGA-28-2499-01	No: TCGA-02-0099-01	TCGA-12-1091-01
							TCGA-06-0152-02	Y	TCGA-26-1799-01
							TCGA-19-1389-02	Y	TCGA-14-0813-01
							TCGA-14-1034-02	Y	TCGA-15-1447-01
HNSC	RNAseq	miRNAseq	517	183/229	494 (95.5%)	0 (0%)			
KIRC	RNAseq	miRNAseq	516	146/205	487 (94.3%)	0 (0%)			
KIRP	RNAseq	miRNAseq	290	131/205	285 (98.2%)	0 (0%)			
LAML	RNAseq	miRNAseq	173	93/166	168 (97.1%)	0			
LGG	RNAseq	miRNAseq	526	170/245	500 (95.0%)	0			
LIHC	RNAseq	miRNAseq	369	179/228	369 (99.4%)	0			
LUAD	RNAseq	miRNAseq	512	179/229	507 (99.0%)	0			
	Agilent	miRNAseq	32	32/180	17 (53.1%)	3 (9.3%)	TCGA-44-2655-01	Y	TCGA-44-6148-01
							TCGA-05-4249-01	No	TCGA-86-A4D0-01
							TCGA-35-4123-01	No	TCGA-55-6969-01
LUSC	RNAseq	miRNAseq	474	191/229	466 (98.3%)	0 (0%)			

OV	RNAseq	miRNAseq	291	159/192	282 (96.9%)	5 (1.7%)	TCGA-24-2261-01	Y	TCGA-31-1953-01
							TCGA-31-1953-01	Y	TCGA-24-2261-01
							TCGA-61-1728-01	Y	TCGA-23-2072-01
							TCGA-09-0369-01	Y	TCGA-25-1877-01
							TCGA-VG-A8LO-01	Y	TCGA-04-1654-01
PRAD	RNAseq	miRNAseq	494	129/198	432 (87.4%)	0			
READ	RNAseq	miRNAseq	66	77/180	60 (90.9%)	3 (4.5%)	TCGA-AG-A01J-01	Y	TCGA-DY-A1DG-01
							TCGA-AG-A014-01	Y	TCGA-DC-6158-01
							TCGA-AG-A023-01	Y	TCGA-AG-4022-01
SARC	RNAseq	miRNAseq	261	169/220	261 (100%)	0			
SKCM	RNAseq	miRNAseq	449	203/251	446 (99.3%)	0			
STAD	RNAseq	miRNAseq	377	193/256	371 (98.4%)	0			
THCA	RNAseq	miRNAseq	508	139/217	483 (95.0%)	0			
UCEC	RNAseq	miRNAseq	361	169/240	354 (98.0%)	4 (1.1%)	TCGA-A5-A0GP-01	Y	TCGA-AJ-A2QO-01
							TCGA-AX-A1C4-01	Y	TCGA-AX-A1CI-01
							TCGA-AX-A1CI-01	Y	TCGA-AX-A1C4-01
							TCGA-BG-A220-01	No	TCGA-AJ-A3NE-01

Underlines indicates sample swaps

Table 4. Application of *pro*MODMatcher to mRNA and RPPA profiles of TCGA cancer data excluding BRCA

Types of cancer	Data types	Data types	# Common samples	# cis pair	# of self-aligned	# of cross-aligned	Cross-aligned pairs	Self in RNA-CNV	Cross-aligned pairs
	Type1	Type 2	Type 1				Type 1		Type 2
BLCA	RNAseq	RPPA	340	121/193	297 (87.3%)	3 (0.8%)	TCGA-XF-AAN8-01	Y	TCGA-FD-A6TB-01
							TCGA-FD-A5BR-01	Y	TCGA-XF-AAMF-01
							TCGA-E7-A6ME-01	Y	TCGA-E7-A541-01
CESC	RNAseq	RPPA	172	101/184	152 (88.8%)	1 (0.5%)	TCGA-EK-A3GJ-01	Y	TCGA-C5-A8XI-01
COAD	RNAseq	RPPA	240	110/202	195 (81.2%)	15 (6.2%)	TCGA-G4-6321-01	Y	TCGA-AA-A01P-01
							TCGA-AD-A5EJ-01	Y	TCGA-AA-3672-01
							TCGA-CA-5256-01	Y	TCGA-AA-3815-01
							TCGA-AZ-4682-01	Y	TCGA-G4-6321-01
							TCGA-G4-6303-01	Y	TCGA-A6-2677-01
							TCGA-A6-6137-01	Y	TCGA-AA-A01S-01
							TCGA-G4-6627-01	Y	TCGA-G4-6298-01
							TCGA-A6-6140-01	Y	TCGA-AA-3519-01
							TCGA-NH-A5IV-01	Y	TCGA-AA-A00E-01
							TCGA-G4-6320-01	Y	TCGA-A6-2672-01
							TCGA-DM-A28H-01	Y	TCGA-AA-3811-01
							TCGA-CK-5913-01	Y	TCGA-AA-3664-01
							TCGA-NH-A50U-01	Y	TCGA-AA-3558-01
							TCGA-AD-6901-01	Y	TCGA-NH-A6GC-06
							TCGA-A6-A565-01	Y	TCGA-AA-3520-01
DLBC	RNAseq	RPPA	33	58/184	32 (96.9%)	0 (0%)			
GBM	Agilent	RPPA	191	97/194	157 (82.1%)	13 (6.8%)	TCGA-06-0139-01	No	TCGA-06-A5U1-01
							TCGA-06-0158-01	Y	TCGA-19-5950-01
							TCGA-06-0176-01	Y	TCGA-19-2625-01
							TCGA-06-0206-01	Y	TCGA-06-0190-02
							TCGA-12-0620-01	Y	TCGA-RR-A6KC-01
							TCGA-06-0881-01	Y	TCGA-02-0003-01

							TCGA-14-1454-01	Y	TCGA-19-A6J5-01
							**TCGA-12-1091-01**	**Y**	**TCGA-14-1034-02**
							TCGA-14-1037-01	No	TCGA-19-A60I-01
							TCGA-14-1795-01	Y	TCGA-12-5301-01
							TCGA-32-2616-01	Y	TCGA-06-5858-01
							TCGA-81-5911-01	Y	TCGA-19-1389-02
							TCGA-14-1450-01	Y	TCGA-06-5418-01
	HG-U133	RPPA	186	90/187	147 (79.0%)	13 (6.9%)	TCGA-02-0068-01	Y	TCGA-06-5413-01
							TCGA-02-0033-01	No	TCGA-32-4211-01
							TCGA-14-0781-01	Y	TCGA-74-6575-01
							**TCGA-12-1091-01**	**Y**	**TCGA-14-1034-02**
							TCGA-28-2509-01	Y	TCGA-19-A60I-01
							TCGA-06-0141-01	Y	TCGA-06-A5U1-01
							TCGA-06-0160-01	Y	TCGA-06-6700-01
							TCGA-06-0394-01	Y	TCGA-74-6578-01
							TCGA-08-0518-01	Y	TCGA-26-6173-01
							TCGA-08-0512-01	Y	TCGA-19-1389-02
							TCGA-02-0330-01	Y	TCGA-06-A6S1-01
							TCGA-32-2491-01	Y	TCGA-06-6698-01
							TCGA-32-4719-01	Y	TCGA-06-0876-01
	RNAseq	RPPA	83	106/201	75 (90.3%)	25			
HNSC	RNAseq	RPPA	212	82/156	175 (82.5%)	3 (1.4%)	TCGA-CQ-6222-01	No	TCGA-CV-5439-01
							TCGA-D6-6824-01	Y	TCGA-CV-5976-01
							TCGA-MZ-A7D7-01	Y	TCGA-CN-6011-01
KIRC	RNAseq	RPPA	475	125/209	396 (83.3%)	4 (0.8%)	TCGA-CJ-5681-01	Y	TCGA-B0-5709-01
							TCGA-B0-5709-01	Y	TCGA-CJ-6030-01
							TCGA-CJ-4869-01	Y	TCGA-BP-4771-01
							TCGA-CJ-4888-01	Y	TCGA-CJ-4875-01
KIRP	RNAseq	RPPA	215	93/184	178 (82.7%)	3 (1.3%)	TCGA-KV-A74V-01	Y	TCGA-MH-A55Z-01
							TCGA-MH-A854-01	Y	TCGA-UZ-A9PL-01
							TCGA-MH-A561-01	Y	TCGA-B1-A47N-01
LGG	RNAseq	RPPA	435	95/173	320 (73.5%)	1 (0.2%)	TCGA-HT-7681-01	Y	TCGA-P5-A737-01
LIHC	RNAseq	RPPA	181	105/214	158 (87.2%)	4 (2.2%)	TCGA-ZS-A9CD-01	Y	TCGA-G3-A5SK-01
							TCGA-DD-AAC9-01	Y	TCGA-DD-A4NG-01
							TCGA-G3-AAV0-01	Y	TCGA-GJ-A9DB-01

							TCGA-G3-AAV5-01	Y	TCGA-ED-A627-01
LUAD	RNAseq	RPPA	360	125/193	312 (86.6%)	10 (2.7%)	TCGA-50-5045-01	No	TCGA-44-7672-01
							TCGA-44-7667-01	Y	TCGA-44-3917-01
							TCGA-MP-A4TI-01	Y	TCGA-MP-A4TA-01
							TCGA-MP-A4TJ-01	Y	TCGA-50-5939-01
							TCGA-50-5055-01	No	TCGA-97-A4M2-01
							TCGA-55-A48X-01	Y	TCGA-64-5778-01
							TCGA-64-5775-01	No	TCGA-05-5715-01
							TCGA-55-6987-01	Y	TCGA-44-2664-01
							TCGA-38-7271-01	Y	TCGA-50-5068-01
							TCGA-55-8208-01	Y	TCGA-50-5066-01
	Agilent	RPPA	23	34/187	14 (60.8%)	7 (30.4%)	TCGA-44-2661-01	No	TCGA-05-4249-01
							TCGA-05-4249-01	No	TCGA-55-6978-01
							TCGA-44-3398-01	No	TCGA-86-A4JF-01
							TCGA-44-4112-01	No	TCGA-44-3919-01
							TCGA-44-2662-01	Y	TCGA-78-7145-01
							TCGA-67-3774-01	Y	TCGA-73-7498-01
							TCGA-35-3621-01	No	TCGA-44-2661-01
LUSC	RNAseq	RPPA	324	125/193	278 (85.8%)	3 (0.9%)	TCGA-18-4086-01	Y	TCGA-63-5131-01
							TCGA-39-5039-01	Y	TCGA-34-2604-01
							TCGA-56-A4ZJ-01	Y	TCGA-90-6837-01
OV	RNAseq	RPPA	241	134/202	232 (96.2%)	9 (3.7%)	TCGA-61-2095-01	Y	TCGA-42-2587-01
							TCGA-09-0364-01	Y	TCGA-29-1774-01
							TCGA-09-2048-01	Y	TCGA-13-0802-01
							TCGA-13-0890-01	Y	TCGA-42-2590-01
							TCGA-24-2035-01	Y	TCGA-30-1892-01
							TCGA-25-1870-01	Y	TCGA-36-2534-01
							TCGA-31-1956-01	Y	TCGA-29-1768-01
							TCGA-57-1583-01	Y	TCGA-61-1916-01
							TCGA-59-2350-01	Y	TCGA-61-1913-01
PRAD	RNAseq	RPPA	351	96/178	209 (59.5%)	9 (2.5%)	TCGA-VN-A88I-01	Y	TCGA-KC-A4BV-01
							TCGA-KC-A7F3-01	Y	TCGA-ZG-A8QX-01
							TCGA-FC-A6HD-01	No	TCGA-EJ-A8FN-01
							TCGA-EJ-5499-01	Y	TCGA-VN-A88L-01
							TCGA-HC-7230-01	Y	TCGA-HC-7748-01

							TCGA-XJ-A83G-01	Y	TCGA-G9-6338-01
							TCGA-HC-A8CY-01	Y	TCGA-V1-A9Z8-01
							TCGA-HC-7821-01	Y	TCGA-YL-A9WL-01
							TCGA-VP-A87C-01	Y	TCGA-EJ-8470-01
READ	RNAseq	RPPA	55	54/202	43 (78.1%)	4 (7.2%)	TCGA-AG-A00H-01	Y	TCGA-F5-6810-01
							TCGA-AG-3584-01	Y	TCGA-AG-4022-01
							TCGA-AG-3883-01	Y	TCGA-AG-4005-01
							TCGA-AG-3575-01	Y	TCGA-F5-6863-01
SARC	RNAseq	RPPA	224	110/184	219 (97.7%)	0			
SKCM	RNAseq	RPPA	352	128/193	314 (89.2%)	2	TCGA-EB-A44N-01	Y	TCGA-EB-A5UM-01
							TCGA-W3-A828-06	Y	TCGA-EB-A551-01
STAD	RNAseq	RPPA	306	103/177	233 (76.1%)	12 (3.9%)	TCGA-D7-6818-01	Y	TCGA-EQ-8122-01
							TCGA-HU-A4H3-01	Y	TCGA-CG-4442-01
							TCGA-SW-A7EB-01	Y	TCGA-CG-4460-01
							TCGA-VQ-A94P-01	Y	TCGA-RD-A8NB-01
							TCGA-ZA-A8F6-01	Y	TCGA-CG-4476-01
							TCGA-FP-8210-01	Y	TCGA-D7-A4Z0-01
							TCGA-HU-8244-01	Y	TCGA-BR-4371-01
							TCGA-HU-8604-01	Y	TCGA-BR-A4QL-01
							TCGA-HU-A4GJ-01	Y	TCGA-CD-A4MI-01
							TCGA-HU-A4H8-01	Y	TCGA-CG-5720-01
							TCGA-R5-A7ZI-01	Y	TCGA-BR-6710-01
							TCGA-VQ-A927-01	Y	TCGA-F1-A72C-01
THCA	RNAseq	RPPA	222	55/167	142 (63.9%)	3 (1.3%)	TCGA-EM-A3FJ-01	No	TCGA-EM-A2CS-06
							TCGA-DJ-A4UW-01	No	TCGA-EL-A3CU-01
							TCGA-ET-A3BQ-01	No	TCGA-EL-A3GR-01
UCEC	RNAseq	RPPA	300	115/187	270 (90%)	15 (5%)	TCGA-AX-A05Y-01	Y	TCGA-AX-A060-01
							TCGA-AX-A05Z-01	Y	TCGA-EO-A3AV-01

							TCGA-AX-A0IW-01	Y	TCGA-KP-A3VZ-01
							TCGA-D1-A163-01	Y	TCGA-AJ-A3BH-01
							TCGA-D1-A1NZ-01	Y	TCGA-E6-A2P9-01
							TCGA-EO-A22T-01	Y	TCGA-B5-A1MW-01
							TCGA-FI-A2F9-01	Y	TCGA-A5-A1OH-01
							TCGA-BG-A0MQ-01	Y	TCGA-A5-A7WJ-01
							TCGA-BG-A0MO-01	Y	TCGA-BK-A13B-01
							TCGA-D1-A17A-01	Y	TCGA-A5-A0GB-01
							TCGA-BS-A0TE-01	Y	TCGA-AJ-A3EK-01
							TCGA-BS-A0UL-01	Y	TCGA-EO-A22T-01
							TCGA-FI-A2CX-01	Y	TCGA-E6-A2P8-01
							TCGA-B5-A11M-01	No	TCGA-EY-A1GW-01
							TCGA-FI-A2D6-01	Y	TCGA-DF-A2KY-01

The **bold** indicates cross-alignments supported by other data.

Figure 1

Click here to access/download;Figure;Figure1_SchematicFigure_01102019.eps

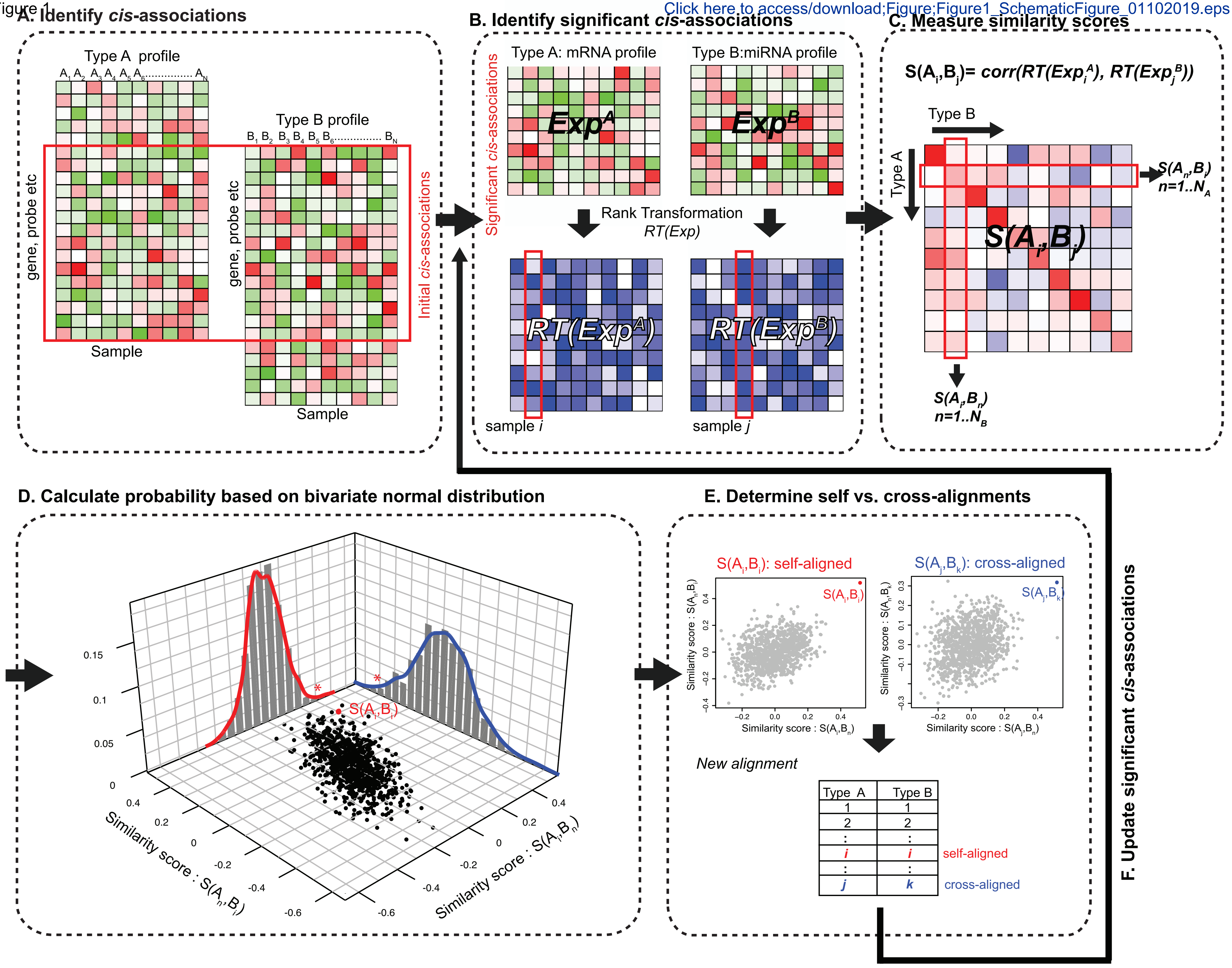

Figure 2

Click here to access/download;Figure;Figure2_Simulation_Updates_01102019.eps

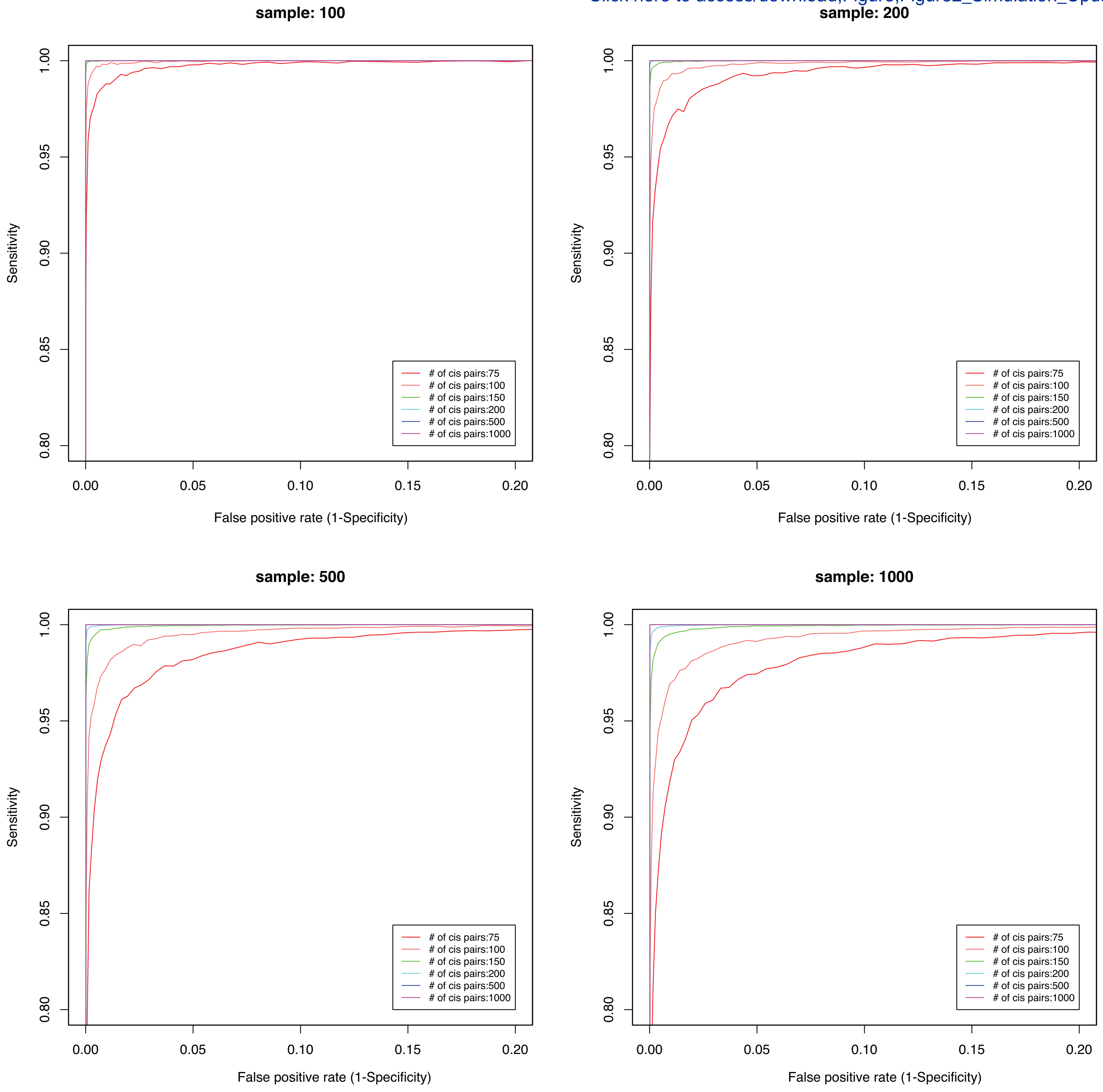

Figure 3

Click here to access/download;Figure;Figure3_MeasurementsCompareMOD_

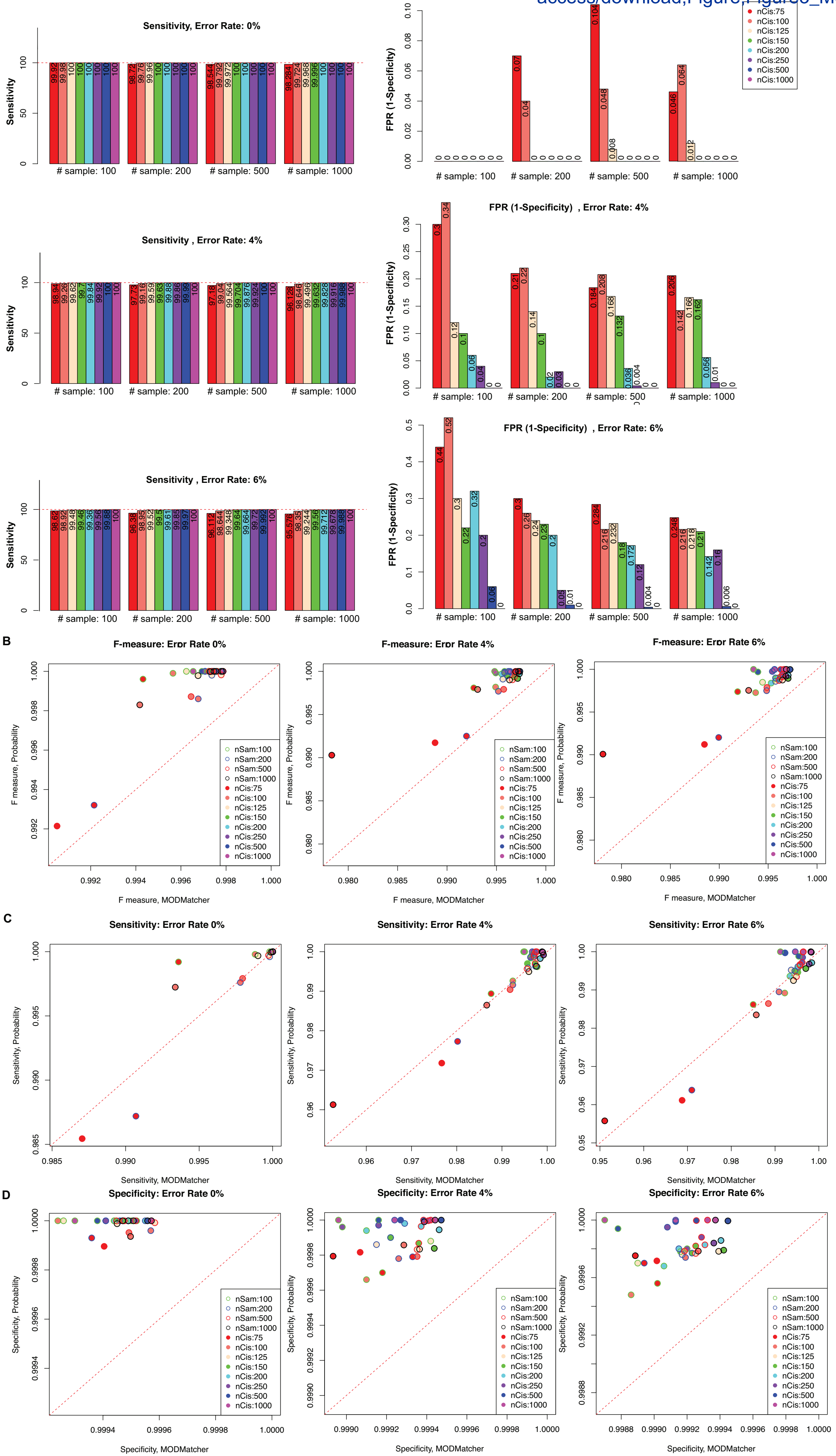

Figure 4

Click here to access/download;Figure;Figure4_SimpleAlignment_01102019.e

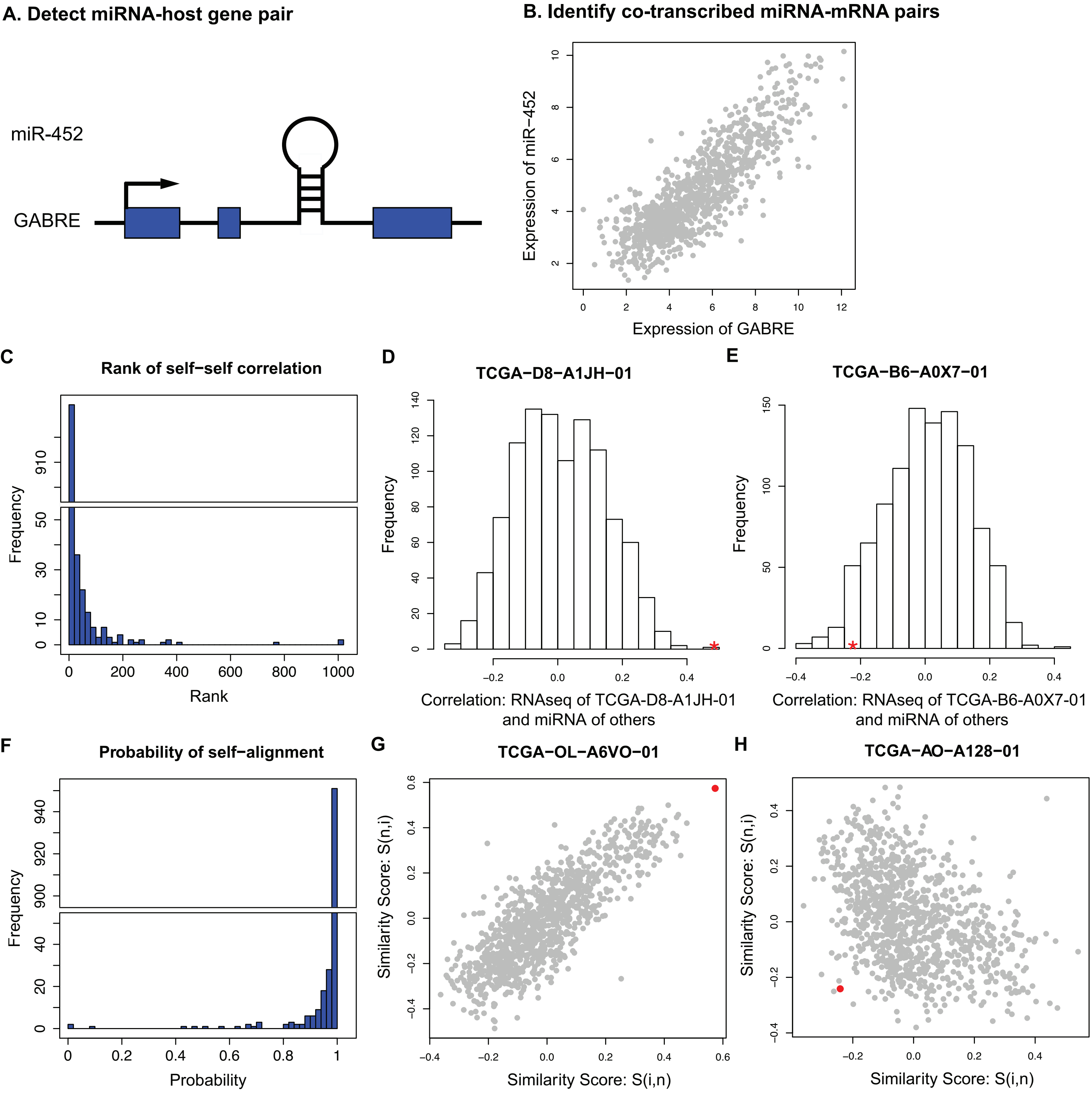

Figure 5

Click here to access/download;Figure;Figure5_SimilarityScore_scatterProb_01102019.eps

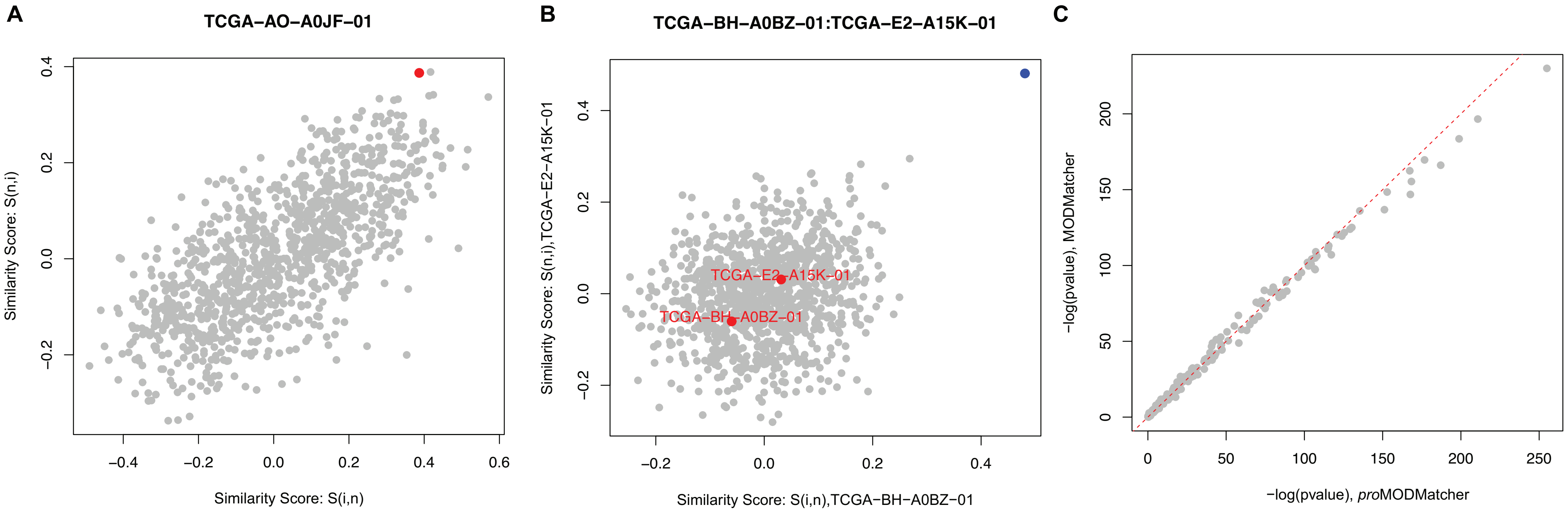

Figure 6

Click here to access/download;Figure;Figure6_SimilarityScore_01102019.eps

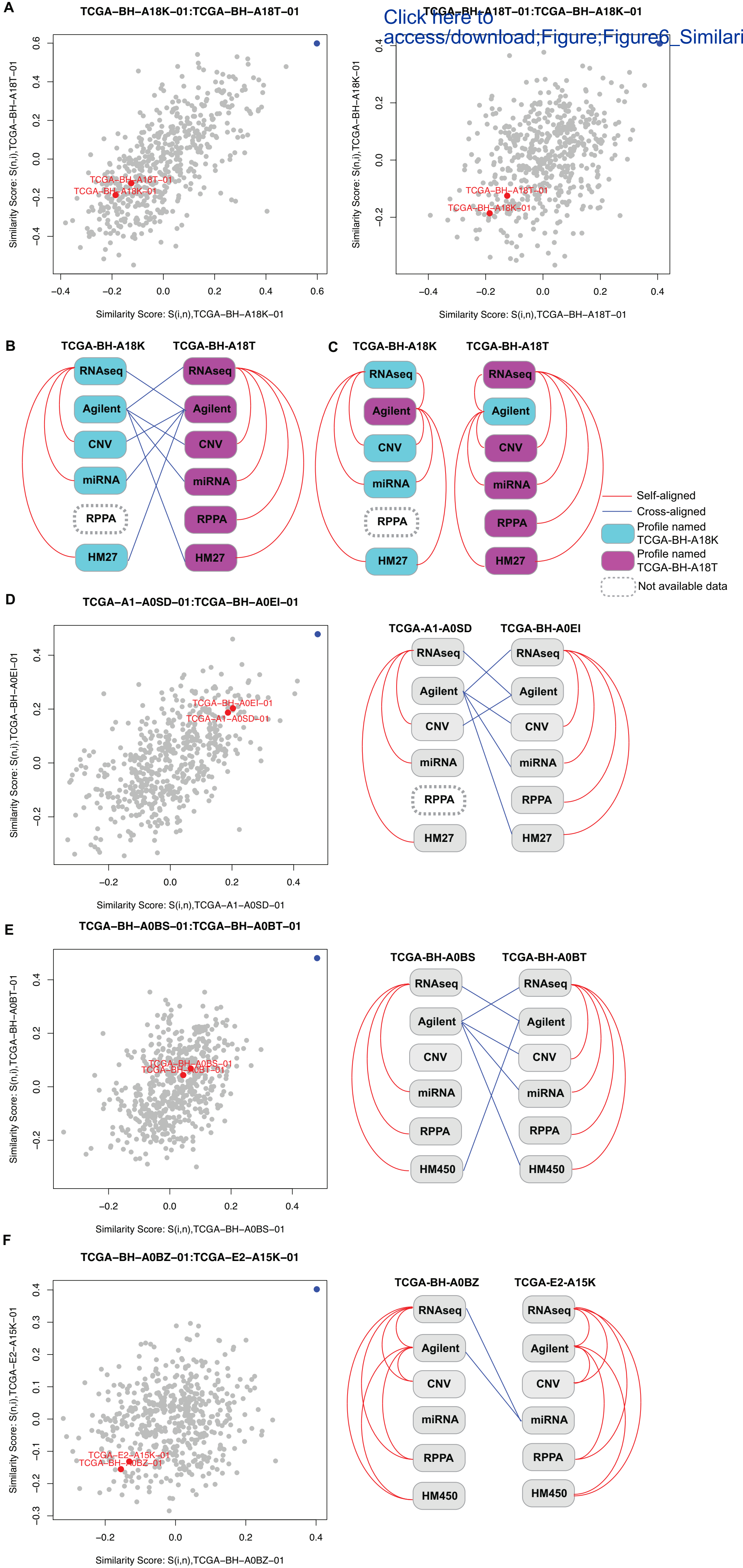

Figure 7

Click here to access/download;Figure;Figure7_RPPAalignments_01102019.e

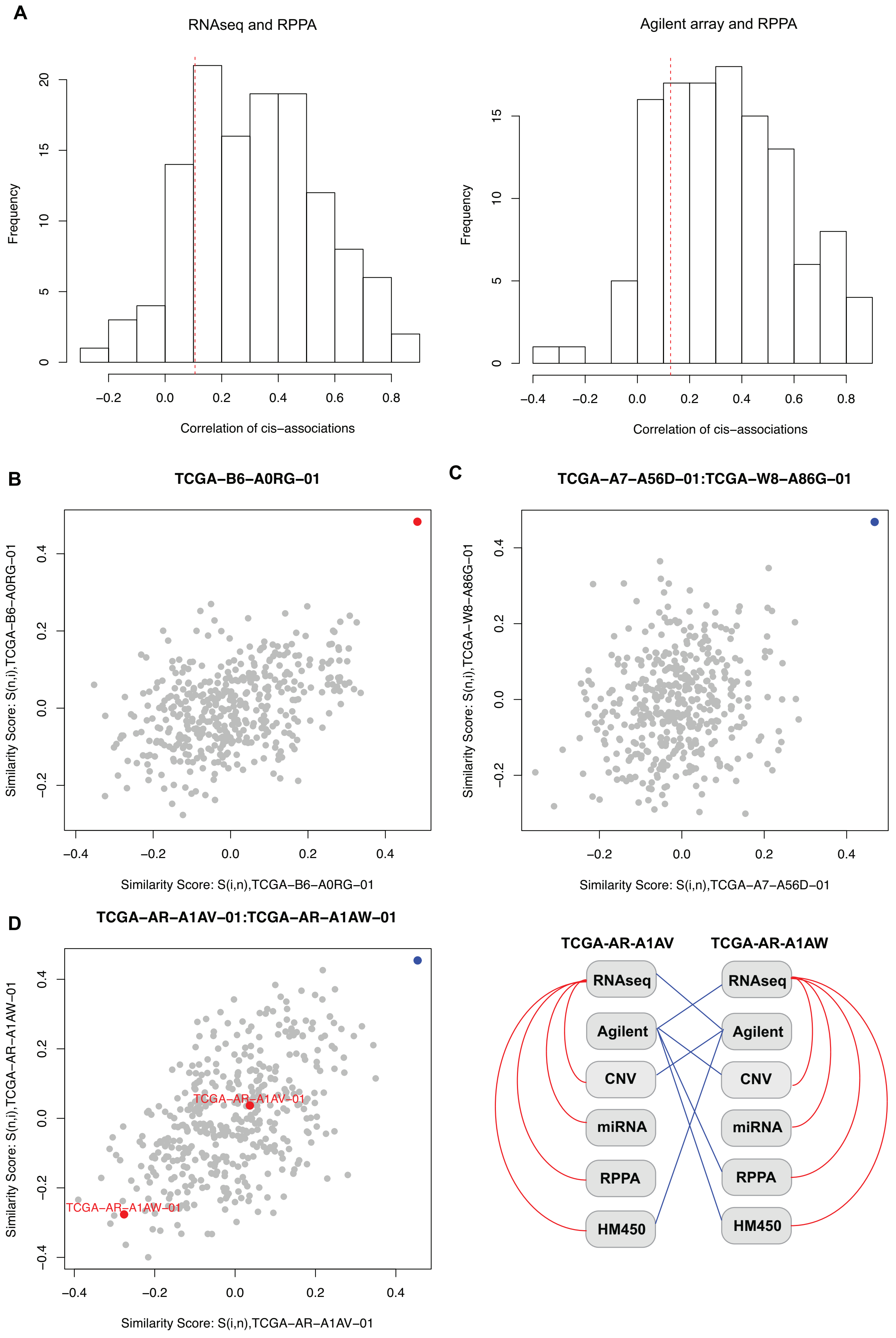

Figure 8

Click here to access/download;Figure;Figure8_CompareCorrelationAfterDisco

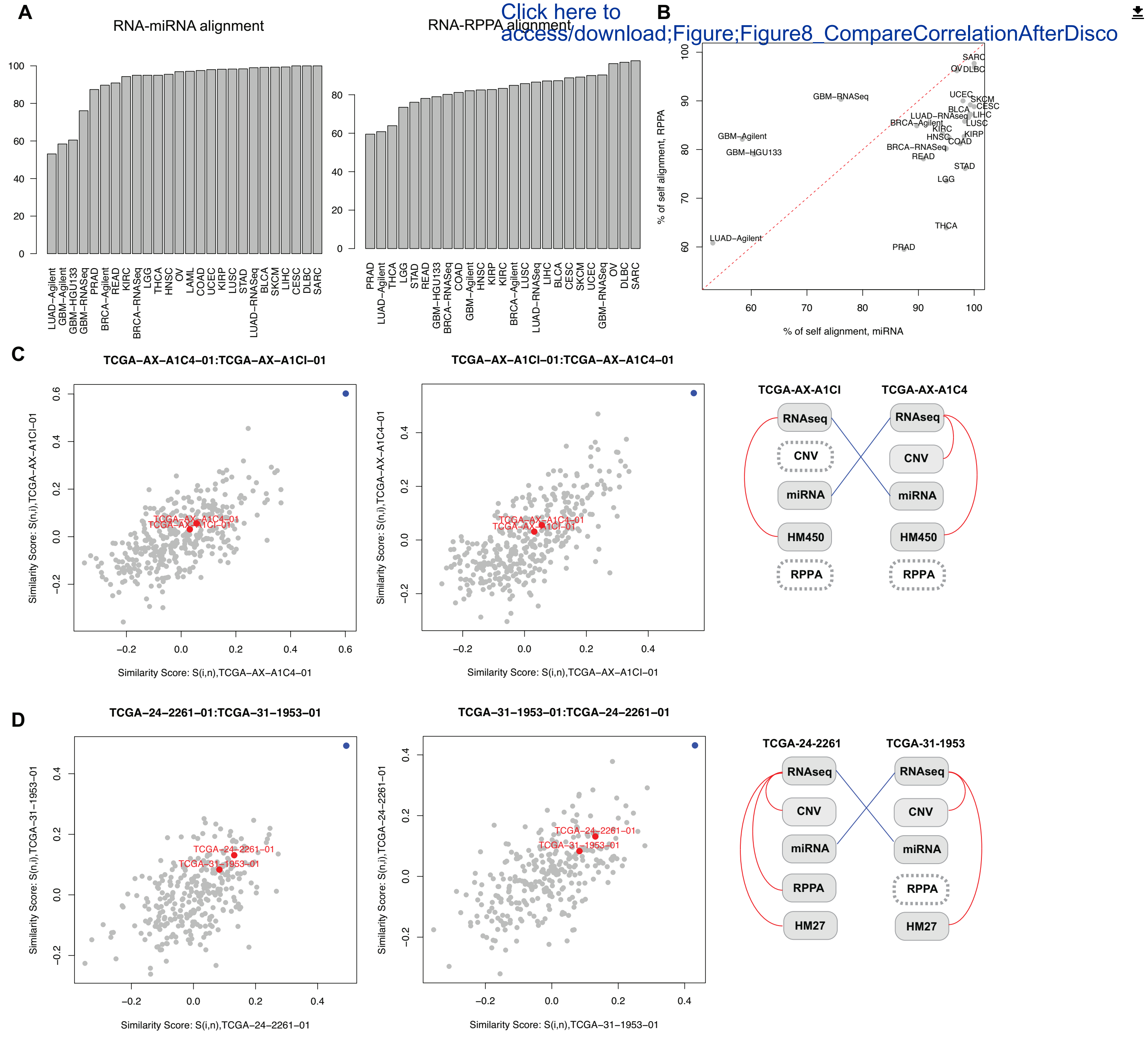

Figure 9

Click here to access/download;Figure;Figure9_ICGCexamples.eps

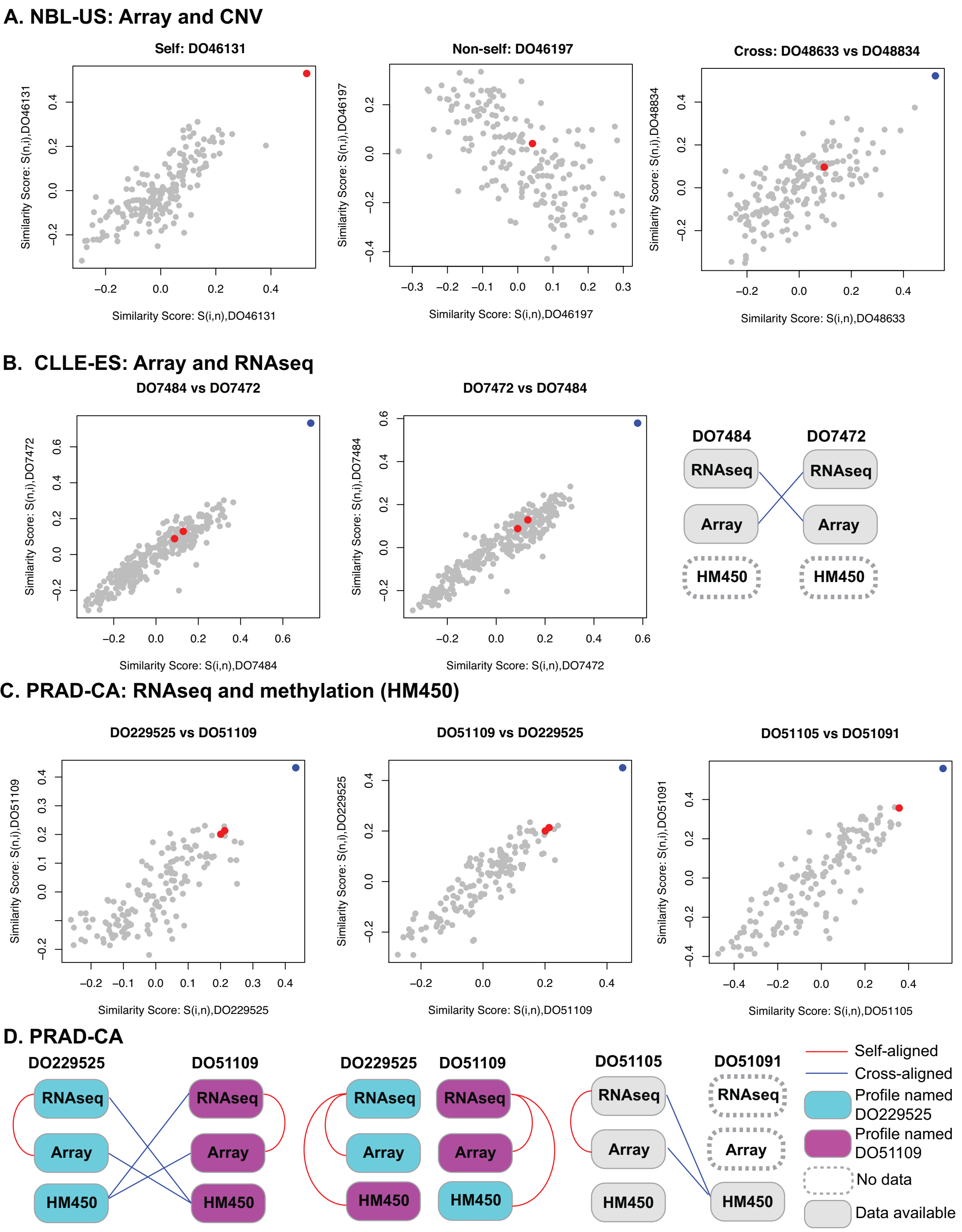

Figure 10

Click here to access/download;Figure;Figure10_OutlierandCisaftercorrection_

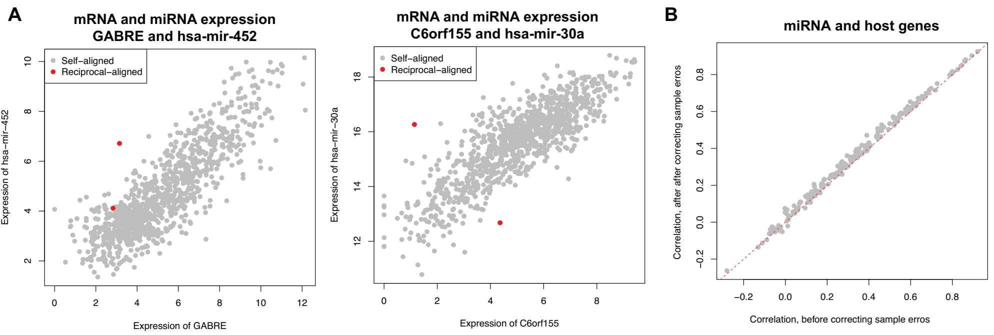

Click here to access/download
Supplementary Material
GigaScience_SupplementaryMaterials_Revision.pdf

Click here to access/download;Personal Cover;Response to Reviewer comments_v1_EL.docx

We have thoroughly addressed all reviewers' comments pertaining to our manuscript "*A probabilistic multi-omics data matching method for detecting sample errors in integrative analysis*". The reviewers' comments are very thoughtful and constructive, and have served to strengthen the manuscript significantly. We performed additional data analyses suggested by the reviewers, and revised the manuscript to address all comments as detailed below. The reviewer's comments are in black font type and our responses are given in blue. All page numbers and other such as references given are with respect to the revised manuscript unless otherwise stated.

Reviewer reports:

Reviewer #1: The author present proMODMatcher, a probabilistic multi-omics data matching method for detecting sample errors in integrative analysis. The study concerns the relevant problem of detecting sample errors in large datasets and the presented method offers an interesting solution. The method, which is an extension of MODMatcher, is designed to overcome the issue that the power of MODMatcher decreases when the number of "cis-associations" between two omics profiles is small. Overall, the paper is well organized.
We thank the reviewer for the encouraging comments.

I recommend a revision because better justification is needed for the arguments based on existing data and the clarity of some results needs to be improved.

1) The generic concept of "biological cis-association" should be explained in more detail and supported with some examples, starting with the introduction. Indeed, this concept is central to the functioning of both MODMatcher and proMODMatcher, and it is also related to the main motivation for the development of proMODMatcher. Besides, what are the criteria for defining such cis-associations? To which (combinations of) omics types can such criteria be applied?
We appreciated the reviewer's comment. Following the reviewer suggestion, we added the following sentences at the Introduction in Page 4:
"The main idea is first to identify "biological *cis*-associations" between two types of omics data, and then to use these "biological *cis*-associations" as intrinsic barcodes to match different types of omics data. The types of "biological *cis*-associations" are different when different pairs of omics data are mapped, but they all reflect general biological regulations. For example, when mapping genotype and gene expression data, the method is based on *cis*-genetic regulation of expression traits (or expression quantitative trait loci—*cis*-eQTLs), where a genetic polymorphism at a gene's promotor or regulatory region affects transcription factors or co-factors binding, which in turn affects the abundance of the gene's transcript [11]. Similarly, when mapping methylation and gene expression data, the method leverages on *cis*-methylation regulation of expression traits (or *cis*-methyls), where high DNA methylation level of CpGs at a gene's promotor or regulatory region hinders transcription factors or co-factors binding, which in turn represses the gene's transcription [12]. More on "biological *cis*-associations" are detailed in the Methods section. "

Also, we added the following section at the Methods section in Page 21:
"**<u>Biological cis-associations</u>**
"Biological cis-associations" reflect different biological regulations when different pairs of omics data are mapped. (1) cis-eQTLs for mapping genotype and gene expression data: a genetic polymorphism at a gene's promotor or regulatory region affects transcription factors or co-factors binding, which in turn affects the abundance of the gene's transcripts [11]. If the genetic polymorphism occurs within 1M bases from the gene's transcription start site and the

association is significant at the false discovery rate (FDR) <0.05, the association is called as a cis-eQTL. (2) cis-methylations for mapping DNA methylation and gene expression data: increased DNA methylation at CpGs sites near a gene promoter region is associated with gene repression [12]. A methylation probe is assigned to the transcript whose start site is closest to the genomic location of the methylation probe when it is potentially mapped to multiple transcripts. If a DNA methylation probe locates within 1M bases from the gene's start site and the association between the methylation level and the gene's expression level is significant at FDR <0.05, the methylation probe is a cis-methylation probe. (3) cis-CNVs for mapping DNA copy number variations (CNVs) and gene expression profiles: amplified or deleted genomic regions can regulate the expression levels of genes within that genomic region [16]. If a gene's expression is associated with its CNV at FDR <0.05, the CNV is a cis-CNV. (4) cis-miRNA-gene pairs for mapping miRNA and gene expression profiles: a small portion of miRNAs are embedded in gene regions (i.e. host genes) and frequently co-transcribed with host genes [14, 15]. If the expression levels of a miRNA and its host gene are associated at FDR <0.05, the pair is a cis-miRNA-gene pair. (5) cis-mRNA-protein pairs for mapping protein and gene expression profiles: the abundance of a protein depends on the corresponding mRNA transcript level and other factors [17]. If their association is significant at FDR <0.05, the pair is a cis-mRNA-protein pair."

2) Related to point 1: are there limitations in terms of missing data or sparse datasets (e.g. mutation profiles)?
For genotype data, we used common variants instead of rare variants to increase information content (Shannon Entropy) per locus (or gene). In the eQTL analyses, the loci of minor allele frequency (MAF)>0.05 were include.

Regarding missing data, we pre-processed data profiles to filter out genes or probes with more than 25% of missing values. Regarding sparse data, the input data can be any sparse datasets such as mutation profiles. We added the following sentences in the Discussion section in Page 19:
"The proMODMatcher depends on a set of biological cis-associations and the information content (Shannon entropy) of each cis-association depends on the randomness of each locus or gene. Thus, in our analyses, we excluded biological cis-associations that are driven by extreme values (rare events). For example, in eQTL analyses, we only included loci of minor allele frequency (MAF)>0.05. Missing values commonly occur in high throughput omics data. In our analyses, we don't explicitly impute missing values. Instead, we filtered out probes or genes of more than 25% missing value in the data pre-processing step."

3) In general, some aspects related to the comparison between proMODMatcher and MODmatcher should be clarified.

3.1) The difference between the performances of the two methods in simulated datasets is very narrow (mostly of 10^(-3) of 10^(-4), like 0.9994 vs 1). In this view, the improvement of proMODMatcher in comparison to MODMatcher appears to be very marginal. Additionally, the specificity for some simulations at low nCIS (e.g. red dots nCIS=75) is, in opposition to expectations, higher in MODMatcher than proMODMatcher; these results raise concerns on the expected superiority of proMODMatcher vs MODMatcher at low nCIS, which does not appear as clearly as in Figure 2.
Methods performance depends on both sensitivity and specificity. The *pro*MODMatcher method performed better than MODMatcher did in term of F scores (Figure 3B). The top goal of MODMatcher and *pro*MODMatcher is to detect "errors" of omics profiles without introducing any

errors. Therefore, we emphasized the improvement of *pro*MODMatcher in terms of specificity over sensitivity. Figure 3D shows that *pro*MODMatcher achieved better specificity than MODMatcher across all conditions that we tested, and the better F scores (Figure 3B) were largely due to better specificity. We reworded the paragraph clarify this point of view in the Analyses section in Page 9:
"The top goal of MODMatcher and proMODMatcher is to identify sample labeling errors without introducing any errors. Thus, we optimized the specificity of proMODMatcher over its sensitivity. In terms of sensitivity and specificity's contribution to F scores, proMODMatcher achieved a similar sensitivity as MODMatcher (Figure 3C) but better specificities in all cases (Figure 3D). "

3.2) In real datasets (TCGA), the gain of using proMODMatcher instead of MODMatcher is not clearly quantified. To better motivate the use of proMODMatcher in spite of MODMatcher, the authors should better illustrate the quantitative differences between the results obtained by the two methods. For instance, how many conflicting predictions? Shared results?
Following the reviewer's comments, we quantified the comparison of results for proMODMatcher and MODMatcher in real data sets in the Analyses section and added one additional column in Tables 1 and 2, indicating whether cross-aligned pairs were detected by MODMatcher.

Additionally, we added similarity score plots for the cross-aligned pairs that were detected only by MODMatcher as Supplementary Figure S2 and Supplementary Figure S4 to emphasize specificity of *pro*MODMatcher. Also, we added the following sentences in the *Aligning gene expression profiles by RNAseq and miRNAseq data* of Analyses section in Page 12:
"On the other hand, the cross-aligned pairs detected only by MODMatcher showed relatively marginal similarity scores even though the similarity scores of cross-aligned pairs were the highest (Supplementary Figure S2). "

Also, we added the following sentences in the *Aligning gene expression profiles by Agilent microarray and miRNAseq data* of Analyses section in Page 13:
"8 out of 9 pairs were also detected by MODMatcher (Table 1). MODMatcher detected additional cross-aligned pairs including several questionable cross-aligned pairs (i.e. TCGA−E2−A153−01 and TCGA−E9−A1NG−01, TCGA-AR-A1AL−01 and TCGA−AR−A1AN−01 in Supplementary Figure S4)."

Additionally, for the alignment between RPPA and Array profiles, we identified the cross-aligned pair of the mRNA Agilent microarray profile TCGA-AR-A1A**V**-01 and the RPPA profile of TCGA-AR-A1A**W**-01 data, consistent with labeling errors in the mRNA Agilent array data. However, this pair was not identified by MODMatcher (Table 2), indicating its limited sensitivity. We added the following sentences in the Application to TCGA breast cancer dataset: mRNA and RPPA profiles of Analyses section in Page 15:
"However, this pair was not identified by MODMatcher (**Table 2**)."

Other minor comments

It is important that potential users are aware of the computational cost required for the analyses.
Following the reviewer's comment, we added our computational cost and CPU time at the end of the Discussion section in Page 19:
"The computational cost of applying proMODMatcher is small. For example, mapping mRNA and miRNA expression profiles for 408 samples took 802 seconds of CPU time with maximum memory usage of 503 MB on a machine with CPU processor 3.50 GHz. ".

117 "based on"?
We thank the reviewer for pointing out these errors. Yes, it should be “based on”

355 Only here the author mention Pearson correlation. Did you mean Spearman?
Yes, it should be “Spearman correlation”.

382 RT(…) and T(…)
Yes , they should RT(..).

Fig. 1 caption: "calucalte"
We corrected the mis-spelling in the Fig1D’s caption.

Fig. 4d sothers
We corrected the mis-spelling.

Reviewer #2: Major comments:
1. It would be highly appreciated if the github or other open source (e.g. CRAN R-package) version of the tool can be provided with a user-friendly manual, this will help to make this tool available to a large enough community.
Following the reviewer’s comments, we uploaded our package to github (https://github.com/integrativenetworkbiology/proMODMatcher). It will become public once the paper is published.

2. It is not very clear how the proteomics/ CNV/ methylation are mapped to gene expression data. From the result part, I can only see RNAseq/microRNA/RPPA/microarray datasets. I didn't see the results of other multi-omics layers as introduced in the data description section of the results part.
As the reviewer suggested, we added the following sentences in the Introduction section in Page 4:
“The main idea is first to identify “biological *cis*-associations” between two types of omics data, and then to use these “biological *cis*-associations” as intrinsic barcodes to match different types of omics data. The types of “biological *cis*-associations” are different when different pairs of omics data are mapped, but they all reflect general biological regulations. For example, when mapping genotype and gene expression data, the method is based on *cis*-genetic regulation of expression traits (or expression quantitative trait loci—*cis*-eQTLs), where a genetic polymorphism at a gene’s promotor or regulatory region affects transcription factors or co-factors binding, which in turn affects the abundance of the gene’s transcript [11]. Similarly, when mapping methylation and gene expression data, the method leverages on *cis*-methylation regulation of expression traits (or *cis*-methyls), where high DNA methylation level of CpGs at a gene’s promotor or regulatory region hinders transcription factors or co-factors binding, which in turn represses the gene’s transcription [12]. More on “biological *cis*-associations” are detailed in the Methods section. ”

Also, we added the following section at the Methods section in Page 21:
“**Biological cis-associations**
“Biological cis-associations” reflect different biological regulations when different pairs of omics data are mapped. (1) cis-eQTLs for mapping genotype and gene expression data: a genetic polymorphism at a gene’s promotor or regulatory region affects transcription factors or co-factors binding, which in turn affects the abundance of the gene’s transcripts [11]. If the genetic

polymorphism occurs within 1M bases from the gene's transcription start site and the association is significant at the false discovery rate (FDR) <0.05, the association is called as a cis-eQTL. (2) cis-methylations for mapping DNA methylation and gene expression data: increased DNA methylation at CpGs sites near a gene promoter region is associated with gene repression [12]. A methylation probe is assigned to the transcript whose start site is closest to the genomic location of the methylation probe when it is potentially mapped to multiple transcripts. If a DNA methylation probe locates within 1M bases from the gene's start site and the association between the methylation level and the gene's expression level is significant at FDR <0.05, the methylation probe is a cis-methylation probe. (3) cis-CNVs for mapping DNA copy number variations (CNVs) and gene expression profiles: amplified or deleted genomic regions can regulate the expression levels of genes within that genomic region [16]. If a gene's expression is associated with its CNV at FDR <0.05, the CNV is a cis-CNV. (4) cis-miRNA-gene pairs for mapping miRNA and gene expression profiles: a small portion of miRNAs are embedded in gene regions (i.e. host genes) and frequently co-transcribed with host genes [14, 15]. If the expression levels of a miRNA and its host gene are associated at FDR <0.05, the pair is a cis-miRNA-gene pair. (5) cis-mRNA-protein pairs for mapping protein and gene expression profiles: the abundance of a protein depends on the corresponding mRNA transcript level and other factors [17]. If their association is significant at FDR <0.05, the pair is a cis-mRNA-protein pair."

3. Mapping database: I can just see a mapper file in the package which is between microRNA and gene expression. I don't know the resource of the mapping file, which should be described in the methods section. 4. This resource may also be updated regularly. The mapping file should also include methylation/gene expression, protein/gene expression etc. Currently this tool is not as what it declares to be, a "multi-omics tool".
Our mapping information is based on human genome assembly GRCh37 or gene symbols. We uploaded the following mapper files:

Matching_array_MethylationHM27.txt: Mapping between gene symbol and HM450 probe ID
Matching_array_MethylationHM450.txt: Mapping between gene symbol and HM27 probe ID
Matching_array_miRNA.txt: Mapping between gene symbol and miRNA
Matching_array_protein.txt: Mapping between gene symbol and RPPA protein

TCGA datasets are mostly based on U.S. patients, I am wondering if you can look into ICGC datasets (https://urldefense.proofpoint.com/v2/url?u=https-3A__dcc.icgc.org_projects&d=DwIGaQ&c=shNJtf5dKgNcPZ6Yh64b-A&r=RO09G907SbMLMqHyrCDZCw&m=HO91CP23G7b0TPBszNguttd47V51QT6Z7R7AQmyn-m8&s=e2XbBb6Lvod0C-R71wukkxsbIJ3yAUM5CrjPmWJXutQ&e=) to look into other multi-omics datasets and see if this tool still holds on the other datasets?
We thank the reviewer for the suggestion. We applied our procedure to ICGC datasets. Among ICGC datasets with more than one types of omics profiles (i.e. expression, DNA methylation, miRNA expression, and copy number variation profile) available, we selected 8 datasets based on the number of samples (i.e. more than 25). We added the section "ICGC datasets" in the Data Description section as follows:
"**ICGC datasets**
"For the ICGC datasets, the pre-processed data were downloaded from ICGC data portal (https://dcc.icgc.org/). We selected datasets with more than one available types of omics data including mRNA expression profiles (i.e. RNAseq and Array), DNA methylation profiles based on Illumina HumanMethylation450 (HM450), miRNA expression profiles, and copy number somatic mutation profiles. Each of profiles was reformatted into a matrix with genes (or probes)

as rows and barcodes of samples as columns. The gene and miRNA expression profiles were log2 transformed and normalized by quantile normalization[13]. For copy number somatic mutation profiles, the segments were mapped to hg19 gene symbols. Some datasets contain very sparse segment information for copy number somatic mutation profiles such as CLLE-ES. We excluded these copy number profiles for further analysis. For methylation profiles, the probes were mapped to hg19 gene symbols."

Among ICGC datasets, *pro*MODMatcher identified data errors in some of datasets including CLLE-ES and PRAD-CA. To summarize the results, we added the Table 5 and Figure 9 and the section Application to ICGC datasets in the Analyses section in Page 17:
"**Application to ICGC datasets**
We applied proMODMatcher to 8 cancer datasets that were generated by institutes in the U.S., Spain, UK, Germany, Australia, Canada, and France. Each dataset contains more than one types of omics data including mRNA expression profiles (i.e. RNAseq and Array), DNA methylation profiles based on Illumina HumanMethylation450 (HM450), miRNA expression profiles, and copy number somatic mutation profiles. The ICGC datasets used and the associated alignment results were summarized in Table 5. In some of datasets such as PAEN-AU and PRAD-FR, all profiles were matched to other corresponding profiles of the same sample names (Table 5). On the other hand, several sample errors were identified in some datasets. For example, mapping between gene expression Array and CNV profiles in the NBL-US dataset resulted in 170 self-self aligned sample pairs, 10 non self-self aligned samples and 12 cross-mapped pairs of profiles (examples shown in Figure 9A). Mapping gene expression profiles by RNAseq and Array in the CLLE-ES dataset yielded five non self-self aligned samples and two cross-mapped pairs of samples. The two cross-mapped pairs of samples were likely due to a swap of either RNAseq profile or Array profile (Figure 9B). Similarly, proMODMatcher identified three cross-alignments between RNAseq and DNA methylation profiles in the PRAD-CA dataset, which were also involved in cross-mappings when mapping Array and DNA methylation profiles: two of them were likely due to a swap of DNA methylation (HM450) profiles of DO229525 and DO51109 (Figure 9CD), and one of them was likely due to sample labeling errors in DNA methylation array (HM450) (Figure 9CD). "

Minor comments:
There were several instances in the manuscript where there were minor grammatical errors. I'd recommend just having a native English speaker give it a careful read before publication. Also there are some misspelling errors (eg. Figure 4E Correlation) in this paper.
There seems to be a bar omitted in Figure 3A first plot with nCis = 75, # sample =1000.
We thank the reviewer for pointing out these errors. We corrected misspelling errors and added a bar corresponding nCis=75 and # sample = 1000 in Figure 3A plot.

Click here to access/download;Personal Cover;GigaSciences_ReviewerReports2.docx

Reviewer reports:
Reviewer #1: The authors responded appropriately to my comments. The manuscript still requires some editing for language and clarity, such as:

- 417. "The sensitivity and accuracy of multi-omics profile matching 418 methods needs further improvement" should be "The sensitivity and accuracy […] need further improvement".
We thank the reviewer for pointing this out. We corrected the grammar error.

- 421. "The proMODMatcher depends on a set of biological cis-associations and the information content (Shannon entropy) of each cis-association depends on the randomness of each locus or gene".
Here, the "randomness" attributed to "each locus or gene" is unclear and requires further explanation.
As the reviewer suggested, we modified the sentence as the following :
"The *pro*MODMatcher depends on a set of biological *cis*-associations and the information content (Shannon entropy) of each *cis*-association depends on the randomness of genotypes at each locus or expression of each gene. For example, if there were two possible genotypes at a locus, then randomness or Shannon entropy is maximized when the probability of each genotype is 50%. If the probabilities of the two genotypes deviate from equal, the randomness or Shannon entropy at the locus decreases."

Reviewer #2: Most of the issues have been addressed.

One question regarding the package is regarding the resource of these mapping files, where are they coming from? Are they up-to-date? Are they all experiment validated?
For Methylation data, we downloaded annotation file for HM27 and HM450 Illumina BeadChip. For miRNA, based on the coordinates of genes and miRNA, we mapped miRNA-host genes. For protein, we mapped the protein whose gene symbol is same as the mRNA id. All mapping files are based on most updated coordinates in chromosome of genes and probes.. There is no experiment attempted to validate beyond associations.

It will be much better if you can provide the links for these files and offer an automatic way of updating, with standardized IDs for each category (gene expression, methylation, CNV, proteins etc.)
We thank the reviewer's suggestion. We modified the code and readme file to take standardized IDs and use the mapped files if a user prefers.
