# Supplementary material for: A probabilistic multi-omics data matching method for detecting sample errors in integrative analysis
Source: Gigascience. 2019 Jul 9;8(7):giz080. doi: 10.1093/gigascience/giz080 (PMC6615984; doi:10.1093/gigascience/giz080)
Supplement: giz080_Supplemental_File [file giz080_supplemental_file.pdf]

SUPPLEMENTARY MATERIALS

A probabilistic multi-omics data matching method for detecting sample errors in integrative analysis

Eunjee Lee[1,2,3], Seungyeul Yoo[1,2], Wenhui Wang[1,2], Zhidong Tu[1,2] and Jun Zhu[1,2,3,4,*]

[1]Department of Genetics and Genomic Sciences; [2]Icahn Institute of Genomics and Multiscale Biology, Icahn School of Medicine at Mount Sinai, New York, NY 10029; [3]Sema4, a Mount Sinai venture, Stamford, CT, USA; [4]The Tisch Cancer Institute, Icahn School of Medicine at Mount Sinai, New York, NY 10029;

*All corresponds should be addressed to Dr. Jun Zhu (jun.zhu@mssm.edu)

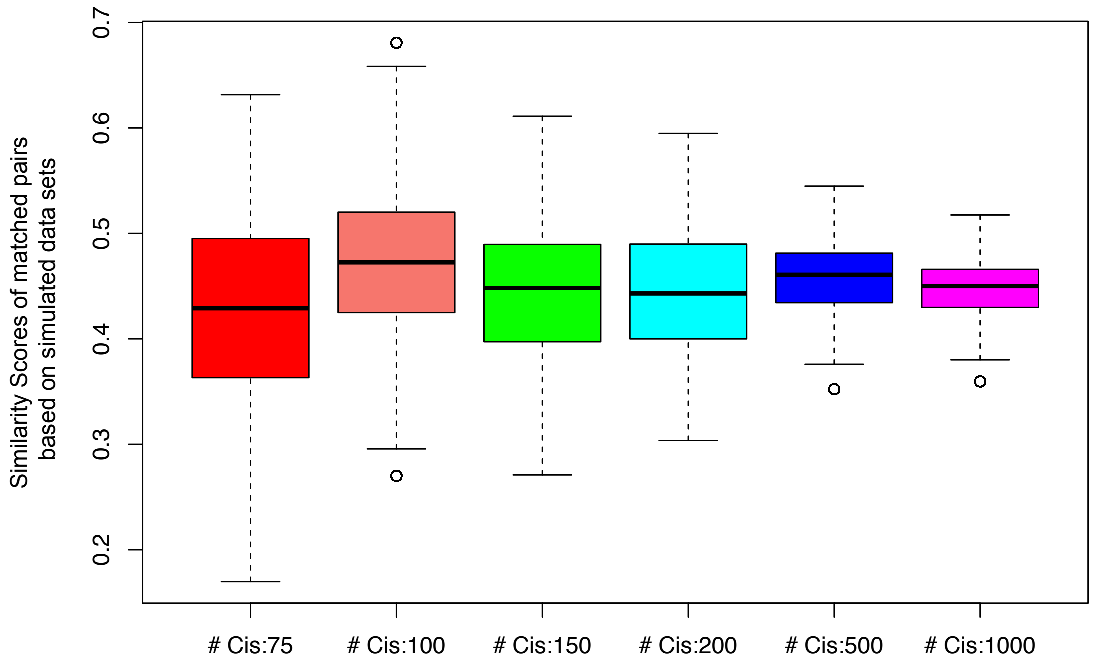

Supplementary Figure S1. Similarity scores of matched pairs based on simulated data sets.

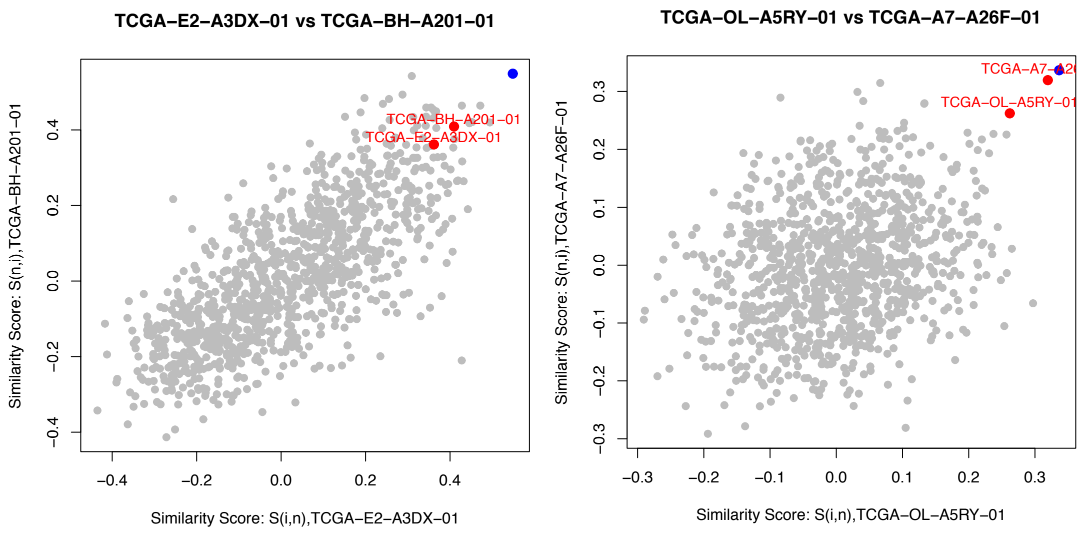

Supplementary Figure S2. Similarity scores of cross-aligned pairs detected by only MODMatcher based on aligning RNASeq and miRNA profiles of BRCA data.

A

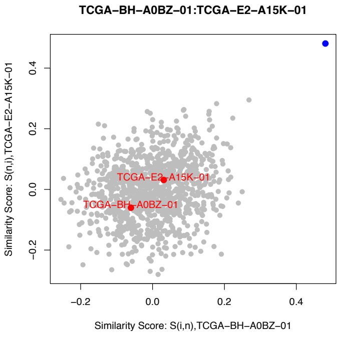

B

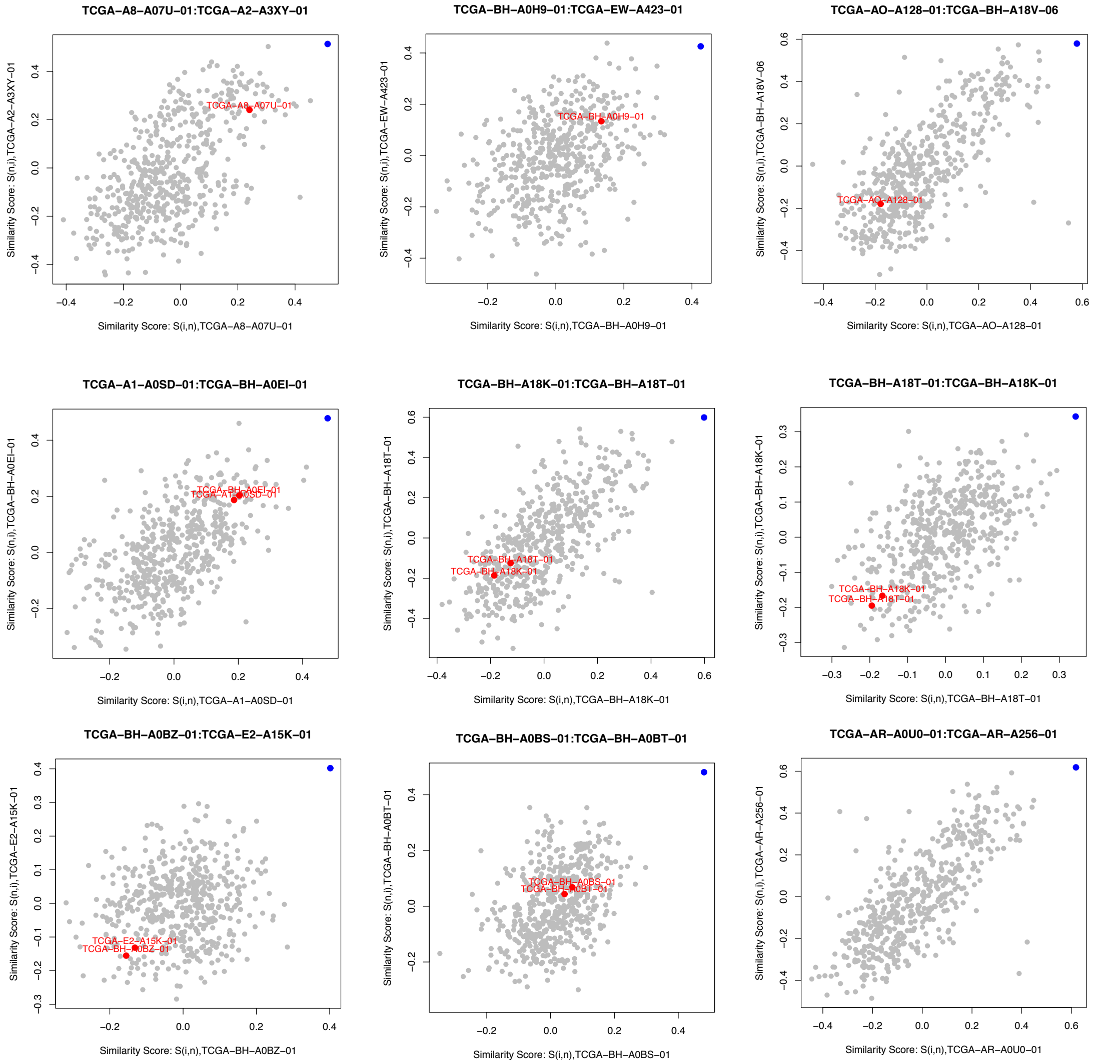

Supplementary Figure S3. Similarity scores of cross-aligned pairs based on aligning RNA and miRNA profiles of BRCA data. (A) Alignment of RNAseq and miRNAseq profiles. (B) Alignment of Agilent array and miRNAseq profiles.

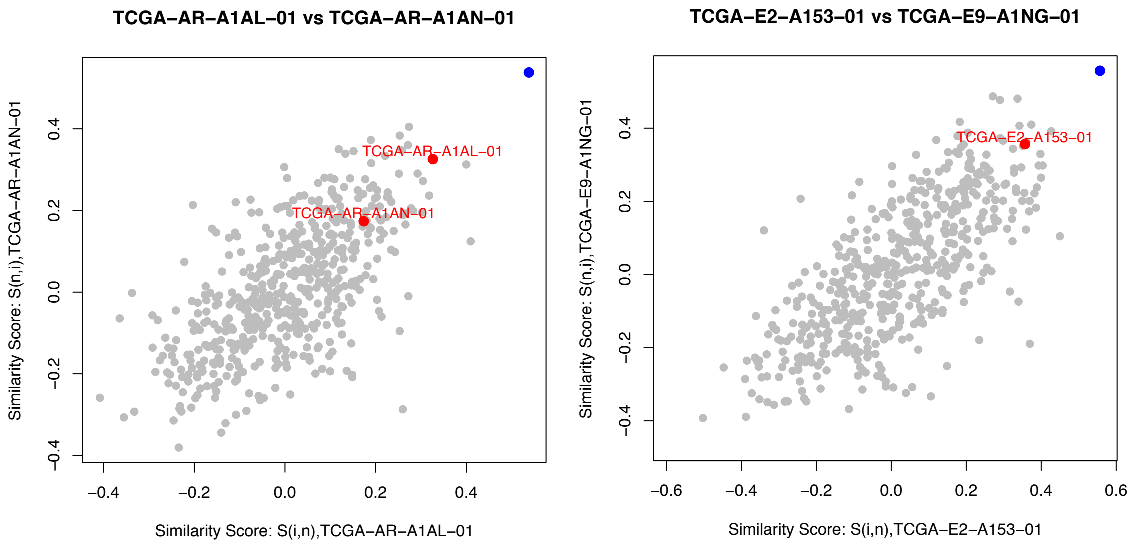

Supplementary Figure S4. Similarity scores of cross-aligned pairs detected by only MODMatcher based on aligning Array and miRNA profiles of BRCA data.

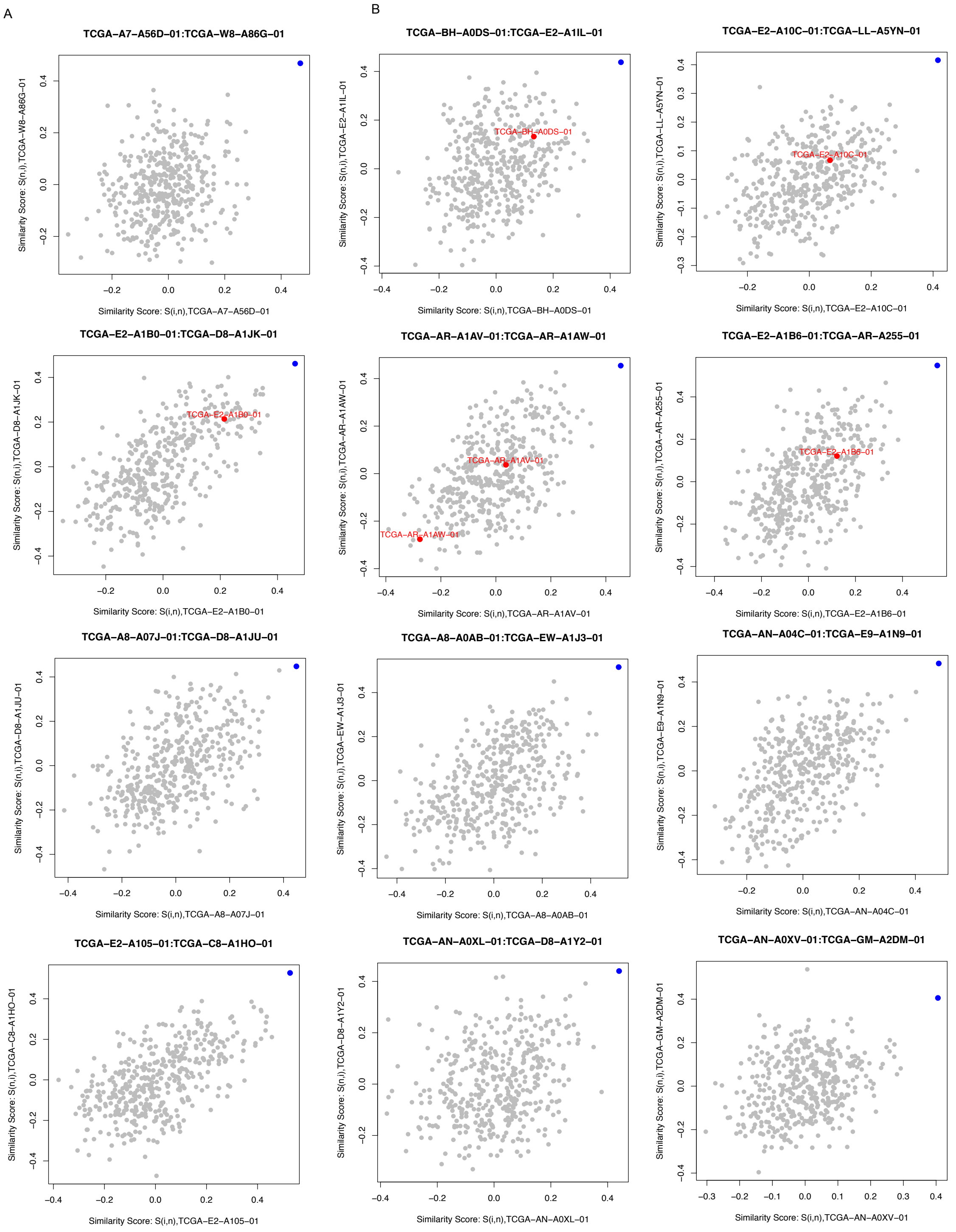

Supplementary Figure S5. Similarity scores of cross-aligned pairs based on aligning RNA and RPPA profiles of BRCA data. (A) Aligning of RNAseq and RPPA profiles. (B) Aligning of Agilent array and RPPA profiles.

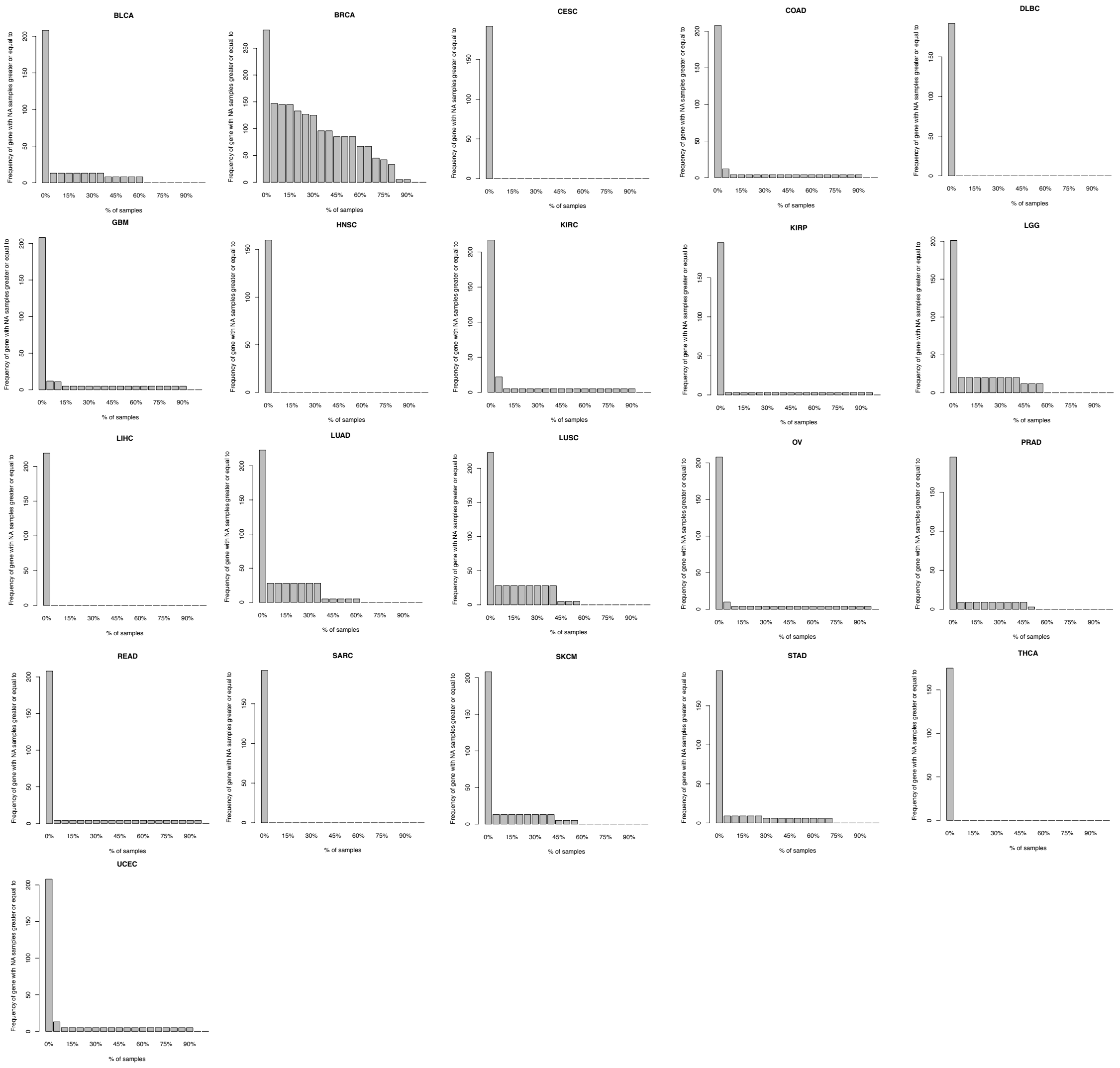

Supplementary Figure S6. The frequency of genes of which the number of samples with non-assigned values are greater than each % of samples.

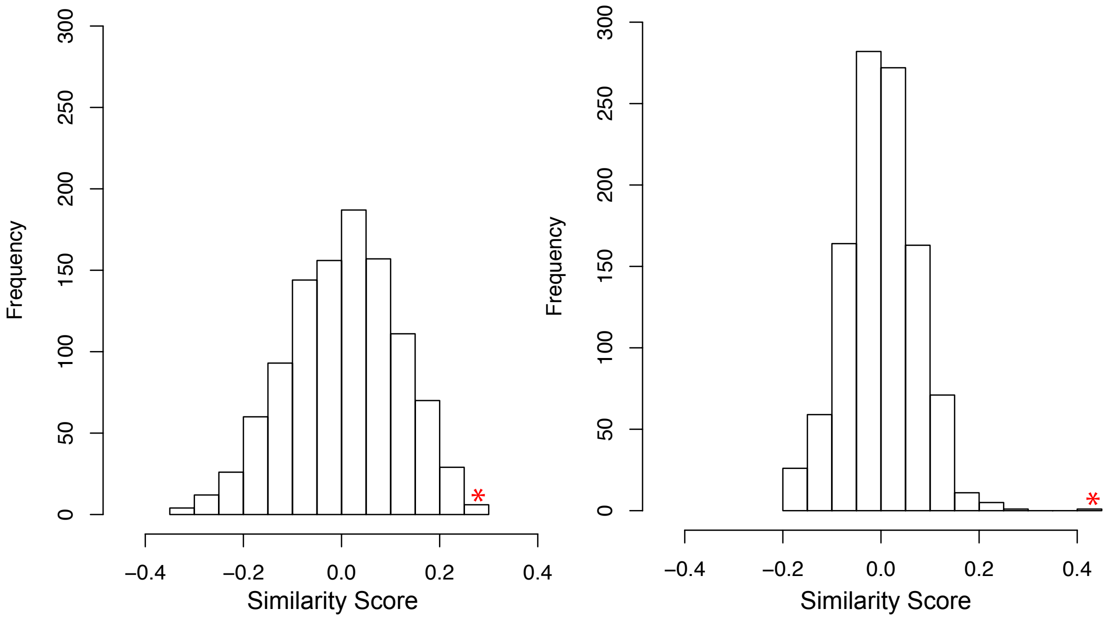

Supplementary Figure S7. Examples of distribution of similarity scores. The red star indicates the values of the highest similarity score.

Supplementary Table S1. Sample alignment results by MODMatcher for mRNA (Array and RNAseq) -miRNAseq profiles based on BRCA data

Method	Data types	Data types	# Common samples	# cis pair	# of self-aligned	# of cross-aligned	Cross-aligned pairs	Cross-aligned pairs
	Type1	Type 2	Type 1				Type 1	Type 2
MODMatcher	RNAseq	miRNAseq	1041	178/215	898 (86.2%)	5 (0.4%)	TCGA-PE-A5DC	TCGA-BH-A0B1
							TCGA-OL-A5RY	TCGA-A7-A26F
							TCGA-E2-A3DX	TCGA-BH-A201
							TCGA-BH-A0BZ	TCGA-E2-A15K
							TCGA-GM-A2DA	TCGA-E2-A14V
MODMatcher	Agilent	miRNAseq	519	137/178	452 (87.0%)	20 (3.8%)	TCGA-A8-A07U	TCGA-A2-A3XY
							TCGA-AN-A0FN	TCGA-D8-A1JH
							TCGA-AO-A0JF	TCGA-BH-A0DV
							TCGA-A2-A0T6	TCGA-D8-A73X
							TCGA-E2-A107	TCGA-A2-A25B
							TCGA-AO-A128	TCGA-BH-A18V
							TCGA-B6-A0X7	TCGA-AO-A128
							TCGA-AN-A0XV	TCGA-E9-A22B
							TCGA-A1-A0SD	TCGA-BH-A0EI
							TCGA-BH-A18K	TCGA-BH-A18T
							TCGA-BH-A18T	TCGA-BH-A18K
							TCGA-E2-A153	TCGA-E9-A1NG
							TCGA-BH-A0BZ	TCGA-E2-A15K
							TCGA-BH-A0AU	TCGA-A8-A0A7
							TCGA-AR-A1AL	TCGA-AR-A1AN
							TCGA-BH-A0BS	TCGA-BH-A0BT
							TCGA-E2-A1B5	TCGA-E2-A1LG
							TCGA-A2-A0YJ	TCGA-AR-A251
							TCGA-AR-A0TU	TCGA-OL-A66I
							TCGA-AR-A0U0	TCGA-AR-A256

Supplementary Table S2. Sample alignment results by MODMatcher for mRNA (Array and RNAseq) -RPPA profiles based on BRCA data

Method	Data types	Data types	# Common samples	# cis pair	# of self-aligned	# of cross-aligned	Cross-aligned pairs	Cross-aligned pairs
MODMatcher	RNAseq	RPPA	856	103/151	606 (70.7%)	26 (3.0%)	TCGA-AO-A12E-01	TCGA-EW-A1IX-01
							TCGA-AR-A24O-01	TCGA-C8-A26W-01
							TCGA-A2-A0CQ-01	TCGA-D8-A73U-01
							TCGA-EW-A6SC-01	TCGA-A8-A09K-01
							TCGA-GM-A2DK-01	TCGA-A8-A06N-01
							TCGA-E2-A1IU-01	TCGA-BH-A0HX-01
							TCGA-A2-A04Y-01	TCGA-EW-A1PA-01
							TCGA-GM-A3XG-01	TCGA-BH-A0HN-01
							TCGA-GM-A5PV-01	TCGA-AO-A0JC-01
							TCGA-D8-A73W-01	TCGA-E9-A1R3-01
							TCGA-AC-A5XS-01	TCGA-C8-A274-01
							TCGA-A2-A0YD-01	TCGA-A7-A26E-01
							TCGA-BH-A0EA-01	TCGA-AR-A24O-01
							TCGA-A2-A04Q-01	TCGA-AO-A128-01
							TCGA-LD-A7W6-01	TCGA-E2-A15M-01
							TCGA-A2-A04X-01	TCGA-3C-AALI-01
							TCGA-AC-A2FK-01	TCGA-AR-A2LJ-01
							TCGA-AC-A23H-01	TCGA-C8-A8HQ-01
							TCGA-A7-A56D-01	TCGA-W8-A86G-01
							TCGA-AC-A3EH-01	TCGA-GM-A5PX-01
							TCGA-E2-A570-01	TCGA-GM-A3XN-01
							TCGA-BH-A0HB-01	TCGA-LD-A7W5-01
							TCGA-BH-A0GZ-01	TCGA-E2-A15F-01
							TCGA-EW-A1IY-01	TCGA-A2-A25A-01
							TCGA-A8-A07G-01	TCGA-C8-A1HE-01
							TCGA-OK-A5Q2-01	TCGA-BH-A1EX-01
MODMatcher	Agilent	RPPA	424	97/145	347 (81.8%)	32 (7.5%)	TCGA-A8-A084-01	TCGA-PE-A5DD-01
							TCGA-BH-A0BA-01	TCGA-A7-A26H-01
							TCGA-BH-A0DS-01	TCGA-E2-A1IL-01
							TCGA-BH-A0DP-01	TCGA-AR-A24P-01
							TCGA-BH-A0HX-01	TCGA-EW-A1OY-01
							TCGA-BH-A0RX-01	TCGA-E2-A1LL-01
							TCGA-E2-A10C-01	TCGA-LL-A5YN-01

							TCGA-A2-A0YD-01	TCGA-BH-A1FB-01
							TCGA-E2-A14T-01	TCGA-E9-A1NI-01
							TCGA-BH-A0C1-01	TCGA-A2-A25C-01
							TCGA-BH-A18T-01	TCGA-BH-A0BQ-01
							TCGA-BH-A18F-01	TCGA-A2-A25A-01
							TCGA-C8-A133-01	TCGA-GM-A2DM-01
							TCGA-AR-A1AO-01	TCGA-A7-A26G-01
							TCGA-E2-A1B0-01	TCGA-D8-A1JK-01
							TCGA-E2-A1B1-01	TCGA-OL-A5RZ-01
							TCGA-AR-A1AL-01	TCGA-EW-A1IX-01
							TCGA-E2-A1B6-01	TCGA-E9-A1R0-01
							TCGA-A8-A08B-01	TCGA-D8-A1J9-01
							TCGA-AN-A0FG-01	TCGA-AR-A250-01
							TCGA-A8-A09V-01	TCGA-A2-A0YD-01
							TCGA-A8-A0AB-01	TCGA-A7-A3IZ-01
							TCGA-AO-A03R-01	TCGA-A2-A25D-01
							TCGA-A8-A07G-01	TCGA-C8-A1HE-01
							TCGA-B6-A0I5-01	TCGA-D8-A1X7-01
							TCGA-AO-A0J4-01	TCGA-D8-A27M-01
							TCGA-BH-A0H0-01	TCGA-BH-A1FJ-01
							TCGA-A2-A0EN-01	TCGA-EW-A1P1-01
							TCGA-E2-A105-01	TCGA-C8-A1HO-01
							TCGA-B6-A0WS-01	TCGA-BH-A0B6-01
							TCGA-BH-A18I-01	TCGA-AC-A3W7-01
							TCGA-BH-A0DO-01	TCGA-AR-A1AL-01
