# Supplementary material for: A probabilistic multi-omics data matching method for detecting sample errors in integrative analysis
Source: Gigascience. 2019 Jul 9;8(7):giz080. doi: 10.1093/gigascience/giz080 (PMC6615984; doi:10.1093/gigascience/giz080)
Supplement: giz080_Response_to_Reviewer_Comments_Original_Submission [file giz080_response_to_reviewer_comments_original_submission.pdf]

Author's Response To Reviewer Comments

Close

We have thoroughly addressed all reviewers’ comments pertaining to our manuscript “A probabilistic multi-omics data matching method for detecting sample errors in integrative analysis”. Detailed responses are listed in the "Response to reviewers comments" letter.

Close
