# Supplementary material for: A probabilistic multi-omics data matching method for detecting sample errors in integrative analysis
Source: Gigascience. 2019 Jul 9;8(7):giz080. doi: 10.1093/gigascience/giz080 (PMC6615984; doi:10.1093/gigascience/giz080)
Supplement: giz080_Response_to_Reviewer_Comments_Revision_1 [file giz080_response_to_reviewer_comments_revision_1.pdf]

Author's Response To Reviewer Comments

Close

Reviewer reports:
Reviewer #1: The authors responded appropriately to my comments. The manuscript still requires some editing for language and clarity, such as:

- 417. "The sensitivity and accuracy of multi-omics profile matching 418 methods needs further improvement" should be "The sensitivity and accuracy [...] need further improvement".
We thank the reviewer for pointing this out. We corrected the grammar error.

- 421. "The proMODMatcher depends on a set of biological cis-associations and the information content (Shannon entropy) of each cis-association depends on the randomness of each locus or gene".
Here, the "randomness" attributed to "each locus or gene" is unclear and requires further explanation.
As the reviewer suggested, we modified the sentence as the following :
"The proMODMatcher depends on a set of biological cis-associations and the information content (Shannon entropy) of each cis-association depends on the randomness of genotypes at each locus or expression of each gene. For example, if there were two possible genotypes at a locus, then randomness or Shannon entropy is maximized when the probability of each genotype is 50%. If the probabilities of the two genotypes deviate from equal, the randomness or Shannon entropy at the locus decreases."

Reviewer #2: Most of the issues have been addressed.

One question regarding the package is regarding the resource of these mapping files, where are they coming from? Are they up-to-date? Are they all experiment validated?
For Methylation data, we downloaded annotation file for HM27 and HM450 Illumina BeadChip. For miRNA, based on the coordinates of genes and miRNA, we mapped miRNA-host genes. For protein, we mapped the protein whose gene symbol is same as the mRNA id. All mapping files are based on most updated coordinates in chromosome of genes and probes.. There is no experiment attempted to validate beyond associations.

It will be much better if you can provide the links for these files and offer an automatic way of updating, with standardized IDs for each category (gene expression, methylation, CNV, proteins etc.)
We thank the reviewer's suggestion. We modified the code and readme file to take standardized IDs and use the mapped files if a user prefers.

Close
