# Supplementary material for: A probabilistic multi-omics data matching method for detecting sample errors in integrative analysis
Source: Gigascience. 2019 Jul 9;8(7):giz080. doi: 10.1093/gigascience/giz080 (PMC6615984; doi:10.1093/gigascience/giz080)
Supplement: giz080_Reviewer_1_Report_Original_Submission — Ettore Mosca -- 3/13/2019 Reviewed [file giz080_reviewer_1_report_original_submission.pdf]

**Reviewer Report**

**Title: A probabilistic multi-omics data matching method for detecting sample errors in integrative analysis**

**Version: Original Submission** **Date:** 3/13/2019

**Reviewer name: Ettore Mosca**

**Reviewer Comments to Author:**

The author present proMODMatcher, a probabilistic multi-omics data matching method for detecting sample errors in integrative analysis. The study concerns the relevant problem of detecting sample errors in large datasets and the presented method offers an interesting solution. The method, which is an extension of MODMatcher, is designed to overcome the issue that the power of MODMatcher decreases when the number of "cis-associations" between two omics profiles is small. Overall, the paper is well organized.â€¨
I recommend a revision because better justification is needed for the arguments based on existing data and the clarity of some results needs to be improved.â€¨â€¨
1) The generic concept of "biological cis-association" should be explained in more detail and supported with some examples, starting with the introduction. Indeed, this concept is central to the functioning of both MODMatcher and proMODMatcher, and it is also related to the main motivation for the development of proMODMatcher. Besides, what are the criteria for defining such cis-associations? To which (combinations of) omics types can such criteria be applied?
â€¨â€¨2) Related to point 1: are there limitations in terms of missing data or sparse datasets (e.g. mutation profiles)?
â€¨â€¨3) In general, some aspects related to the comparison between proMODMatcher and MODmatcher should be clarified.â€¨
3.1) The difference between the performances of the two methods in simulated datasets is very narrow (mostly of 10^(-3) of 10^(-4), like 0.9994 vs 1). In this view, the improvement of proMODMatcher in comparison to MODMatcher appears to be very marginal. Additionally, the specificity for some simulations at low nCIS (e.g. red dots nCIS=75) is, in opposition to expectations, higher in MODMatcher than proMODMatcher; these results raise concerns on the expected superiority of proMODMatcher vs MODMatcher at low nCIS, which does not appear as clearly as in Figure 2.â€¨â€¨
3.2) In real datasets (TCGA), the gain of using proMODMatcher instead of MODMatcher is not clearly quantified. To better motivate the use of proMODMatcher in spite of MODMatcher, the authors should better illustrate the quantitative differences between the results obtained by the two methods. For instance, how many conflicting predictions? Shared results?
â€¨â€¨Other minor commentsâ€¨
It is important that potential users are aware of the computational cost required for the analyses.â€¨
117 "based on"?â€¨355 Only here the author mention Pearson correlation. Did you mean Spearman?
â€¨382 RT(…) and T(…)â€¨

Fig. 1 caption: "calucalte"
â€¨Fig. 4d sothers

**Methods**

Are the methods appropriate to the aims of the study, are they well described, and are necessary controls included? Choose an item.

**Conclusions**

Are the conclusions adequately supported by the data shown? Choose an item.

**Reporting Standards**

Does the manuscript adhere to the journal's guidelines on minimum standards of reporting? Choose an item.

Choose an item.

**Statistics**

Are you able to assess all statistics in the manuscript, including the appropriateness of statistical tests used? Choose an item.

**Quality of Written English**

Please indicate the quality of language in the manuscript: Choose an item.

**Declaration of Competing Interests**

Please complete a declaration of competing interests, considering the following questions:

- Have you in the past five years received reimbursements, fees, funding, or salary from an organisation that may in any way gain or lose financially from the publication of this manuscript, either now or in the future?
- Do you hold any stocks or shares in an organisation that may in any way gain or lose financially from the publication of this manuscript, either now or in the future?
- Do you hold or are you currently applying for any patents relating to the content of the manuscript?
- Have you received reimbursements, fees, funding, or salary from an organization that holds or has applied for patents relating to the content of the manuscript?
- Do you have any other financial competing interests?
- Do you have any non-financial competing interests in relation to this paper?

If you can answer no to all of the above, write 'I declare that I have no competing interests' below. If your reply is yes to any, please give details below.

I declare that I have no competing interests.

I agree to the open peer review policy of the journal. I understand that my name will be included on my report to the authors and, if the manuscript is accepted for publication, my named report including any attachments I upload will be posted on the website along with the authors' responses. I agree for my report to be made available under an Open Access Creative Commons CC-BY license (http://creativecommons.org/licenses/by/4.0/). I understand that any comments which I do not wish to be included in my named report can be included as confidential comments to the editors, which will not be published.

I agree to the open peer review policy of the journal

To further support our reviewers, we have joined with Publons, where you can gain additional credit to further highlight your hard work (see: https://publons.com/journal/530/gigascience). On publication of this paper, your review will be automatically added to Publons, you can then choose whether or not to claim your Publons credit. I understand this statement.

Yes Choose an item.
