# Supplementary material for: A probabilistic multi-omics data matching method for detecting sample errors in integrative analysis
Source: Gigascience. 2019 Jul 9;8(7):giz080. doi: 10.1093/gigascience/giz080 (PMC6615984; doi:10.1093/gigascience/giz080)
Supplement: giz080_Reviewer_2_Report_Original_Submission — Sijia Huang -- 4/16/2019 Reviewed [file giz080_reviewer_2_report_original_submission.pdf]

Reviewer Report

Title: A probabilistic multi-omics data matching method for detecting sample errors in integrative analysis

Version: Original Submission **Date:** 4/16/2019

Reviewer name: Sijia Huang

Reviewer Comments to Author:

Major comments:
1. It would be highly appreciated if the github or other open source (e.g. CRAN R-package) version of the tool can be provided with a user-friendly manual, this will help to make this tool available to a large enough community.
2. It is not very clear how the proteomics/ CNV/ methylation are mapped to gene expression data. From the result part, I can only see
RNAseq/microRNA/RPPA/microarray datasets. I didn't see the results of other multi-omics layers as introduced in the data description section of the results part.
3. Mapping database: I can just see a mapper file in the package which is between microRNA and gene expression. I don't know the resource of the mapping file, which should be described in the methods section. 4. This resource may also be updated regularly. The mapping file should also include methylation/gene expression, protein/gene expression etc. Currently this tool is not as what it declares to be, a "multi-omics tool".
TCGA datasets are mostly based on U.S. patients, I am wondering if you can look into ICGC datasets (https://dcc.icgc.org/projects) to look into other multi-omics datasets and see if this tool still holds on the other datasets?
Minor comments:
There were several instances in the manuscript where there were minor grammatical errors. I'd recommend just having a native English speaker give it a careful read before publication. Also there are some misspelling errors (eg. Figure 4E Correlation) in this paper.
There seems to be a bar omitted in Figure 3A first plot with nCis = 75, # sample =1000.

Methods

Are the methods appropriate to the aims of the study, are they well described, and are necessary controls included? Choose an item.

Conclusions

Are the conclusions adequately supported by the data shown? Choose an item.

Reporting Standards

Does the manuscript adhere to the journal's guidelines on minimum standards of reporting? Choose an item.

Choose an item.

Statistics

Are you able to assess all statistics in the manuscript, including the appropriateness of statistical tests used? Choose an item.

Quality of Written English

Please indicate the quality of language in the manuscript: Choose an item.

Declaration of Competing Interests

Please complete a declaration of competing interests, considering the following questions:

- Have you in the past five years received reimbursements, fees, funding, or salary from an organisation that may in any way gain or lose financially from the publication of this manuscript, either now or in the future?
- Do you hold any stocks or shares in an organisation that may in any way gain or lose financially from the publication of this manuscript, either now or in the future?
- Do you hold or are you currently applying for any patents relating to the content of the manuscript?
- Have you received reimbursements, fees, funding, or salary from an organization that holds or has applied for patents relating to the content of the manuscript?
- Do you have any other financial competing interests?
- Do you have any non-financial competing interests in relation to this paper?

If you can answer no to all of the above, write 'I declare that I have no competing interests' below. If your reply is yes to any, please give details below.

I declare that I have no competing interests.

I agree to the open peer review policy of the journal. I understand that my name will be included on my report to the authors and, if the manuscript is accepted for publication, my named report including any attachments I upload will be posted on the website along with the authors' responses. I agree for my report to be made available under an Open Access Creative Commons CC-BY license (http://creativecommons.org/licenses/by/4.0/). I understand that any comments which I do not wish to be included in my named report can be included as confidential comments to the editors, which will not be published.

I agree to the open peer review policy of the journal

To further support our reviewers, we have joined with Publons, where you can gain additional credit to further highlight your hard work (see: https://publons.com/journal/530/gigascience). On publication of this paper, your review will be automatically added to Publons, you can then choose whether or not to claim your Publons credit. I understand this statement.

Yes Choose an item.
